# Priming Effects on Soil Organic Matter Mineralization by Carbon Substrates: A Global Meta‐Analysis

**DOI:** 10.1111/gcb.70861

**Published:** 2026-04-10

**Authors:** Hongxin Dong, Yingyi Fu, Shanshan Dai, Peng He, Yu Luo, Margaret A. Oliver, William R. Horwath, Lu‐Jun Li

**Affiliations:** ^1^ State Key Laboratory of Black Soils Conservation and Utilization, Northeast Institute of Geography and Agroecology Chinese Academy of Sciences Harbin China; ^2^ College of Advanced Agricultural Sciences University of Chinese Academy of Sciences Beijing China; ^3^ Institute of Soil and Water Resources and Environmental Science, Zhejiang Provincial Key Laboratory of Agricultural Resources and Environment Zhejiang University Hangzhou China; ^4^ Department of Geography & Environmental Science University of Reading Reading UK; ^5^ Department of Land, Air, Water and Resources University of California Davis California USA

**Keywords:** carbon cycling, carbon dioxide, carbon types, isotope tracing, litter quality, soil organic carbon

## Abstract

Priming effects (PE) on soil organic matter (SOM) mineralization depend strongly on the type of carbon substrates added. It is crucial to understand the PE induced by various carbon substrates for predicting SOM dynamics and soil‐atmosphere carbon feedback. We conducted a meta‐analysis of 8015 observations from 283 articles to evaluate how carbon substrates (plant residues, root exudates, biochar, and degradable microplastics) regulate the mineralization of SOM through PE. Results demonstrated that all these types of carbon substrates increased SOM mineralization, which induced a positive PE. Plant residues induced the highest average PE, followed by root exudates, biochar, and degradable microplastics. Compared to soils without carbon substrate inputs, the rate of SOM mineralization increased by 113% in soil with cellulose‐rich non‐woody residues, whereas it increased by only 25% in soil with lignin‐rich woody residues. The mineralization of SOM increased by organic acids (151%) was greatest in root exudates, followed by monosaccharides (60%) and polysaccharides (12%). The strong mineralization induced by organic acids was probably related to the release of more mineral nutrients by reducing soil pH. The PE on SOM mineralization by woody biochar with high aromatic carbon content (48%) was greater than that of non‐woody biochar with high alkyl carbon content (43%). Microplastics with rapidly degradable polyhydroxyalkanoates induced more SOM mineralization (258%) than polybutylene succinate (61%) and polybutylene adipate‐co‐terephthalate (21%). The SOM priming was positively correlated with soil clay and incubation moisture, and negatively correlated with soil organic carbon, total nitrogen, soil C:N ratio, dissolved organic carbon, microbial biomass carbon, carbon input rate, incubation temperature, and soil depth. These results show that the positive PE is ubiquitous in soil ecosystems; its magnitude is linked intrinsically to the physicochemical characteristics and source of exogenous carbon substrate.

## Graphical abstract

Positive priming effect is a common phenomenon in soil ecosystems, with plant residues inducing the highest average priming effect, followed by root exudates and biochar.

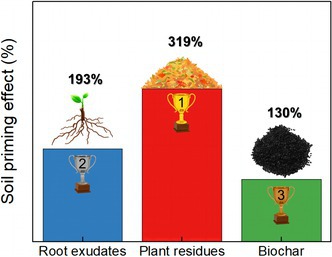

## 1 Introduction

Soil is an important part of the terrestrial ecosystem that stores almost 1500 Pg of organic carbon, which is approximately 2–3 times the size of plant and atmospheric carbon pools (Fu, Xu, et al. 2025; Xu et al. 2025). The stability of the large soil carbon pool is crucial for regulating the concentration of atmospheric carbon dioxide (CO_2_) to mitigate extreme climate events (Feng et al. 2025). Soil organic carbon (SOC) exists in various forms within soil organic matter (SOM) (Schmidt et al. 2011). The loss of SOM elevates atmospheric CO_2_, which exacerbates global warming, whereas sequestering and accumulating atmospheric CO_2_ in SOM mitigates the threat of climate warming (Armstrong McKay et al. 2022). The loss and sequestration of SOM are essentially governed by both plant net primary production and microbial activity involved in the mineralization of SOM (von Luetzow et al. 2006; Wu et al. 2012). Mounting evidence indicates that plant‐derived carbon substrates can either stimulate or suppress SOM mineralization by microbial regulation, a phenomenon known as the priming effects (PE) (Wang et al. 2026; Wu et al. 1993). Soil PE can exert more effects on SOM dynamics than climate factors such as warming and drought (He et al. 2024; Zhu and Cheng 2011). The PE can accelerate SOM mineralization by 380% or reduce it by 50% (Kuzyakov et al. 2000). Therefore, a comprehensive understanding of the mechanisms controlling PE is essential to elucidate soil carbon cycling.

Several theories have been proposed to explain SOM mineralization through PE: co‐metabolism (Huo et al. 2024), preferential substrate utilization (Zhou et al. 2023), microbial community structure transformation (Chen et al. 2022), microbial activation (Drake et al. 2013), stoichiometric decomposition (Feng and Zhu 2021), and microbial nitrogen mining (Zhang et al. 2023). However, soil PE is regulated by many factors and through diverse mechanisms. The input of nutrients (especially nitrogen) is generally considered to limit mining of mineral nitrogen by K‐strategist microorganisms, which reduces SOM priming (Fontaine et al. 2011; Wang et al. 2021). Some research has shown that mineral nitrogen mining is weak because the input of substrates provides a preferable carbon and energy source for microorganisms, reducing SOM breakdown (Mason‐Jones and Kuzyakov 2017; Wild et al. 2017). Conversely, stoichiometric decomposition indicates that substrates with an optimal carbon: nitrogen ratio (C:N) enhance microbial respiration and assimilation (especially *r*‐strategist microorganisms that grow rapidly), resulting in greater SOM mineralization through PE (Mehnaz et al. 2019). Rapid microbial turnover, however, can also increase the accumulation of necromass and its association with metal (oxyhydr)oxides by microbial and mineral carbon pumps, such as the entombing effect, ex vivo modification, and in vivo turnover, thereby reducing the intensity of PE and SOM mineralization (Tao et al. 2023; Wang, Gao, et al. 2024). In nutrient‐poor soil, the input of carbon substrates can stimulate growth and competition between *r‐* and K‐strategist microorganisms, which increases SOM priming through co‐metabolism and transformation of microbial community structure (Tao et al. 2023; Wang and Kuzyakov 2024a). In nutrient‐rich soil, the input of carbon substrates can increase soil acidification by nutrient enrichment (Hu, Delgado‐Baquerizo, et al. 2024). The decrease of soil pH can strengthen metal (oxyhydr)oxide and SOM associations that suppress PE (Wang and Kuzyakov 2024b), whereas other studies indicated that lower pH increases PE intensity because of stimulated *Acidobacteria* growth (Aye et al. 2018; Fan et al. 2019). The prediction of SOM mineralization responses to carbon substrates under diverse soil properties and experimental conditions remains challenging, despite advances in the understanding of soil PE mechanisms.

The physicochemical properties and types of external carbon substrates are important factors in regulating the magnitude and direction of PE (Xu, Wang, et al. 2024). Plant residues induced a greater PE under warming conditions than root exudates (Dong et al. 2024). The input of mineral nitrogen decreased PE from root exudates and microplastics, whereas it increased biochar‐induced PE (Dong et al. 2025). Intensity of the PE of high‐quality plant residues (C:N < 25) was greater than that induced by low‐quality plant residues (C:N > 25), due to the regulation of stoichiometric decomposition (Zhang et al. 2025). Among root exudates, the mean PE was about 39%; monosaccharides induced the greatest PE, followed by organic acids from microbial co‐metabolism (Yan et al. 2023). Mean PE from biochar was about −4%; it was less from crop‐derived biochar than woody‐ and herb‐derived biochar because of preferential use of the biochar substrate by microorganisms (Wang et al. 2016). Degradable plastics (e.g., polylactic acid and polyhydroxyalkanoates) are primarily manufactured from plant‐derived materials such as sugars and vegetable oils (Dong et al. 2025). The PE induced by the decomposition of degradable plastic products, specifically polylactic acid microplastics, polybutylene succinate microplastics, and polyhydroxyalkanoates microplastics ranged from −29% to 43%, 93%–180%, and 550%–1700%, respectively, which was attributed to the mineral nitrogen mining by soil microorganisms (Zhang et al. 2023). These results show that the intensity and direction of PE and microbial effects vary substantially across external carbon substrates (e.g., plant residues, root exudates, biochar, degradable microplastics), and considerable component‐specific variation within substrates (e.g., monosaccharides, polysaccharides, organic acids in root exudates). Although these PE phenomena have been documented in previous studies, their underlying regulatory mechanisms remain poorly elucidated. In particular, how carbon source type (e.g., organic acids and sugars in root exudates) governs the intensity of the PE, and how soil edaphic variables interact with the intrinsic characteristics of these carbon substrates to modulate PE intensity, remain unclear.

To address these gaps in knowledge, we conducted a global meta‐analysis to determine the variation in SOM mineralization from priming induced by the input of four carbon sources: plant residues, root exudates, biochar, and degradable microplastics. The data were derived from 8015 observations in 283 publications to achieve three objectives: (1) to evaluate the effect of four carbon sources on PE; (2) to determine how substrate‐specific characteristics within each carbon source (e.g., non‐woody vs. woody plant residues; sugar vs. organic acids in root exudates) modulate; (3) to explore how soil variables affect SOM mineralization through priming.

## 2 Methods

### 2.1 Data Sources and Inclusion Criteria

**FIGURE 1:**
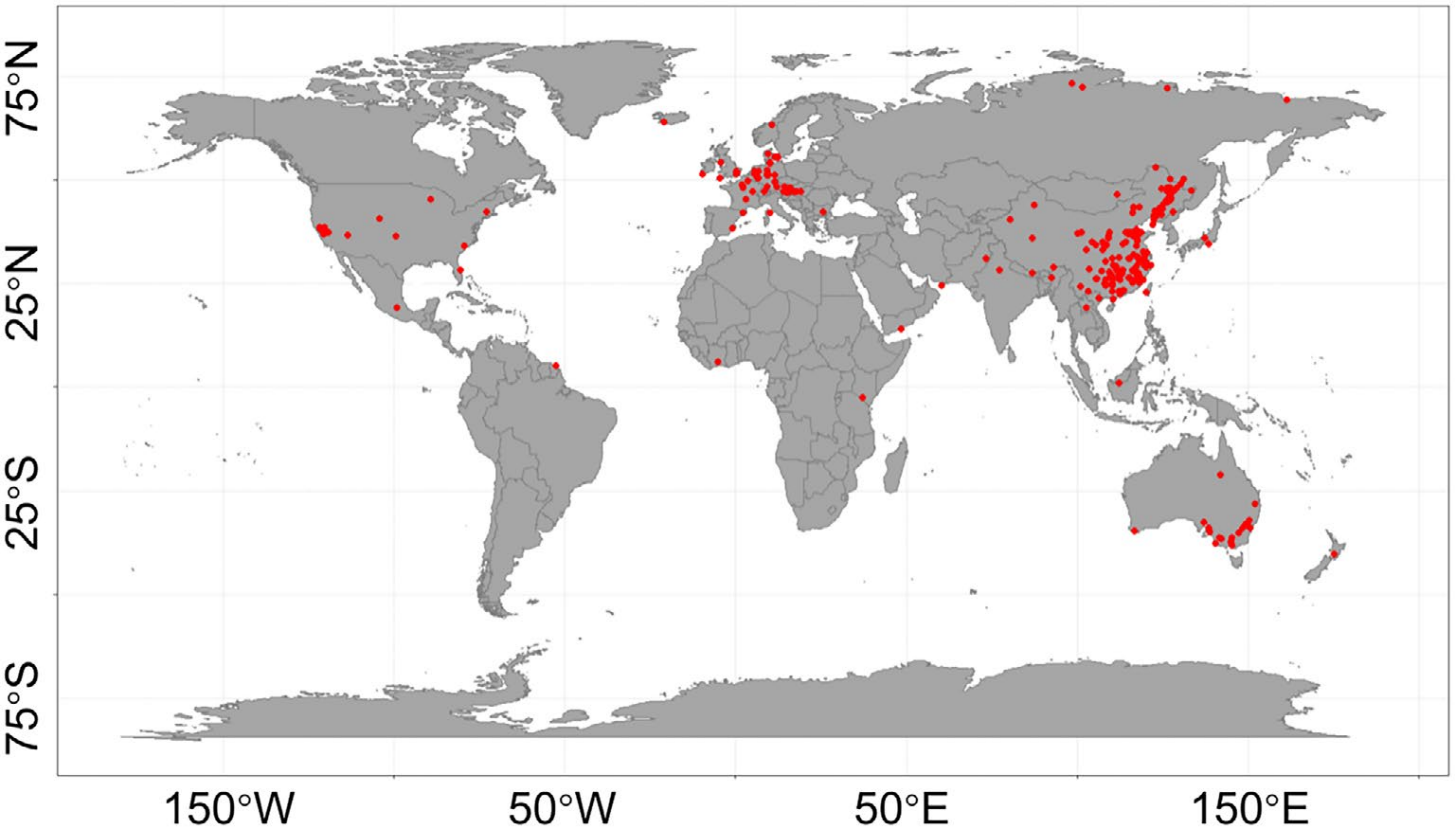
The soil sampling sites used in meta‐analysis.

We searched for relevant papers in the China National Knowledge Infrastructure (https://www.cnki.net), Google Scholar (https://scholar.google.com), and Web of Science (http://apps.webofknowledge.com, until January 7, 2026, Figure S1). Our search included the following terms and a combination of them: “isotope”, “^14^C labeled”, “^13^C labeled”, “priming”, “decomposition”, “respiration”, “CO_2_”, “carbon dioxide”, “terrestrial”, “soil”, and “land”. The studies included in the meta‐analysis should meet the following criteria: (1) soil with the addition of external carbon substrate was set as the treatment group, (2) inclusion of SOM‐derived CO_2_ in the publication, (3) CO_2_ emission from SOM calculated by the ^13^C or ^14^C isotope tracer technique, (4) consideration of soil PE induced by a single external carbon substrate, (5) no nutrient additions in the laboratory incubation experiment. The GetData Graph Digitizer data extraction software (http://www.getdata‐graph‐digitizer.com/index.php) was used to extract the observations from the 283 publications. If the standard deviation of the observation was missing from the publication, we used 1/10 of the mean value to represent it (Dong et al. 2024). Exogenous organic carbon substrates were divided into plant residues, root exudates, biochar, and degradable microplastics (Table S1). The plant residues were divided into non‐woody and woody types (Huo et al. 2017). Root exudates were grouped by their components: monosaccharides, polysaccharides, phenols, and organic acids (Yan et al. 2023). Biochar was grouped by pyrolysis material into non‐woody and woody sources (Wang et al. 2016). Microplastics were grouped by composition into polyhydroxyalkanoates, polybutylene succinate, polylactic acid, and polybutylene adipate‐co‐terephthalate (Zhang et al. 2023). The variables associated with PE treatments were also included: SOC, total nitrogen (TN), soil C:N, soil pH, dissolved organic carbon (DOC), microbial biomass carbon (MBC), soil clay content, mean soil depth, incubation moisture, incubation temperature, incubation time, rate of carbon input, ecosystem types, and climatic zones. We also used the random forest model to calculate the relative importance of variables (Breiman 2001). Soil sampling sites are shown in Figure 1.

### 2.2 Data Calculation and Analysis

The external substrate‐derived CO_2_ (C_e_, mg C kg^−1^ soil), SOM‐derived CO_2_ (C_s_, mg C kg^−1^ soil), and PE (C_PE_, %) were analyzed using the following method (Dong et al. 2024):

(1) $$C_{s}=C_{t}\times \left(\delta_{t}-\delta_{e}\right)/\left(\delta_{\mathrm{ck}}-\delta_{e}\right)$$

(2) $$C_{e}=C_{t}-C_{s}$$

(3) $$C_{\mathrm{PE}}=100\times \left(C_{s}-C_{\mathrm{ck}}\right)/C_{\mathrm{ck}}$$

where *C*_*t*_ (mg C kg^−1^ soil) and *δ*_*t*_ (‰) are the total CO_2_ emissions and ^13^δ from external carbon substrate addition soil, *C*_*ck*_ (mg C kg^−1^ soil) and *δ*_*ck*_ (‰) are the total CO_2_ emissions and ^13^δ of total CO_2_ respired from the soil without external carbon substrate addition, and δ_e_ (‰) is the ^13^δ of the external carbon substrate.

The response ratio (or effect size, RR) is used to estimate the effect of the addition of external carbon substrate on various response variables. The RR was calculated as follows: (Dong et al. 2021; Fu, Liu, et al. 2025):

(4) $$\mathrm{RR}=\text{In}Y_{t}-\text{In}Y_{c}$$

where *Y*_t_ and *Y*_c_ are the mean values of the treatment and control groups, respectively. An RR > 0 represents the addition of external carbon substrate that has a positive effect, whereas RR < 0 indicates a negative effect. We converted RR into a percentage change to facilitate the explanation: Percentage change = 100 × (*e* ^RR^–1), where *e* is a natural constant or Euler number.

The variance (*v*) of RR was calculated as follows (Tao et al. 2026; Zhang et al. 2020):

(5) $$v=\frac{S_{t}^{2}}{N_{t}Y_{t}^{2}}+\frac{S_{c}^{2}}{N_{c}Y_{c}^{2}}$$

where *S*_t_ and *S*_c_ are the standard deviations of the treatment group and the control group, respectively, *N*_t_ and *N*_c_ are the sample sizes of the treatment group and the control group.

The net carbon balance was calculated as the difference between the amount of added carbon (plant residues, root exudates, biochar, and degradable microplastics) remaining in the soil and the cumulative primed carbon from the soil (Chen et al. 2022).

We used the random‐effect models to calculate the size of the mean effect related to the true variation in the sizes of effect between studies. The between‐study variance (*τ* ^2^) was calculated by restricted maximum likelihood because it is more reliable for continuous data. The weighting factor [1/(*v* + *τ* ^2^)] was calculated using *τ* ^2^ and the within‐study variance (*v*). The 95% confidence intervals were determined by the variance‐weighted bootstrapping method (999 iterations). Linear regression analysis was used to explore the relation between the size of the PE and measured soil variables. Various moderators can be used in part to explain heterogeneity in the size of effects with Q statistics. Significant heterogeneity in the sizes of group cumulative effects indicates that part of the total heterogeneity can be explained by the moderator. We tested the publication bias by the methods of Rosenthal's fail‐safe, and trim and fill (Duval and Tweedie 2000; Rosenthal 1979). The relative importance of variables affecting SOM priming was assessed by the random forest model. Model robustness was evaluated by the cross‐validation consistency method, which determined the goodness‐of‐fit with the coefficient of determination (*R* ^2^) through repeated random splitting of the dataset into training and test subsets. All the meta‐analyses in the research were performed with R (version 4.4.2) statistical software by the *metafor* and *randomForest* packages (Breiman 2001; Viechtbauer 2010).

## 3 Results

### 3.1 Effects of Four Carbon Sources on the Priming Effects

**FIGURE 2:**
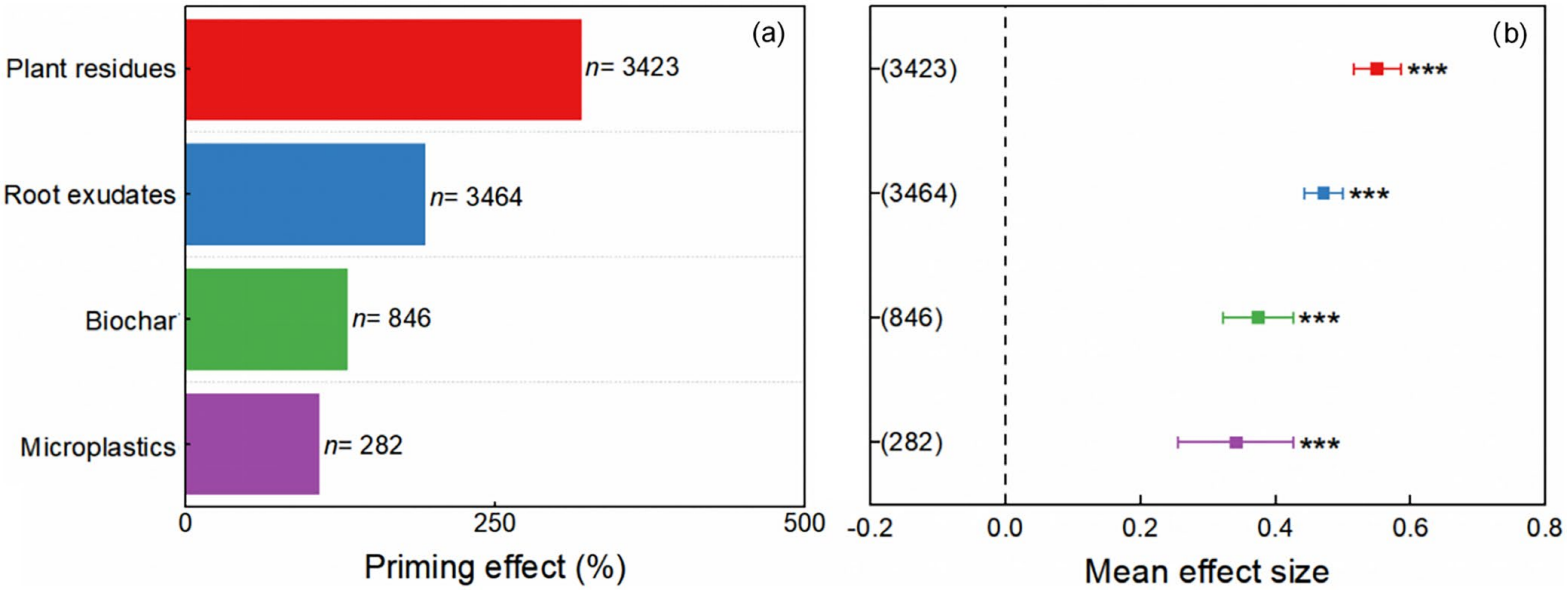
Size of mean values (a) and mean effect (b) of four carbon sources on soil organic matter mineralization by the priming effect (*p* < 0.001). Error bars are 95% bootstrapped confidence intervals.

**FIGURE 3:**
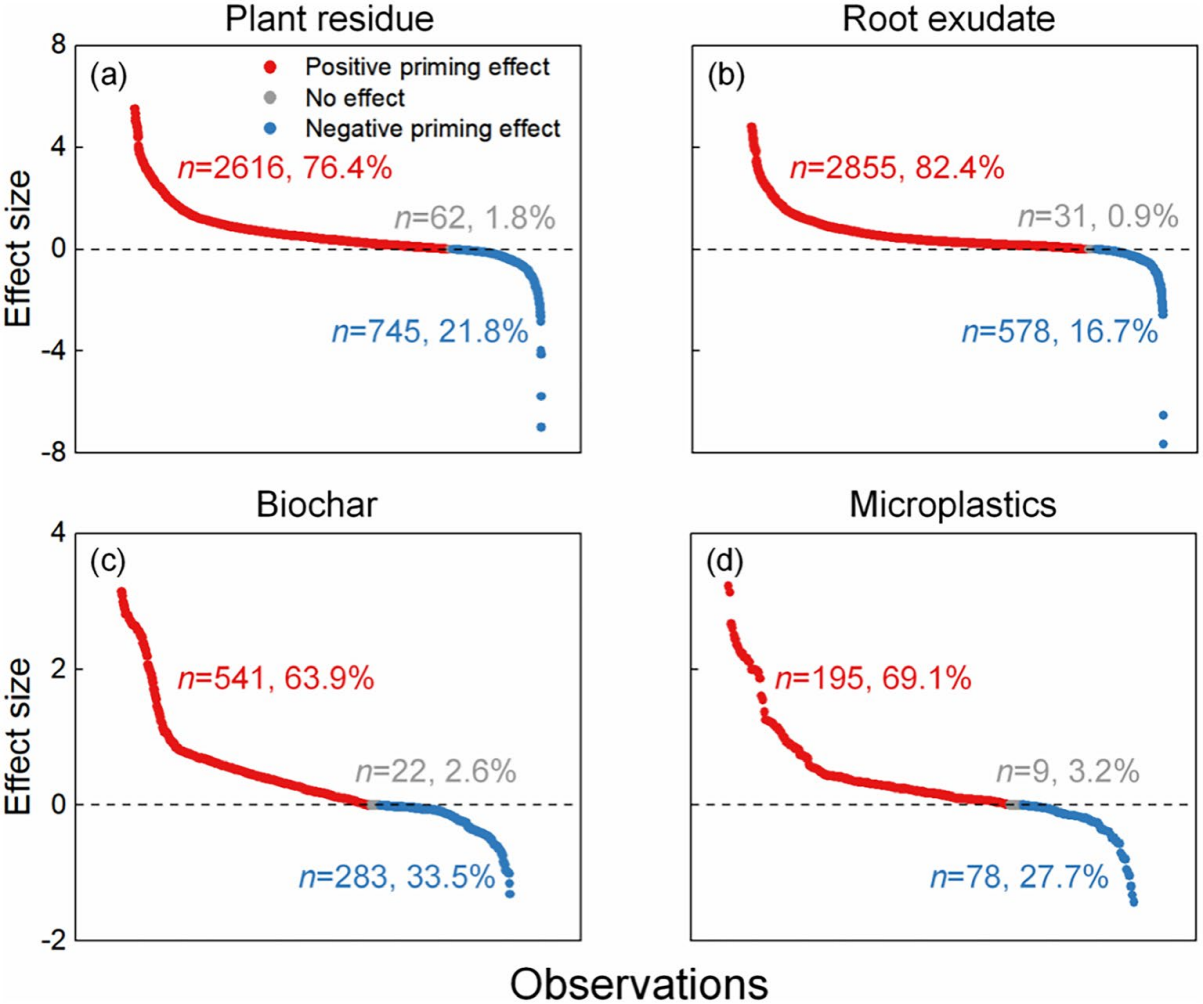
Graphs of percentage values plotted against for positive and negative effects of four carbon sources (plant residues, root exudates, biochar, and microplastics) on soil organic matter mineralization by the priming effect. A positive effect size suggests that four carbon sources increase soil organic matter mineralization by the priming effect, whereas a negative effect size suggests that four carbon sources decrease soil organic matter mineralization by the priming effect. The *n* is sample size, and the number after the comma shows the percentage of positive and negative effects of four carbon sources on soil organic matter mineralization by the priming effect.

The results indicated that in general the input of four carbon substrates increased mineralization of SOM (i.e., positive PE, Figure 2). The average PE induced by plant residues, root exudates, biochar, and microplastics were 319%, 193%, 130%, and 107%, respectively (Figure 2a). Compared to the soil without the input of external carbon substrate, that with the input of plant residues, root exudates, biochar, and microplastics increased SOM mineralization by 73%, 60%, 45%, and 41%, respectively (Figure 2b). Specifically, in the data of PE induced by plant residues, 76.4% of effect sizes were higher than zero (i.e., positive PE), and 21.8% of effect sizes were lower than zero (i.e., negative PE, Figure 3a). In the data of PE induced by root exudates, 82.4% of effect sizes were higher than zero, and 16.7% of effect sizes were lower than zero (Figure 3b). In the data of biochar induced PE by, 63.9% of effect sizes were higher than zero, and 33.5% of effect sizes were lower than zero (Figure 3c). In the data of degradable microplastics induced PE, 69.1% of effect sizes were higher than zero, and 27.7% of effect sizes were lower than zero (Figure 3d).

**FIGURE 4:**
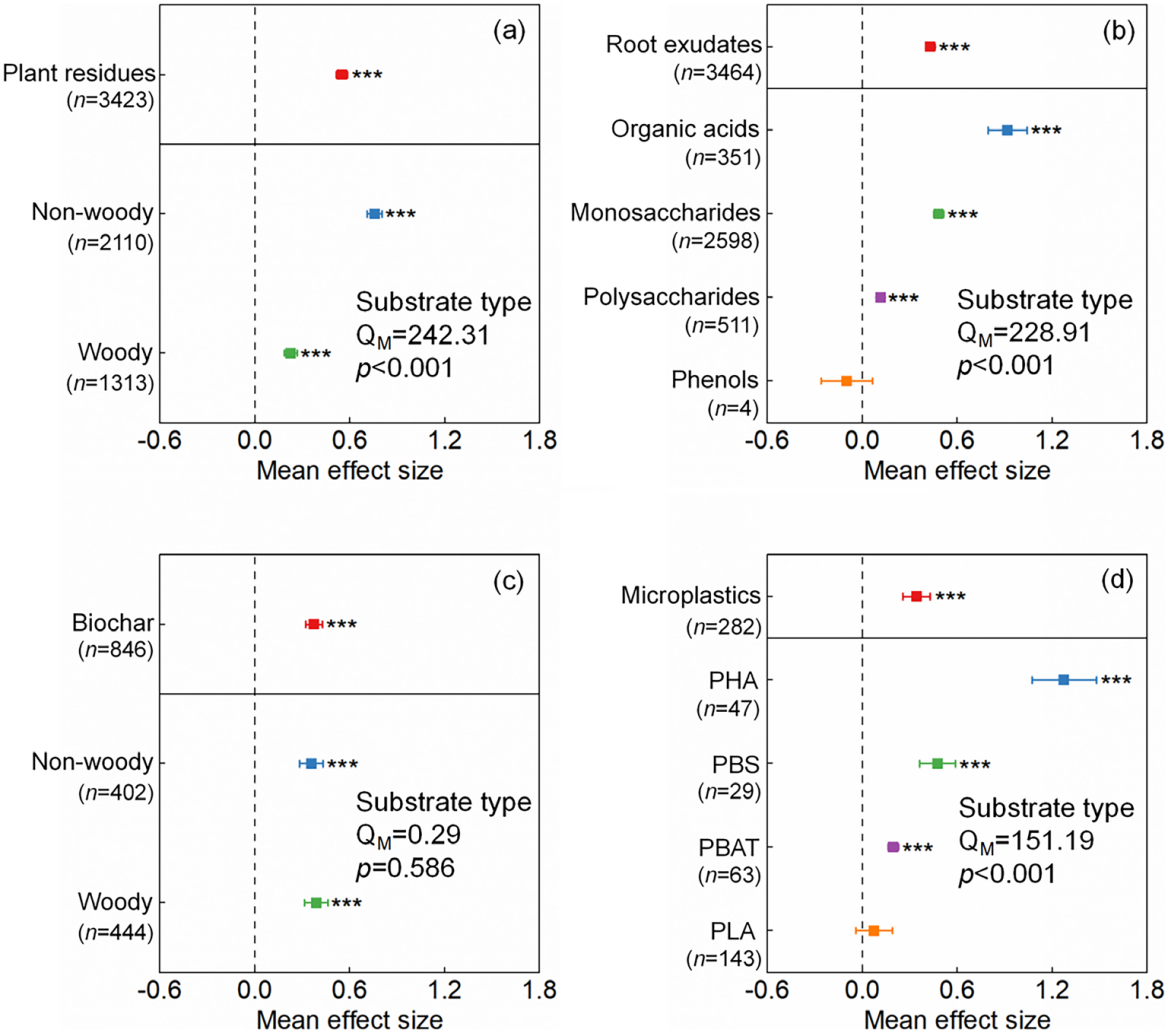
Size of mean effect of plant residues (a), root exudates (b), biochar (c), and microplastics (d) on soil organic matter mineralization by the priming effect (*p* < 0.001). Error bars are 95% bootstrapped confidence intervals. PBAT, polybutylene adipate‐co‐terephthalate; PBS, polybutylene succinate; PHA, polyhydroxyalkanoates; PLA, polylactic acid; Q_M_, heterogeneity in group cumulative effect sizes.

**FIGURE 5:**
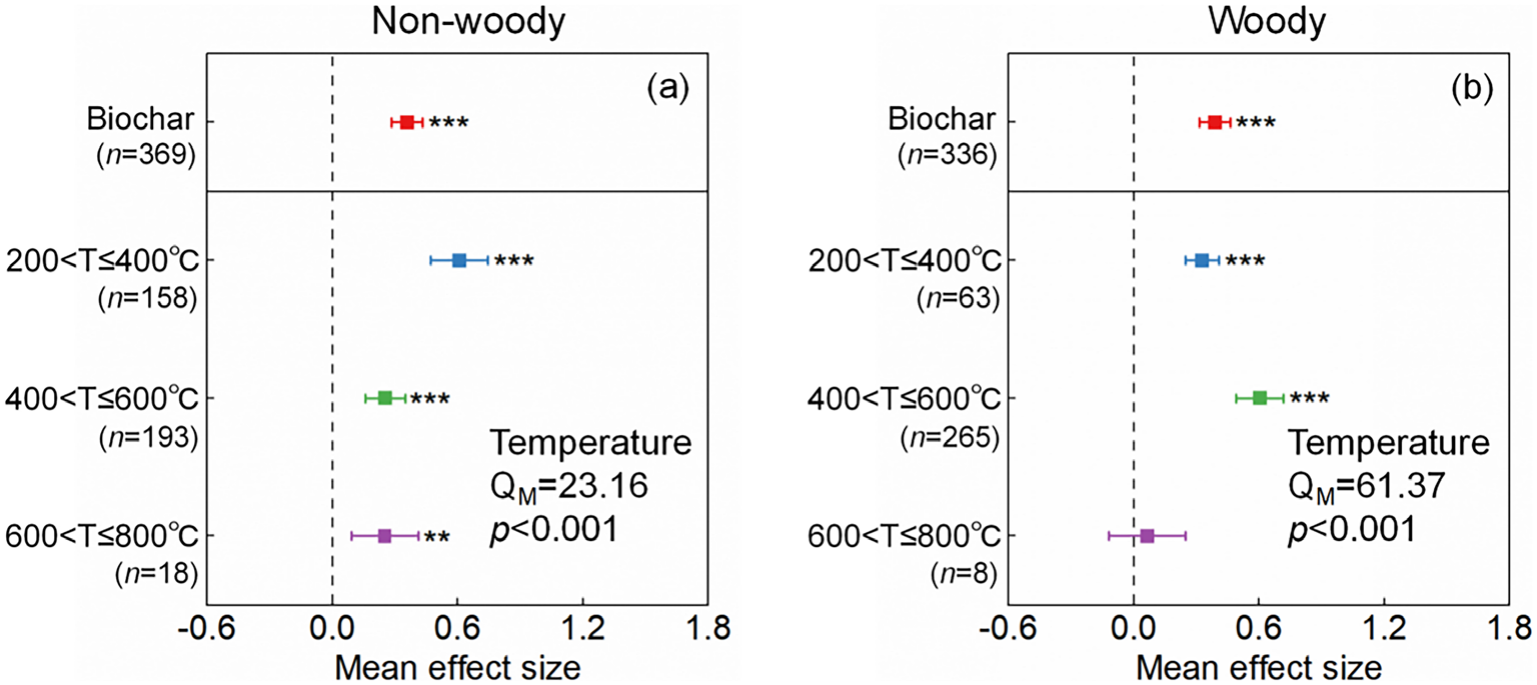
Mean effect size of non‐woody and woody biochars pyrolysis temperatures (T) on soil organic matter mineralization by priming effect. Q_M_, heterogeneity in group cumulative effect sizes.

Compared to soil without carbon substrate inputs, additions of non‐woody and woody residues led to increases in SOM mineralization of 113% and 25%, respectively (Figure 4a). Among the components of root exudates, organic acids induced the greatest mineralization of SOM (151%), followed by monosaccharides (53%) and polysaccharides (12%, Figure 4b). The SOM mineralization induced by woody biochar increased by 48%, which was more than that of non‐woody biochar (43%, Figure 4c). The average pyrolysis temperature of wood biochar was higher than that of non‐woody biochar (Figure S2). For non‐woody biochar, SOM mineralization induced by materials pyrolyzed at 200°C–400°C, 400°C–600°C, and 600°C–800°C increased by 84%, 29%, and 29%, respectively (Figure 5a). For woody biochar, SOM mineralization increased by 38% and 83% at pyrolysis temperatures of 200°C–400°C and 400°C–600°C, respectively (Figure 5b). The addition of polyhydroxyalkanoates (258%) microplastics stimulated SOM mineralization more than polybutylene succinate (61%) and polybutylene adipate‐co‐terephthalate (21%, Figure 4d).

### 3.2 Effects of Ecosystem and Climate Zone on Priming Effects

Across different ecosystem types, the input of plant residues increased SOM mineralization via the PE by 102%, 135%, 32%, and 49% in cropland, grassland, forest, and wetland soils, respectively (Figure S3a). Root exudate input enhanced SOM mineralization by 71%, 35%, 62%, and 16% in cropland, grassland, forest, and wetland soils, respectively (Figure S3b). Biochar induced the strongest SOM mineralization in grassland soil (171%), followed by cropland (43%), wetland (22%), and forest soil (9%; Figure S3c). The addition of degradable microplastics stimulated SOM mineralization via the PE by 41% and 27% in cropland and forest soils, respectively (Figure S3d).

Across different climatic zones, plant residues input increased SOM mineralization via the PE by 34%, 75%, and 5% in tropical, temperate, and boreal zones, respectively (Figure S4a). Root exudate input enhanced SOM mineralization by 15%, 65%, and 57% in tropical, temperate, and boreal zones, respectively (Figure S4b). Biochar input increased SOM mineralization by 148% and 29% in tropical and temperate zones, respectively (Figure S4c). The addition of degradable microplastics stimulated SOM mineralization via the PE by 308% and 38% in tropical and temperate zones, respectively (Figure S4d).

### 3.3 Correlations Between SOM Priming, Soil Physicochemical Properties, and Experimental Variables

**FIGURE 6:**
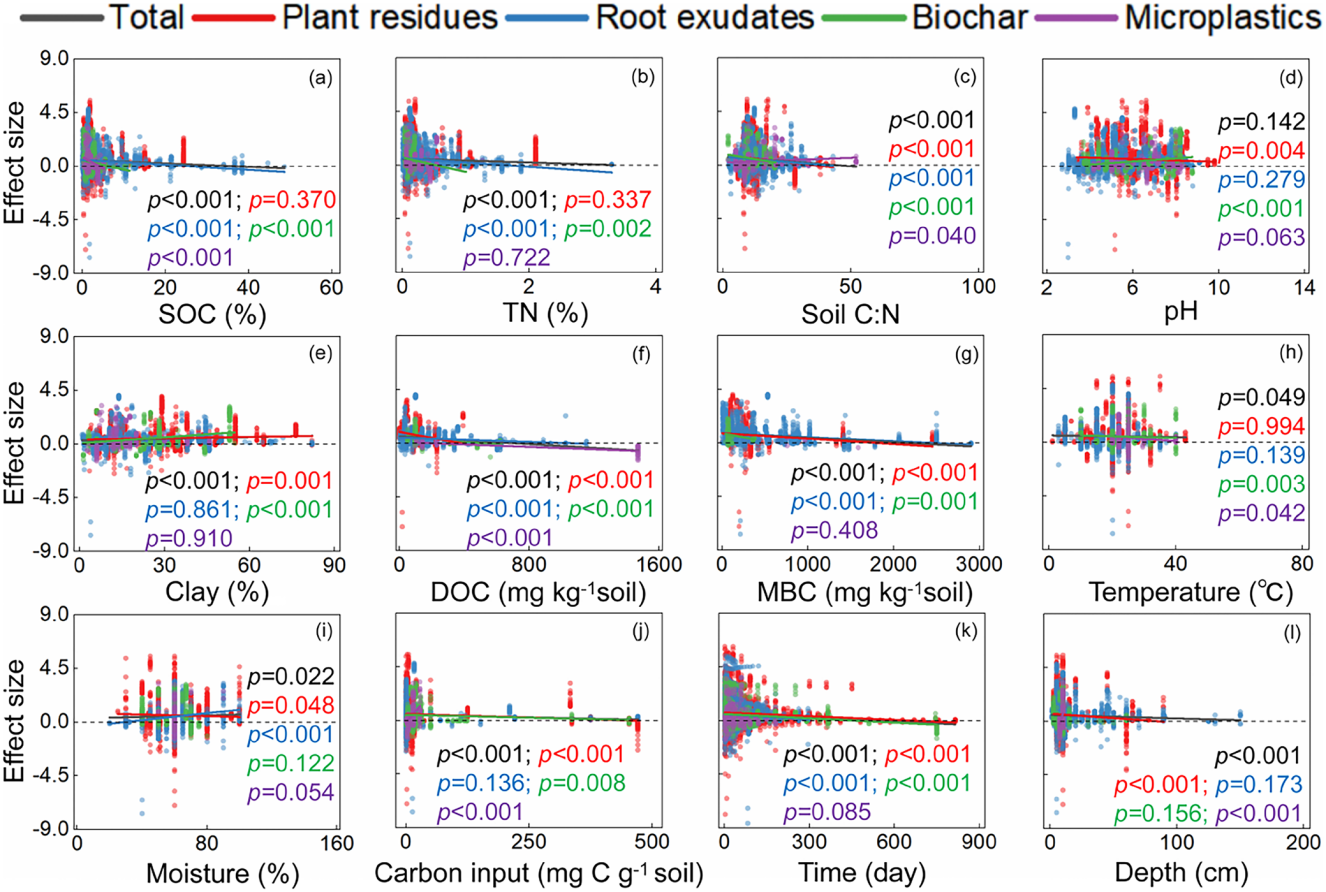
Linear relations between size of the effect of soil organic matter priming with the soil variables. The black, red, blue, green, and purple lines represent the total soil organic matter priming induced by plant residues, root exudates, biochar, and biodegradable microplastics, respectively. Carbon input, rate of external carbon input; clay, soil clay content; depth, mean soil depth; DOC, dissolved organic carbon; MBC, microbial biomass carbon; moisture, incubation moisture; SOC, soil organic carbon; Soil C:N, SOC to TN ratio; temperature, incubation temperature; time, incubation time; TN, total nitrogen.

**FIGURE 7:**
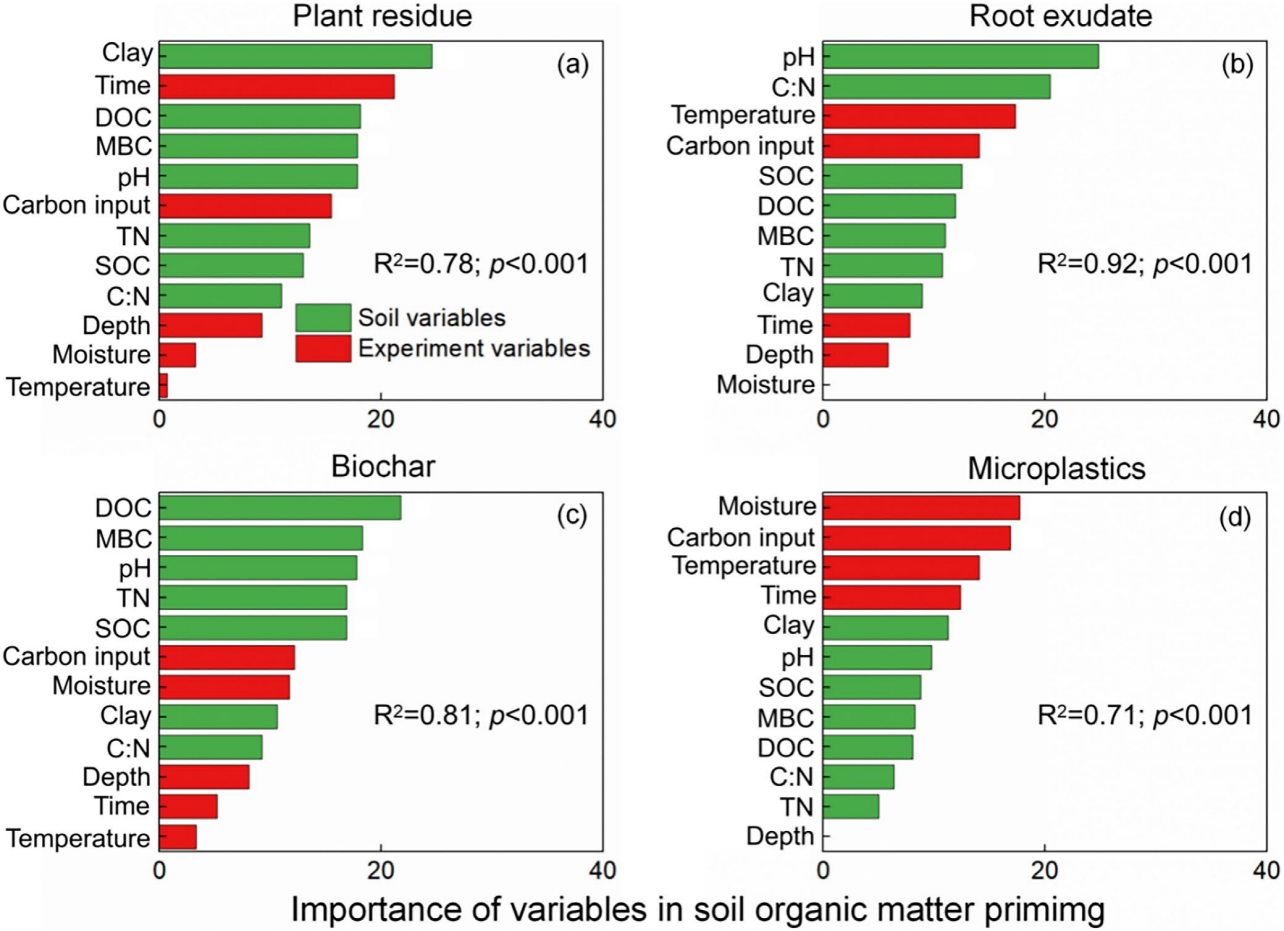
Relative importance of variables affecting soil organic matter priming induced by plant residues, root exudates, biochar, and microplastics determined by a random forest model. C:N, SOC to TN ratio; carbon input, external organic carbon input rate; clay, soil clay content; depth, mean soil depth; DOC, dissolved organic carbon; MBC, microbial biomass carbon; Moisture, incubation moisture; SOC, soil organic carbon; temperature, incubation temperature; time, incubation time; TN, total nitrogen.

The input effects of four carbon substrates (plant residues, root exudates, biochar, and microplastics) on SOM priming are positively correlated with soil clay and incubation moisture, and negatively correlated with SOC, TN, soil C:N, DOC, MBC, incubation temperature, rate of carbon input, mean soil depth, and length of incubation time (Figure 6). The SOM priming induced by plant residues is positively correlated with soil clay content, and negatively correlated with soil C:N, pH, DOC, MBC, incubation moisture, incubation time, rate of carbon input, and mean soil depth (Figure 6). Priming of SOM induced by root exudates was positively correlated with incubation moisture, and negatively correlated with SOC, TN, soil C:N, DOC, MBC, incubation time, rate of carbon input, and mean soil depth (Figure 6). The SOM priming induced by biochar is positively correlated with soil pH and clay content, and negatively correlated with SOC, TN, DOC, MBC, incubation temperature, incubation time, rate of carbon input, and mean soil depth (Figure 6). Priming of SOM induced by microplastics is positively correlated with soil C:N, whereas negatively correlated with SOC, DOC, incubation temperature, carbon input rate, and mean soil depth (Figure 6). The random forest model indicated that soil clay content was the most crucial factor in mediating priming induced by the plant residues (Figure 7a). The soil pH was the most crucial factor mediating the root exudates‐induced SOM priming (Figure 7b). The DOC was the most crucial factor mediating the biochar‐induced SOM priming (Figure 7c). The incubation moisture was the most crucial factor mediating the microplastics‐induced SOM priming (Figure 7d).

### 3.4 Effects of Four Carbon Sources on Net Carbon Balance

The average net carbon balance in soils following the input of plant residues, root exudates, biochar, and microplastics was 2.83, 2.26, 27.25, and 8.08 mg C g^−1^ soil, respectively (Figure S5). Specifically, for plant residue input, 86.8% of effect sizes for net carbon balance were higher than zero (i.e., substrate addition increases soil carbon content), with only 13.2% lower than zero (i.e., substrate addition decreases soil carbon content; Figure S6a). For root exudate input, 76.0% of the effect sizes were higher than zero, and 24.0% were lower than zero (Figure S6b). For biochar input, 96.8% of the effect sizes were higher than zero, with a mere 3.2% lower than zero (Figure S6c). For degradable microplastic input, 100% of the effect sizes for net carbon balance were higher than zero (Figure S6d).

### 3.5 Publication Bias

Rosenthal's fail‐safe numbers for the effects of plant residues, root exudates, biochar, and microplastics on SOM priming are 212,459,258, 192,566,628, 6,406,764, 512,448, respectively, suggesting that the results are robust (Table S2). The mean effect sizes from the original and trim‐and‐fill model for SOM priming induced by plant residues were 0.55 (95% confidence intervals from 0.52 to 0.58) and 0.86 (95% confidence intervals from 0.82 to 0.89), respectively (Table S2). The mean effect sizes from the original and trim‐and‐fill model for SOM priming induced by root exudates were 0.47 (95% confidence intervals from 0.44 to 0.50) and 0.71 (95% confidence intervals from 0.68 to 0.74), respectively (Table S2). The mean effect sizes from the original and trim‐and‐fill model for SOM priming induced by biochar were 0.37 (95% confidence intervals from 0.32 to 0.43) and 0.37 (95% confidence intervals from 0.32 to 0.43), respectively (Table S2). The mean effect sizes from the original and trim‐and‐fill model for SOM priming induced by microplastics were 0.34 (95% confidence intervals from 0.26 to 0.43) and 0.34 (95% confidence intervals from 0.26 to 0.43), respectively (Table S2). The original and trim‐and‐fill models indicated that missing values in the data for SOM priming did not affect the results.

## 4 Discussion

### 4.1 Plant Residues‐Induced Priming Effects

Plant residues are an important carbon substrate in regulating soil microbial metabolic processes through respiratory decomposition and biomass assimilation (Poeplau et al. 2024; Wu et al. 2019). The results of the meta‐analysis showed that the input of non‐woody and woody residues enhanced SOM mineralization by 113% and 25%, respectively, through the PE (Figure 4a). Cellulose, hemicellulose, and lignin are important components of plant residues (Chaturvedi and Verma 2013; Deng et al. 2016). Compared to plant residues from woody, the greater SOM priming from non‐woody residues (like herbs and crops) than from woody residues is because of their greater proportions of cellulose and hemicellulose (He et al. 2023; Reddy and Yang 2009). Unlike lignin, the low thermal stability of cellulose and hemicellulose is conducive to their rapid decomposition, which stimulates microbial growth (Ornaghi Jr. et al. 2023; Parajuli et al. 2022). This process enhances the PE through microbial activation and co‐metabolism processes (Fu, Liu, et al. 2025).

Woody residues contain greater lignin contents because of the developed nature of the roots and hard stems of perennial woody plants and increased secondary xylem (Han et al. 2022). Compared with cellulose and hemicellulose, lignin decomposition requires greater activation energy; therefore, it degrades slowly in soil (He et al. 2024; Yeo et al. 2019). Lignin‐like compounds interact more strongly with clay minerals in soil because of their slow degradation and persist for longer periods in soil (Chen et al. 2017; Islam et al. 2025; Liu et al. 2025). The random forest model also indicated that clay content was the most crucial factor in mediating SOM priming from plant residues (Figure 7a). The larger content lignin‐like compounds in woody plants directly decreases the active organic carbon from residue decomposition, thereby decreasing the carbon and energy sources for microbial growth, which limits SOM priming (Coward et al. 2019; Santos et al. 2021). The combination of lignin‐like compounds in woody residues with clay minerals in soil was also conducive to the formation and sequestration of SOM, which improved soil aggregate stability by interactions between lignin and clay minerals (Hu, Xu, et al. 2024), adsorption of active organic carbon in the soil solution onto the surface and pores of woody residues and clay minerals (Kou et al. 2025), increases in fungal necromass accumulation and sequestration such as the entombing effect (Jin et al. 2024; Xiao et al. 2024), thereby indirectly decreasing SOM priming (Figure 4a).

Overall, the present study highlights the critical role of plant residue quality in modulating the PE. Specifically, the contrasting PE of non‐woody and woody residues are governed by their distinct chemical compositions, with cellulose‐ and hemicellulose‐rich non‐woody residues stimulating microbial co‐metabolism, thereby enhancing the PE; in contrast, woody residues with high lignin content suppress the PE through slow decomposition, strong associations with soil minerals, and promotion of soil aggregate stability and microbial necromass sequestration. This finding emphasizes that plant residue quality (i.e., cellulose vs. lignin content) is a key determinant of PE intensity, which is of great significance for predicting SOM dynamics under different land management practices.

### 4.2 Root Exudates‐Induced Priming Effects

**FIGURE 8:**
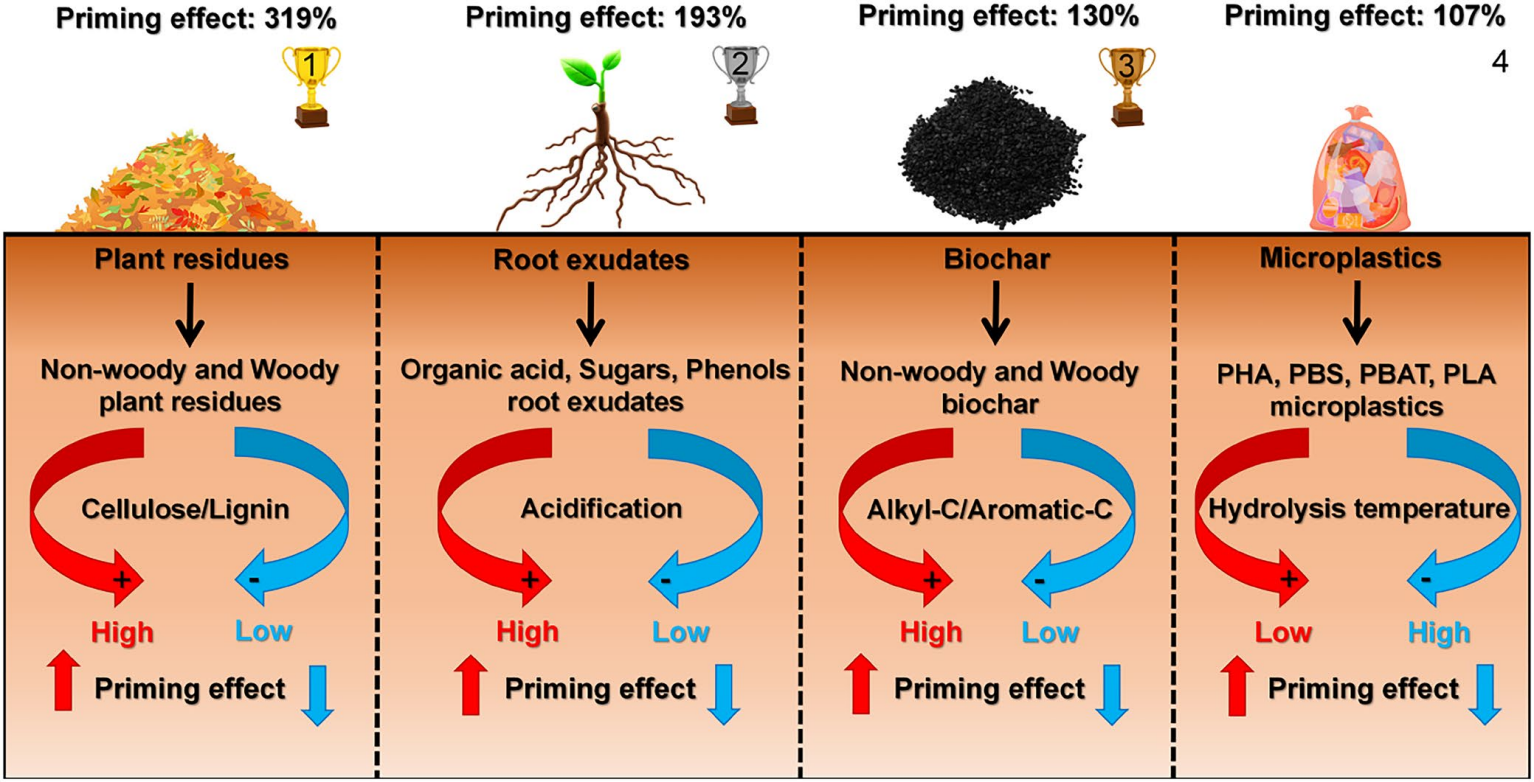
The impact of plant residues, root exudates, biochar, and microplastics input on the soil priming effect. ATP, adenosine triphosphate; PBAT, polybutylene adipate‐co‐terephthalate; PBS, polybutylene succinate; PHA, polyhydroxyalkanoates; PLA, polylactic acid.

Root exudates from plants are composed of monosaccharides, polysaccharides, organic acids, phenolics, and other compounds (Yan et al. 2023). We found that the SOM priming induced by organic acids was greatest, followed by monosaccharides, polysaccharides, and phenolics (Figure 4b). First, the strong SOM priming induced by organic acids can be partially explained by the regulation of microbial stoichiometric decomposition (Feng and Zhu 2021). On the one hand, some organic acids (e.g., amino acids) have a larger microbial utilization efficiency and contain elements of nitrogen (Yan et al. 2023). On the other hand, organic acids in the soil can dissolve minerals by chelation and acidification to destroy soil metal‐bound organic carbon (Wen et al. 2025), which makes recalcitrant carbon and nutrients (particularly mineral nitrogen) available for microorganisms (Giehl and von Wirén 2014). The random forest model also indicated that soil pH was the most crucial factor in mediating root exudates‐induced priming of SOM (Figure 7b). Therefore, unlike monosaccharides and polysaccharides, the addition of organic acids can meet soil microbial demand for carbon and nitrogen, and the respiration and assimilation by microorganisms are optimized, resulting in the largest PE (Yan et al. 2023). Second, microbial decomposition of sugars can provide more energy. For example, sugars (e.g., glucose and fructose) produce about 28 to 32 ATP during aerobic respiration, whereas organic acids (e.g., acetate) produce about 12 ATP (Pastor et al. 2019; Wiesenbauer et al. 2025). However, soil microorganisms can metabolize organic acids (e.g., acetate) to form acetyl‐coenzyme A, thereby bypassing the glucose decarboxylation to acetyl‐coenzyme A step and entering the tricarboxylic acid cycle (Wiesenbauer et al. 2025; Zhang et al. 2019). The acetic acid to acetyl‐coenzyme A step is a shorter metabolic pathway, resulting in faster rates of adenosine triphosphate production (Kreft and Bonhoeffer 2005; Kreft et al. 2020). Therefore, the input of organic acids is beneficial to increasing SOM mineralization through the PE (Figure 8).

Soil microbial communities are evolutionarily adapted to metabolize simple, low‐molecular‐weight compounds (e.g., organic acids and monosaccharides) rather than large, polymeric organic molecules, which is a key principle governing SOM decomposition (Lehmann and Kleber 2015). Specifically, molecules with a molecular weight below 600–700 Da can passively diffuse through microbial cell membrane porins and be directly taken up by microbial cells, whereas larger molecules require extracellular hydrolysis prior to microbial assimilation (Li et al. 2023; Nakae 1976). Monosaccharides (e.g., glucose, ~180 Da) and organic acids (e.g., acetate, ~60 Da) fall well below this molecular threshold, rendering them directly accessible for microbial uptake (Grimm et al. 2003). In contrast, polysaccharides are polymeric molecules composed of numerous sugar monomers, with molecular weights often exceeding 1 MDa, and thus must first be cleaved by extracellular hydrolases into smaller monomeric units before the resulting products become available for microbial uptake (Yeul and Rayalu 2013). Compared with polysaccharides, organic acids and monosaccharides have significantly lower molecular weights, which greatly enhances their microbial accessibility (Lehmann and Kleber 2015). This, in turn, provides soil microorganisms with a readily available carbon and energy source to fuel their metabolic activity, thereby inducing a higher intensity of the PE (Figure 4b). Finally, phenolic compounds, which contain aromatic rings, are more resistant to microbial decomposition and yield less energy per unit carbon, thus limiting the SOM priming (Frey et al. 2013; Yan et al. 2023). Some phenolics are toxic and can also reduce SOM priming by killing soil microorganisms (Girish and Murty 2012).

Collectively, organic acids, sugars, and phenolics are the major compound classes of plant root exudates (Yan et al. 2023). Our results demonstrate that among these root exudate components, organic acids elicit the strongest PE, which is significantly higher than that induced by sugars and phenolics. This suggests that organic acids serve as a particularly vital energy source for microorganisms, driving rapid carbon turnover in the rhizosphere. Consequently, future investigations into root exudate‐induced PE and rhizosphere PE should prioritize the composition of root exudates, with a specific focus on the mechanisms and dynamic processes of organic‐acid‐mediated microbial metabolism.

### 4.3 Biochar‐Induced Priming Effects

Biochar is a carbon‐rich porous material produced by pyrolysis, and that remains stable in soil for decades (Mikajlo et al. 2024). The mean PE (130%) induced by biochar in our meta‐analysis was markedly higher than the value (−4%) that was reported in a previous meta‐analysis (Wang et al. 2016). The discrepancy in observed PE values can be primarily attributed to substantial differences in the study databases and inclusion criteria between the two meta‐analyses. For example, Wang et al. (2016) only included 128 observations from 24 studies, focusing exclusively on biochar produced by dry pyrolysis of organic materials and excluding all incubation studies with temperatures exceeding 40°C. In contrast, the present meta‐analysis incorporated a much larger and more comprehensive dataset of biochar‐induced PE, including 847 observations from 36 studies (Table S1), and placed a specific emphasis on the comparison between woody and non‐woody biochar (e.g., herb and crop‐derived biochar). Our results showed that woody and non‐woody biochar increased the mineralization of SOM by 48% and 43%, respectively (Figure 4c). In addition, the DOC released from biochar decomposition is a key factor regulating the intensity of biochar‐induced PE (Figure 7c), and the quantity and quality of biochar‐derived DOC are closely related to the type of biochar and pyrolysis temperature (Wang et al. 2016), which further contributes to the PE differences between the two studies.

Non‐woody residues, rich in cellulose and hemicellulose, yield biochar with lower aromatic carbon and higher nonaromatic carbon (like alkyl and O‐alkyl carbon) contents (Preston and Schmidt 2006). Rapid decomposition of non‐woody biochar mainly releases DOC containing rich alkyl and O‐alkyl carbon, which can be utilized by soil microorganisms (especially *r*‐strategists) for respiration and assimilation, thereby simultaneously increasing the decomposition of non‐woody biochar and SOM through co‐metabolic processes (Huo et al. 2024; Wang and Kuzyakov 2024a). Increasing the pyrolysis temperature of non‐woody biochar production significantly decreased PE intensity (Figure 5a). For example, the PE intensity induced by biochar prepared at 200°C–400°C was higher than that of biochar pyrolyzed above 400°C (Figure 5a). During the pyrolysis process, the low thermal stability of cellulose and hemicellulose in non‐woody residues leads to their rapid decomposition to CO_2_ (Ornaghi Jr. et al. 2023; Parajuli et al. 2022). As pyrolysis temperature increases, the proportion of aromatic carbon and the degree of aromatic condensation in biochar derived from non‐woody residues both increase, resulting in a lower decomposition rate of biochar and a lower PE (Singh et al. 2012).

Biochar made from lignin‐rich wood has larger aromatic carbon content (Hilscher et al. 2009; Singh and Cowie 2014). Compared with aromatic carbon‐rich DOC from woody biochar decomposition, soil microorganisms (especially K‐strategists) are more likely to utilize SOM for respiration and assimilation (Zhou et al. 2023), thereby increasing PE intensity through preferential substrate utilization by microorganisms (Blagodatskaya and Kuzyakov 2008). The increase in SOM mineralization induced by woody biochar was greatest at pyrolysis temperatures of 400°C–600°C (Figure 5b). Compared with cellulose and hemicellulose, lignin decomposition requires greater activation energy (He et al. 2024; Yeo et al. 2019). Low‐temperature pyrolysis (200°C–400°C) results in woody biochar that retains more non‐pyrolyzed organic residues and nutrients, such as low‐molecular‐weight volatiles (Murtaza et al. 2024; Zhu et al. 2017). This enhances microbial utilization of these readily available substrates, thereby reducing SOM mineralization (Murtaza et al. 2024; Zhou et al. 2023). In contrast, woody biochar produced at 600°C–800°C has a higher aromatic carbon content, which fails to supply sufficient carbon and energy for microbial growth and metabolism, thereby constraining PE intensity and subsequent SOM mineralization (Wang et al. 2016).

Overall, the PE in biochar‐amended soils is jointly regulated by microbial co‐metabolism and preferential substrate utilization. In industrial and agricultural production, appropriate pyrolysis temperatures should be selected based on the type of biochar feedstock (i.e., non‐woody vs. woody) to mitigate soil CO_2_ emissions induced by the PE, while preserving the soil improvement functions of biochar (e.g., fertility promotion and structure improvement).

### 4.4 Degradable Microplastics‐Induced Priming Effects

Degradable microplastics in soil are a hidden carbon substrate that can increase or decrease SOM mineralization through PE (Huang and Xia 2024; Zhao et al. 2024). The input of polyhydroxyalkanoate microplastics stimulated SOM priming more than those from polybutylene succinate, polybutylene adipate‐co‐terephthalate, and polylactic acid (Figure 4d), which results from differences in the degradability of plastics (Figure 8). The biodegradability of microplastics is related to their hydrolysis and melting temperature (Huo et al. 2024; Zhang et al. 2023). The random forest model also indicated that soil moisture was the most crucial factor in mediating microplastics‐induced priming of SOM (Figure 6d). The hydrolysis temperatures are 20°C–60°C for polyhydroxyalkanoates, 60°C for polybutylene succinate, 70°C for polybutylene adipate‐co‐terephthalate, and 70°C–160°C for polylactic acid (Grundmann et al. 2013; Wang, Liu, et al. 2024; Xu, Delgado‐Baquerizo, et al. 2024). The melting temperatures are 45°C–54°C for polyhydroxyalkanoates, 150°C for polybutylene succinate, 160°C–170°C for polybutylene adipate‐co‐terephthalate, and 182°C–200°C for polylactic acid (Ryu et al. 2025; Zhang et al. 2023). Therefore, polyhydroxyalkanoates are easier to decompose than other microplastics under natural conditions.

In field soil, the rate of degradation of polyhydroxyalkanoates was 2.5 times greater than that of polylactic acid (Weng et al. 2013). The electron microscope image showed that in soil, polyhydroxyalkanoates were very degraded at room temperature (25°C), whereas polylactic acid did not change markedly after 38 days of incubation (Jia et al. 2024). Compared to soil without degradable microplastics, soil with polyhydroxyalkanoates can rapidly increase the active organic carbon from microplastic decomposition, thereby increasing microbial growth and SOM priming through microbial activation and co‐metabolism (Drake et al. 2013; Huo et al. 2024). Unlike root exudates and plant residues, most degradable microplastics contain almost no nitrogen (Huang and Xia 2024). Therefore, the rapid degradation of microplastics exacerbates soil C:N imbalance (Shi et al. 2025). This increases nitrogen mining by K‐strategist microorganisms through the synthesis of nutrient‐acquiring enzymes and thus SOM mineralization (Chen et al. 2014; Zhang et al. 2023). At the same time, fast‐growing K‐strategist microorganisms can feed on microbial necromass (Huo et al. 2024), which limits the accumulation of necromass and its contribution to the physical protection of SOM by microbial and mineral carbon pump processes (Xiao et al. 2023; Zhu et al. 2020). The larger soil C:N ratio increased SOM priming from microplastics, which supported the present results (Figure 6c). In contrast, large specific surface area and slow decomposition of microplastics (such as polylactic acid) reduce the active organic carbon in the soil, thus limiting the microbial growth and SOM mineralization (Zhang et al. 2023). The combination of slowly degradable microplastics and SOM also increases the stability of soil aggregates and the adsorption of active organic carbon through plastisphere formation (Chen et al. 2025; Li et al. 2025; Tanunchai et al. 2025), which further decreases SOM mineralization. In addition, the slow decomposition of microplastics in soil can destroy the tertiary enzyme structure and cell membrane, resulting in the death of microorganisms, especially K‐strategists that synthesize nutrient‐acquiring enzymes (Lin et al. 2025), thus limiting mineral nitrogen mining and SOM mineralization.

Overall, our findings highlight the importance of microplastic degradability (especially hydrolysis and melting temperatures) in regulating SOM priming. Rapidly degrading microplastics can accelerate SOM mineralization via the PE, thereby reducing the carbon sequestration potential of soils. In contrast, slowly degrading microplastics may enhance SOM stability through plastisphere‐microorganism‐soil particle interactions. Therefore, in agricultural practices such as plastic film mulching and greenhouse cultivation, it is essential to select microplastic materials with a weak PE (e.g., polylactic acid) instead of high‐risk polymers (e.g., polyhydroxyalkanoates) to minimize soil carbon loss and mitigate the negative impacts on soil health.

### 4.5 Future Perspectives and Limitations

This meta‐analysis provides a comprehensive global synthesis of the PE induced by four major carbon substrates on SOM mineralization. However, several limitations of this study should be acknowledged when interpreting the findings, which also reflect the critical knowledge gaps in the current research field. Despite our extensive and systematic literature search, the geographical distribution of the included study sites is highly uneven, and most studies were conducted in temperate regions, while tropical and boreal zones are severely underrepresented (Figures 1 and S4). This pronounced spatial bias may limit the global generalizability of our conclusions, especially considering the significant regulatory effect of climatic factors on PE intensity observed in this study (Figure S4). Thus, future research should prioritize expanding empirical PE studies in these underrepresented climatic regions to develop robust, globally applicable predictive models for substrate‐induced PE. In addition, the interactive effects of multiple carbon substrates entering soils simultaneously remain largely unexplored and represent another key knowledge gap. Most existing studies, including those synthesized in this meta‐analysis, only examined the effects of single carbon substrate addition on PE. Future experimental designs should systematically manipulate combinations of different carbon substrates to elucidate their synergistic or antagonistic effects on PE, which would better reflect the complexity of real‐world soil carbon input scenarios (e.g., simultaneous input of plant residues and root exudates in natural ecosystems). Therefore, to accelerate progress in this critical research area, we advocate for the implementation of coordinated, multi‐site field experiments, the establishment of standardized experimental methodologies for PE research, and the promotion of open data sharing among the global soil science community.

## 5 Conclusions

This meta‐analysis elucidates that the positive SOM priming is a widespread phenomenon in soil ecosystems, and its magnitude is closely related to the substrates from specific types of external carbon. Specifically, plant residues induced the greatest PE, followed by root exudates, biochar, and degradable microplastics. Substrate‐specific properties control SOM priming by regulating microbial activity, growth, competition, and microbial life strategies. In particular, non‐woody residues with higher cellulose and hemicellulose contents resulted in greater SOM priming than woody residues with higher lignin contents. Organic acids in root exudates, followed by monosaccharides, polysaccharides, and phenolics, induced the most SOM priming because organic acids released mineral nutrients through soil acidification. Woody‐derived biochar with more aromatic carbon induced more SOM priming than non‐woody biochar. The input of highly degradable polyhydroxyalkanoate microplastics stimulated SOM priming more than other microplastics (polybutylene succinate, polybutylene adipate‐co‐terephthalate, and polylactic acid). These findings highlight the critical role of the type and quality of external carbon substrates in shaping SOM dynamics and provide insight for managing soil carbon sequestration in industrial and agricultural production activities.

## Author Contributions

**Hongxin Dong:** data curation, formal analysis, investigation, visualization, writing – original draft, writing – review and editing. **Yingyi Fu:** formal analysis, validation, investigation. **Shanshan Dai:** visualization, investigation, validation, software. **Peng He:** visualization, validation, formal analysis, resources. **Yu Luo:** investigation, writing – review and editing. **Margaret A. Oliver:** writing – review and editing. **William R. Horwath:** supervision, writing – review and editing. **Lu‐Jun Li:** conceptualization, supervision, project administration, investigation, writing – review and editing.

## Funding

This work was supported by the National Natural Science Foundation of China (U23A6001, 42277350), the State Key Laboratory of Black Soils Conservation and Utilization, Northeast Institute of Geography and Agroecology, Chinese Academy of Sciences (2023HTDGZ‐KF‐01), the International Partnership Project of Chinese Academy of Sciences (131323KYSB20210004), and the Natural Science Foundation of Jilin Province (YDZJ202501ZYTS465).

## Conflicts of Interest

The authors declare no conflicts of interest.

## Supporting information


**Data S1:** gcb70861‐sup‐0001‐Supinfo1.xlsx.

**Data S2:** PRISMA 2020 Checklist.

**Figure S1:** The flow diagram of meta‐analysis describes the information flow in four stages of the system evaluation process (‘identification’, ‘screening’, ‘eligibility’, and ‘included’).
**Figure S2:** The average pyrolysis temperature of woody and non‐woody biochars.
**Figure S3:** The mean effect size of soil organic matter priming in response to carbon input (mean±95% CI, CI is confidence interval) categorized by ecosystem (cropland, grassland, forest, and wetland). QM, heterogeneity in group cumulative effect sizes.
**Figure S4:** The mean effect size of soil organic matter priming in response to carbon input (mean±95% CI, CI is confidence interval) categorized by climatic zones (tropical zone, temperate zone, and boreal zone). QM, heterogeneity in group cumulative effect sizes.
**Figure S5:** Size of average values of four carbon sources on net carbon (C) balance.
**Figure S6:** Graphs of percentage values plotted against for positive and negative effects of four carbon sources (plant residues, root exudates, biochar, and microplastics) on net carbon (C) balance. A positive effect size suggests that four C sources increase soil C content, whereas a negative effect size suggests that four C sources decrease soil C content. The n is sample size, and the number after the comma shows the percentage of positive and negative effects of four C sources on net C balance.
**Table S1:** List of publications used in the study.
**Table S2:** The plant residues, root exudates, biochar and microplastics considered for the effects on soil organic carbon (SOM) priming, **s**ample size (number of observations), results of testing publication bias and random‐effect models for each response variable. Publication bias was tested through fail‐safe number and trim and fill models.

## Data Availability

The data that support the findings of this study are openly available in Figshare at https://doi.org/10.6084/m9.figshare.31908364.

## References

[gcb70861-bib-0001] Armstrong McKay, D. I. , A. Staal , J. F. Abrams , et al. 2022. “Exceeding 1.5°C Global Warming Could Trigger Multiple Climate Tipping Points.” Science 377, no. 6611: eabn7950. 36074831 10.1126/science.abn7950

[gcb70861-bib-0002] Aye, N. S. , C. R. Butterly , P. W. G. Sale , and C. Tang . 2018. “Interactive Effects of Initial pH and Nitrogen Status on Soil Organic Carbon Priming by Glucose and Lignocellulose.” Soil Biology & Biochemistry 123: 33–44.

[gcb70861-bib-0003] Blagodatskaya, E. , and Y. Kuzyakov . 2008. “Mechanisms of Real and Apparent Priming Effects and Their Dependence on Soil Microbial Biomass and Community Structure: Critical Review.” Biology and Fertility of Soils 45, no. 2: 115–131.

[gcb70861-bib-0004] Breiman, L. 2001. “Random Forests.” Machine Learning 45, no. 1: 5–32.

[gcb70861-bib-0005] Chaturvedi, V. , and P. Verma . 2013. “An Overview of Key Pretreatment Processes Employed for Bioconversion of Lignocellulosic Biomass Into Biofuels and Value Added Products.” 3 Biotech 3, no. 5: 415–431. 10.1007/s13205-013-0167-8 PMC3781263 28324338

[gcb70861-bib-0006] Chen, L. , L. Han , F. Wang , et al. 2025. “Polylactic Acid Microplastics Induced Negative Priming and Improved Carbon Sequestration via Microbial Processes in Different Paddy Soils.” Soil Biology & Biochemistry 201: 109653.

[gcb70861-bib-0007] Chen, R. , M. Senbayram , S. Blagodatsky , et al. 2014. “Soil C and N Availability Determine the Priming Effect: Microbial N Mining and Stoichiometric Decomposition Theories.” Global Change Biology 20, no. 7: 2356–2367. 24273056 10.1111/gcb.12475

[gcb70861-bib-0008] Chen, T. J. , L. Y. Li , R. D. Zhao , and J. H. Wu . 2017. “Pyrolysis Kinetic Analysis of the Three Pseudocomponents of Biomass‐Cellulose, Hemicellulose and Lignin.” Journal of Thermal Analysis and Calorimetry 128, no. 3: 1825–1832.

[gcb70861-bib-0009] Chen, X. , J. Lin , P. Wang , S. Zhang , D. Liu , and B. Zhu . 2022. “Resistant Soil Carbon Is More Vulnerable to Priming Effect Than Active Soil Carbon.” Soil Biology & Biochemistry 168: 108619.

[gcb70861-bib-0010] Coward, E. K. , T. Ohno , and D. L. Sparks . 2019. “Direct Evidence for Temporal Molecular Fractionation of Dissolved Organic Matter at the Iron Oxyhydroxide Interface.” Environmental Science and Technology 53, no. 2: 642–650. 30525494 10.1021/acs.est.8b04687

[gcb70861-bib-0011] Deng, J. , T. Y. Xiong , H. Y. Wang , A. M. Zheng , and Y. Wang . 2016. “Effects of Cellulose, Hemicellulose, and Lignin on the Structure and Morphology of Porous Carbons.” ACS Sustainable Chemistry & Engineering 4, no. 7: 3750–3756.

[gcb70861-bib-0012] Dong, H. , P. He , M. Liu , Y. Kuzyakov , and L. J. Li . 2025. “Nitrogen Availability Governs Priming Effect Induced by Biodegradable Microplastics Through Microbial Life‐Strategies.” European Journal of Soil Science 76, no. 4: e70170.

[gcb70861-bib-0013] Dong, H. , J. Lin , J. Lu , et al. 2024. “Priming Effects of Surface Soil Organic Carbon Decreased With Warming: A Global Meta‐Analysis.” Plant and Soil 500, no. 1: 233–242.

[gcb70861-bib-0014] Dong, H. , S. Zhang , J. Lin , and B. Zhu . 2021. “Responses of Soil Microbial Biomass Carbon and Dissolved Organic Carbon to Drying‐Rewetting Cycles: A Meta‐Analysis.” Catena 207: 105610.

[gcb70861-bib-0015] Drake, J. E. , B. Darby , M. Giasson , M. A. Kramer , R. P. Phillips , and A. C. Finzi . 2013. “Stoichiometry Constrains Microbial Response to Root Exudation‐Insights From a Model and a Field Experiment in a Temperate Forest.” Biogeosciences 10, no. 2: 821–838.

[gcb70861-bib-0016] Duval, S. , and R. Tweedie . 2000. “Trim and Fill: A Simple Funnel‐Plot‐Based Method of Testing and Adjusting for Publication Bias in Meta‐Analysis.” Biometrics 56, no. 2: 455–463. 10877304 10.1111/j.0006-341x.2000.00455.x

[gcb70861-bib-0017] Fan, F. , B. Yu , B. Wang , et al. 2019. “Microbial Mechanisms of the Contrast Residue Decomposition and Priming Effect in Soils With Different Organic and Chemical Fertilization Histories.” Soil Biology and Biochemistry 135: 213–221.

[gcb70861-bib-0018] Feng, J. , and B. Zhu . 2021. “Global Patterns and Associated Drivers of Priming Effect in Response to Nutrient Addition.” Soil Biology and Biochemistry 153: 108118.

[gcb70861-bib-0019] Feng, L. , Y. Wang , R. Fensholt , et al. 2025. “Globally Increased Cropland Soil Exposure to Climate Extremes in Recent Decades.” Nature Communications 16, no. 1: 4354. 10.1038/s41467-025-59544-1 PMC12065872 40348795

[gcb70861-bib-0020] Fontaine, S. , C. Henault , A. Aamor , et al. 2011. “Fungi Mediate Long Term Sequestration of Carbon and Nitrogen in Soil Through Their Priming Effect.” Soil Biology and Biochemistry 43, no. 1: 86–96.

[gcb70861-bib-0021] Frey, S. D. , J. Lee , J. M. Melillo , and J. Six . 2013. “The Temperature Response of Soil Microbial Efficiency and Its Feedback to Climate.” Nature Climate Change 3, no. 4: 395–398.

[gcb70861-bib-0022] Fu, Y. , W. Liu , Z. Chen , et al. 2025. “Microbial Metabolisms Determine Soil Priming Effect Induced by Organic Inputs.” Soil Biology and Biochemistry 209: 109885.

[gcb70861-bib-0023] Fu, Y. , Y. Xu , Q. Wang , et al. 2025. “Deciphering the Microbial Players Driving Straw Decomposition and Accumulation in Soil Components of Particulate and Mineral‐Associated Organic Matter.” Soil Biology and Biochemistry 209: 109871.

[gcb70861-bib-0024] Giehl, R. F. H. , and N. von Wirén . 2014. “Root Nutrient Foraging.” Plant Physiology 166, no. 2: 509–517. 25082891 10.1104/pp.114.245225 PMC4213083

[gcb70861-bib-0025] Girish, C. R. , and V. R. Murty . 2012. “Review of Various Treatment Methods for the Abatement of Phenolic Compounds From Wastewater.” Journal of Environmental Science & Engineering 54, no. 2: 306–316. 24749384

[gcb70861-bib-0026] Grimm, C. C. , D. A. Grimm , and C. J. Bergman . 2003. “The Analysis of Oligosaccharides by Mass Spectrometry.” In Oligosaccharides in Food and Agriculture, edited by G. Eggleston and G. L. Cote , 32–42. ACS Symposium Series.

[gcb70861-bib-0027] Grundmann, V. , B. Bilitewski , A. Zentner , C. R. Wonschik , and M. Focke . 2013. “Hydrolysis and Anaerobic co‐Fermentation of Different Kinds of Biodegradable Polymers.” Waste and Biomass Valorization 4, no. 2: 371–376.

[gcb70861-bib-0028] Han, X. , Y. Zhao , Y. Chen , et al. 2022. “Lignin Biosynthesis and Accumulation in Response to Abiotic Stresses in Woody Plants.” Forest Research 2: 9. 10.48130/FR-2022-0009 PMC11524291 39525415

[gcb70861-bib-0029] He, M. , Y. Jiang , Y. Han , et al. 2023. “Rice Straw Biochar Is More Beneficial to Soil Organic Carbon Accumulation and Stabilization Than Rice Dtraw and Rice Straw Ash.” Journal of Soil Science and Plant Nutrition 23: 3023–3033.

[gcb70861-bib-0030] He, P. , L.‐J. Li , S. S. Dai , et al. 2024. “Straw Addition and Low Soil Moisture Decreased Temperature Sensitivity and Activation Energy of Soil Organic Matter.” Geoderma 442: 116802.

[gcb70861-bib-0031] Hilscher, A. , K. Heister , C. Siewert , and H. Knicker . 2009. “Mineralisation and Structural Changes During the Initial Phase of Microbial Degradation of Pyrogenic Plant Residues in Soil.” Organic Geochemistry 40, no. 3: 332–342.

[gcb70861-bib-0032] Hu, C. , T. Xu , S. Wang , H. Bian , and H. Dai . 2024. “Effect of Aminating Lignin Loading With Arbuscular Mycorrhizal Fungi on Soil Aggregate Structure Improvement.” Polymers 16, no. 12: 1701. 38932051 10.3390/polym16121701 PMC11207646

[gcb70861-bib-0033] Hu, Z. , M. Delgado‐Baquerizo , N. Fanin , et al. 2024. “Nutrient‐Induced Acidification Modulates Soil Biodiversity‐Function Relationships.” Nature Communications 15, no. 1: 2858. 10.1038/s41467-024-47323-3 PMC10991381 38570522

[gcb70861-bib-0034] Huang, W. , and X. Xia . 2024. “Element Cycling With Micro(Nano)plastics.” Science 385, no. 6712: 933–935. 39208108 10.1126/science.adk9505

[gcb70861-bib-0035] Huo, C. , Y. Luo , and W. Cheng . 2017. “Rhizosphere Priming Effect: A Meta‐Analysis.” Soil Biology and Biochemistry 111: 78–84.

[gcb70861-bib-0036] Huo, Y. , F. A. Dijkstra , M. Possell , and B. Singh . 2024. “Mineralisation and Priming Effects of a Biodegradable Plastic Mulch Film in Soils: Influence of Soil Type, Temperature and Plastic Particle Size.” Soil Biology and Biochemistry 189: 109257.

[gcb70861-bib-0037] Islam, M. R. , B. Bicharanloo , X. Yu , B. Singh , and F. A. Dijkstra . 2025. “Rhizodeposition Stimulates Soil Carbon Decomposition and Promotes Formation of Mineral‐Associated Carbon With Increased Clay Content.” Geoderma 454: 117180.

[gcb70861-bib-0038] Jia, K. , S. Nie , M. Tian , et al. 2024. “Biodegradable Microplastics Can Cause More Serious Loss of Soil Organic Carbon by Priming Effect Than Conventional Microplastics in Farmland Shelterbelts.” Functional Ecology 38, no. 11: 2447–2458.

[gcb70861-bib-0039] Jin, X. , L. Wang , J. Zhang , et al. 2024. “Two Decades of no Tillage Divergently Accumulate Plant Lignin and Microbial Necromass in the Top and Sublayers.” Soil and Tillage Research 244: 106211.

[gcb70861-bib-0040] Kou, Z. N. , D. Tolmachev , M. Vuorte , and M. Sammalkorpi . 2025. “Component Size Dependent Lignin‐Carbohydrate Complex Adsorption at Crystalline Cellulose Surfaces.” Cellulose 32, no. 2: 983–997.

[gcb70861-bib-0041] Kreft, J. U. , and S. Bonhoeffer . 2005. “The Evolution of Groups of Cooperating Bacteria and the Growth Rate Versus Yield Trade‐Off.” Microbiology 151, no. 3: 637–641. 15758209 10.1099/mic.0.27415-0

[gcb70861-bib-0042] Kreft, J. U. , B. M. Griffin , and R. González‐Cabaleiro . 2020. “Evolutionary Causes and Consequences of Metabolic Division of Labour: Why Anaerobes Do and Aerobes Don't.” Current Opinion in Biotechnology 62: 80–87. 31654858 10.1016/j.copbio.2019.08.008

[gcb70861-bib-0043] Kuzyakov, Y. , J. K. Friedel , and K. Stahr . 2000. “Review of Mechanisms and Quantification of Priming Effects.” Soil Biology and Biochemistry 32, no. 11: 1485–1498.

[gcb70861-bib-0044] Lehmann, J. , and M. Kleber . 2015. “The Contentious Nature of Soil Organic Matter.” Nature 528, no. 7580: 60–68. 26595271 10.1038/nature16069

[gcb70861-bib-0045] Li, P. F. , M. Wu , T. Li , et al. 2023. “Molecular Weight of Dissolved Organic Matter Determines Its Interactions With Microbes and Its Assembly Processes in Soils.” Soil Biology and Biochemistry 184: 109117.

[gcb70861-bib-0046] Li, Y. , Q. Yan , C. Zou , et al. 2025. “Microplastic‐Induced Alterations in Soil Aggregate‐Associated Carbon Stabilization Pathways: Evidence From δ^13^C Signature Analysis.” Environmental Science & Technology 59, no. 11: 5545–5555. 40070098 10.1021/acs.est.4c09242

[gcb70861-bib-0047] Lin, J. , B. Chen , H. Dong , et al. 2025. “Effects of Soil Moisture Fluctuation and Microplastics Types on Soil Organic Matter Decomposition and Carbon Dynamics.” Soil Biology and Biochemistry 205: 109781.

[gcb70861-bib-0048] Liu, Y. , W. Li , H. Meng , et al. 2025. “Contributions of Microbial Necromass and Plant Lignin to Soil Organic Carbon Stock in a Paddy Field Under Simulated Conditions of Long‐Term Elevated CO_2_ and Warming.” Soil Biology and Biochemistry 201: 109649.

[gcb70861-bib-0049] Mason‐Jones, K. , and Y. Kuzyakov . 2017. “‘Non‐Metabolizable’ Glucose Analogue Shines New Light on Priming Mechanisms: Triggering of Microbial Metabolism.” Soil Biology and Biochemistry 107: 68–76.

[gcb70861-bib-0050] Mehnaz, K. R. , P. E. Corneo , C. Keitel , and F. A. Dijkstra . 2019. “Carbon and Phosphorus Addition Effects on Microbial Carbon Use Efficiency, Soil Organic Matter Priming, Gross Nitrogen Mineralization and Nitrous Oxide Emission From Soil.” Soil Biology and Biochemistry 134: 175–186.

[gcb70861-bib-0051] Mikajlo, I. , T. Z. Lerch , B. Louvel , J. Hynst , J. Zahora , and B. Pourrut . 2024. “Composted Biochar Versus Compost With Biochar: Effects on Soil Properties and Plant Growth.” Biochar 6, no. 1: 85.

[gcb70861-bib-0052] Murtaza, G. , M. Usman , J. Iqbal , et al. 2024. “Liming Potential and Characteristics of Biochar Produced From Woody and Non‐Woody Biomass at Different Pyrolysis Temperatures.” Scientific Reports 14, no. 1: 11469. 38769392 10.1038/s41598-024-61974-8 PMC11106251

[gcb70861-bib-0053] Nakae, T. 1976. “Outer Membrane of Salmonella—Isolation of Protein Complex That Produces Transmembrane Channels.” Journal of Biological Chemistry 251, no. 7: 2176–2178. 773934

[gcb70861-bib-0054] Ornaghi, H. L., Jr. , F. M. Monticeli , R. M. Neves , L. D. Agnol , and O. Bianchi . 2023. “Influence of Different Cellulose/Hemicellulose/Lignin Ratios on the Thermal Degradation Behavior: Prediction and Optimization.” Biomass Conversion and Biorefinery 13, no. 9: 7775–7782.

[gcb70861-bib-0055] Parajuli, B. , R. Ye , and A. Szogi . 2022. “Mineral N Suppressed Priming Effect While Increasing Microbial C Use Efficiency and N_2_O Production in Sandy Soils Under Long‐Term Conservation Management.” Biology and Fertility of Soils 58, no. 8: 903–915.

[gcb70861-bib-0056] Pastor, J. M. , N. Borges , J. P. Pagán , et al. 2019. “Fructose Metabolism in *Chromohalobacter salexigens* : Interplay Between the Embden–Meyerhof–Parnas and Entner–Doudoroff Pathways.” Microbial Cell Factories 18, no. 1: 134. 31409414 10.1186/s12934-019-1178-x PMC6692947

[gcb70861-bib-0057] Poeplau, C. , R. Dechow , N. Begill , and A. Don . 2024. “Towards an Ecosystem Capacity to Stabilise Organic Carbon in Soils.” Global Change Biology 30, no. 8: e17453. 39099457 10.1111/gcb.17453

[gcb70861-bib-0058] Preston, C. M. , and M. W. I. Schmidt . 2006. “Black (Pyrogenic) Carbon: A Synthesis of Current Knowledge and Uncertainties With Special Consideration of Boreal Regions.” Biogeosciences 3, no. 4: 397–420.

[gcb70861-bib-0059] Reddy, N. , and Y. Q. Yang . 2009. “Properties and Potential Applications of Natural Cellulose Fibers From the Bark of Cotton Stalks.” Bioresource Technology 100, no. 14: 3563–3569. 19327987 10.1016/j.biortech.2009.02.047

[gcb70861-bib-0060] Rosenthal, R. 1979. “The File Drawer Problem and Tolerance for Null Results.” Psychological Bulletin 86, no. 3: 638–641.

[gcb70861-bib-0061] Ryu, Y. , F. E. Bouharras , M. Cha , et al. 2025. “Recent Advancements in the Evolution, Production, and Degradation of Biodegradable Mulch Films: A Review.” Environmental Research 277: 121629. 40250592 10.1016/j.envres.2025.121629

[gcb70861-bib-0062] Santos, F. , D. M. Rice , J. A. Bird , and A. A. Berhe . 2021. “Pyrolysis Temperature and Soil Depth Interactions Determine PyC Turnover and Induced Soil Organic Carbon Priming.” Biogeochemistry 153, no. 1: 47–65.

[gcb70861-bib-0063] Schmidt, M. W. I. , M. S. Torn , S. Abiven , et al. 2011. “Persistence of Soil Organic Matter as an Ecosystem Property.” Nature 478, no. 7367: 49–56. 21979045 10.1038/nature10386

[gcb70861-bib-0064] Shi, J. , Q. Zhang , Y. Sun , Y. Peng , J. Wang , and X. Wang . 2025. “Microplastic Induces Microbial Nitrogen Limitation Further Alters Microbial Nitrogentransformation: Insights From Metagenomic Analysis.” Science of the Total Environment 967: 178825. 39946886 10.1016/j.scitotenv.2025.178825

[gcb70861-bib-0065] Singh, B. P. , and A. L. Cowie . 2014. “Long‐Term Influence of Biochar on Native Organic Carbon Mineralisation in a Low‐Carbon Clayey Soil.” Scientific Reports 4: 3687. 24446050 10.1038/srep03687 PMC3896930

[gcb70861-bib-0066] Singh, B. P. , A. L. Cowie , and R. J. Smernik . 2012. “Biochar Carbon Stability in a Clayey Soil as a Function of Feedstock and Pyrolysis Temperature.” Environmental Science and Technology 46, no. 21: 11770–11778. 23013285 10.1021/es302545b

[gcb70861-bib-0067] Tanunchai, B. , M. Schädler , and M. Noll . 2025. “Future Climate and Agricultural Farming Systems Affect the Fungal Plastisphere of Different Biodegradable Plastics at the Early Stage of Field Degradation.” Environmental Sciences Europe 37, no. 1: 23.

[gcb70861-bib-0068] Tao, F. , Y. Huang , B. A. Hungate , et al. 2023. “Microbial Carbon Use Efficiency Promotes Global Soil Carbon Storage.” Nature 618, no. 7967: 981–985. 37225998 10.1038/s41586-023-06042-3 PMC10307633

[gcb70861-bib-0069] Tao, L. , L. Yin , F. A. Dijkstra , J. Lin , P. Wang , and W. Cheng . 2026. “Differences in Priming Effect of Topsoil Versus Subsoil Depends on Ecosystem Type.” Catena 263: 109766.

[gcb70861-bib-0070] Viechtbauer, W. 2010. “Conducting Meta‐Analyses in R With the Metafor Package.” Journal of Statistical Software 36, no. 3: 1–48.

[gcb70861-bib-0071] von Luetzow, M. , I. Koegel‐Knabner , K. Ekschmitt , et al. 2006. “Stabilization of Organic Matter in Temperate Soils: Mechanisms and Their Relevance Under Different Soil Conditions: A Review.” European Journal of Soil Science 57, no. 4: 426–445.

[gcb70861-bib-0072] Wang, C. , and Y. Kuzyakov . 2024a. “Mechanisms and Implications of Bacterial–Fungal Competition for Soil Resources.” ISME Journal 18, no. 1: wrae073. 38691428 10.1093/ismejo/wrae073 PMC11104273

[gcb70861-bib-0073] Wang, C. , and Y. Kuzyakov . 2024b. “Soil Organic Matter Priming: The pH Effects.” Global Change Biology 30, no. 6: e17349. 38822665 10.1111/gcb.17349

[gcb70861-bib-0074] Wang, C. , B. Zhu , Y. Luo , and Y. Kuzyakov . 2026. “100 Years of Soil Organic Matter Priming Research.” Global Change Biology 32, no. 1: e70674. 41472643 10.1111/gcb.70674

[gcb70861-bib-0075] Wang, J. , Z. Xiong , and Y. Kuzyakov . 2016. “Biochar Stability in Soil: Meta‐Analysis of Decomposition and Priming Effects.” GCB Bioenergy 8, no. 3: 512–523.

[gcb70861-bib-0076] Wang, S. , W. Gao , Z. Ma , et al. 2024. “Iron Mineral Type Controls Organic Matter Stability and Priming in Paddy Soil Under Anaerobic Conditions.” Soil Biology and Biochemistry 197: 109518.

[gcb70861-bib-0077] Wang, X. , J. Lu , X. Zhang , and P. Wang . 2021. “Contrasting Microbial Mechanisms of Soil Priming Effects Induced by Crop Residues Depend on Nitrogen Availability and Temperature.” Applied Soil Ecology 168: 104186.

[gcb70861-bib-0078] Wang, Y. , X. Liu , W. Han , et al. 2024. “Migration and Transformation Modes of Microplastics in Reclaimed Wastewater Treatment Plant and Sludge Treatment Center With Thermal Hydrolysis and Anaerobic Digestion.” Bioresource Technology 400: 130649. 38570098 10.1016/j.biortech.2024.130649

[gcb70861-bib-0079] Wen, H. , F. Yang , Z. Sun , Z. Miao , J. Hu , and G. Zhang . 2025. “Asymmetric Responses of Soil Organic Carbon Stability to Shifting Dominance of pH‐Mediated Metal‐Bound Organic Carbon.” Communications Earth & Environment 6, no. 1: 574.

[gcb70861-bib-0080] Weng, Y. X. , L. Wang , M. Zhang , X.‐L. Wang , and Y. Z. Wang . 2013. “Biodegradation Behavior of P(3HB,4HB)/PLA Blends in Real Soil Environments.” Polymer Testing 32, no. 1: 60–70.

[gcb70861-bib-0081] Wiesenbauer, J. , S. Gorka , K. Jenab , et al. 2025. “Preferential Use of Organic Acids Over Sugars by Soil Microbes in Simulated Root Exudation.” Soil Biology & Biochemistry 203: 109738.

[gcb70861-bib-0082] Wild, B. , S. Alaei , P. Bengtson , et al. 2017. “Short‐Term Carbon Input Increases Microbial Nitrogen Demand, but Not Microbial Nitrogen Mining, in a Set of Boreal Forest Soils.” Biogeochemistry 136, no. 3: 261–278.

[gcb70861-bib-0083] Wu, J. , P. C. Brookes , and D. S. Jenkinson . 1993. “Formation and Destruction of Microbial Biomass During the Decomposition of Glucose and Ryegrass in Soil.” Soil Biology & Biochemistry 25, no. 10: 1435–1441.

[gcb70861-bib-0084] Wu, J. , P. Zhou , L. Li , Y. Su , H. Yuan , and J. K. Syers . 2012. “Restricted Mineralization of Fresh Organic Materials Incorporated Into a Subtropical Paddy Soil.” Journal of the Science of Food and Agriculture 92, no. 5: 1031–1037. 21993911 10.1002/jsfa.4645

[gcb70861-bib-0085] Wu, L. , W. Zhang , W. Wei , et al. 2019. “Soil Organic Matter Priming and Carbon Balance After Straw Addition Is Regulated by Long‐Term Fertilization.” Soil Biology & Biochemistry 135: 383–391.

[gcb70861-bib-0086] Xiao, K. Q. , C. Liang , Z. Wang , et al. 2024. “Beyond Microbial Carbon Use Efficiency.” National Science Review 11, no. 4: nwae059. 38487496 10.1093/nsr/nwae059 PMC10939436

[gcb70861-bib-0087] Xiao, K. Q. , Y. Zhao , C. Liang , et al. 2023. “Introducing the Soil Mineral Carbon Pump.” Nature Reviews Earth & Environment 4, no. 3: 135–136.

[gcb70861-bib-0088] Xu, S. , M. Delgado‐Baquerizo , Y. Kuzyakov , et al. 2024. “Positive Soil Priming Effects Are the Rule at a Global Scale.” Global Change Biology 30, no. 9: e17502. 39252425 10.1111/gcb.17502

[gcb70861-bib-0089] Xu, S. , M. Na , Y. Huang , J. Zhang , J. Zhou , and L. J. Li . 2025. “Changes in Microbial Carbon Cycling Functions Along Rice Cultivation Chronosequences in Saline‐Alkali Soils.” Soil Biology and Biochemistry 202: 109699.

[gcb70861-bib-0090] Xu, T. , X. Wang , Q. Shi , H. Liu , Y. Chen , and J. Liu . 2024. “Review of Soil Microplastic Degradation Pathways and Remediation Techniques.” International Journal of Environmental Research 18, no. 5: 77.

[gcb70861-bib-0091] Yan, S. , L. Yin , F. A. Dijkstra , P. Wang , and W. Cheng . 2023. “Priming Effect on Soil Carbon Decomposition by Root Exudate Surrogates: A Meta‐Analysis.” Soil Biology and Biochemistry 178: 108955.

[gcb70861-bib-0092] Yeo, J. Y. , B. L. F. Chin , J. K. Tan , and Y. S. Loh . 2019. “Comparative Studies on the Pyrolysis of Cellulose, Hemicellulose, and Lignin Based on Combined Kinetics.” Journal of the Energy Institute 92, no. 1: 27–37.

[gcb70861-bib-0093] Yeul, V. S. , and S. S. Rayalu . 2013. “Unprecedented Chitin and Chitosan: A Chemical Overview.” Journal of Polymers and the Environment 21, no. 2: 606–614.

[gcb70861-bib-0094] Zhang, G. , D. Liu , J. Lin , et al. 2023. “Priming Effects Induced by Degradable Microplastics in Agricultural Soils.” Soil Biology and Biochemistry 180: 109006.

[gcb70861-bib-0095] Zhang, S. , W. Yang , H. Chen , B. Liu , B. Lin , and Y. Tao . 2019. “Metabolic Engineering for Efficient Supply of Acetyl‐CoA From Different Carbon Sources in *Escherichia coli* .” Microbial Cell Factories 18, no. 1: 130. 31387584 10.1186/s12934-019-1177-y PMC6685171

[gcb70861-bib-0096] Zhang, S. , Z. Yu , J. Lin , and B. Zhu . 2020. “Responses of Soil Carbon Decomposition to Drying‐Rewetting Cycles: A Meta‐Analysis.” Geoderma 361: 114069.

[gcb70861-bib-0097] Zhang, Y. , H. Hu , Y. Ran , et al. 2025. “Enhanced Priming Effect in Agricultural Soils Driven by High‐Quality Exogenous Organic Carbon Additions: A Meta‐Analysis.” Science of the Total Environment 962: 178387. 39799648 10.1016/j.scitotenv.2025.178387

[gcb70861-bib-0098] Zhao, S. , M. C. Rillig , H. Bing , et al. 2024. “Microplastic Pollution Promotes Soil Respiration: A Global‐Scale Meta‐Analysis.” Global Change Biology 30, no. 7: e17415. 39005227 10.1111/gcb.17415

[gcb70861-bib-0099] Zhou, S. , J. Lin , P. Wang , P. Zhu , and B. Zhu . 2023. “Resistant Soil Organic Carbon Is More Vulnerable to Priming by Root Exudate Fractions Than Relatively Active Soil Organic Carbon.” Plant and Soil 488, no. 1: 71–82.

[gcb70861-bib-0100] Zhu, B. , and W. Cheng . 2011. “Rhizosphere Priming Effect Increases the Temperature Sensitivity of Soil Organic Matter Decomposition.” Global Change Biology 17, no. 6: 2172–2183.

[gcb70861-bib-0101] Zhu, X. , B. Chen , L. Zhu , and B. Xing . 2017. “Effects and Mechanisms of Biochar‐Microbe Interactions in Soil Improvement and Pollution Remediation: A Review.” Environmental Pollution 227: 98–115. 28458251 10.1016/j.envpol.2017.04.032

[gcb70861-bib-0102] Zhu, X. F. , R. D. Jackson , E. H. DeLucia , J. M. Tiedje , and C. Liang . 2020. “The Soil Microbial Carbon Pump: From Conceptual Insights to Empirical Assessments.” Global Change Biology 26, no. 11: 6032–6039. 32844509 10.1111/gcb.15319

