# Supplementary material for: Priming Effects on Soil Organic Matter Mineralization by Carbon Substrates: A Global Meta‐Analysis
Source: Glob Chang Biol. 2026 Apr 10;32(4):e70861. doi: 10.1111/gcb.70861 (PMC13069200; doi:10.1111/gcb.70861)
Supplement: Supplementary file 3 — Figure S1: The flow diagram of meta‐analysis describes the information flow in four stages of the system evaluation process (‘identification’, ‘screening’, ‘eligibility’, and ‘included’). Figure S2: The average pyrolysis temperature of woody and non‐woody biochars. Figure S3: The mean effect size of soil organic matter priming in response to carbon input (mean±95% CI, CI is confidence interval) categorized by ecosystem (cropland, grassland, forest, and wetland). QM, heterogeneity in group cumulative effect sizes. Figure S4: The mean effect size of soil organic matter priming in response to carbon input (mean±95% CI, CI is confidence interval) categorized by climatic zones (tropical zone, temperate zone, and boreal zone). QM, heterogeneity in group cumulative effect sizes. Figure S5: Size of average values of four carbon sources on net carbon (C) balance. Figure S6: Graphs of percentage values plotted against for positive and negative effects of four carbon sources (plant residues, root exudates, biochar, and microplastics) on net carbon (C) balance. A positive effect size suggests that four C sources increase soil C content, whereas a negative effect size suggests that four C sources decrease soil C content. The n is sample size, and the number after the comma shows the percentage of positive and negative effects of four C sources on net C balance. Table S1: List of publications used in the study. Table S2: The plant residues, root exudates, biochar and microplastics considered for the effects on soil organic carbon (SOM) priming, sample size (number of observations), results of testing publication bias and random‐effect models for each response variable. Publication bias was tested through fail‐safe number and trim and fill models. [file GCB-32-e70861-s002.docx]

**Supplementary information**


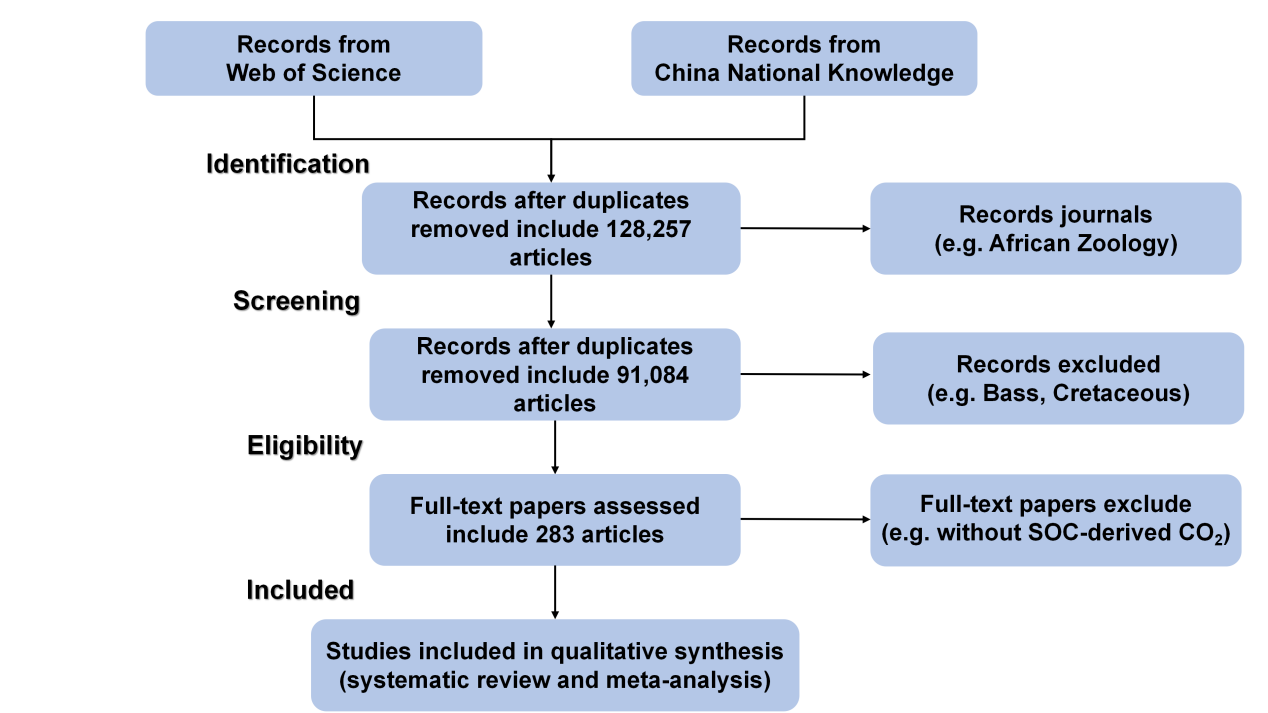


**Fig. S1** The flow diagram of meta-analysis describes the information flow in four stages of the system evaluation process (‘identification’, ‘screening’, ‘eligibility’, and ‘included’).

**

**

**Fig. S2** The average pyrolysis temperature of woody and non-woody biochars.


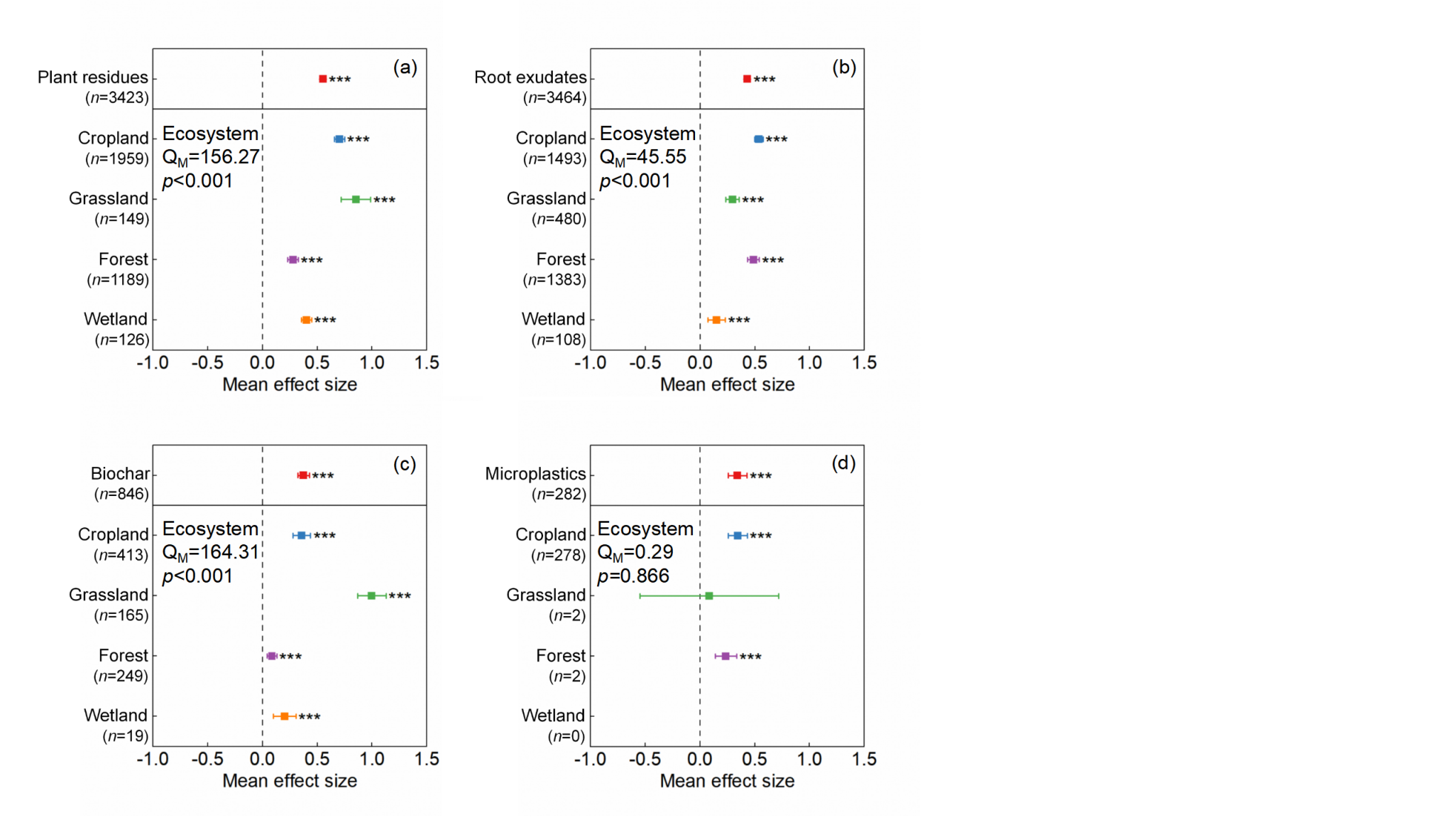


**Fig. S3** The mean effect size of soil organic matter priming in response to carbon input (mean ± 95% CI, CI is confidence interval) categorized by ecosystem (cropland, grassland, forest, and wetland). Q_M_, heterogeneity in group cumulative effect sizes.

**
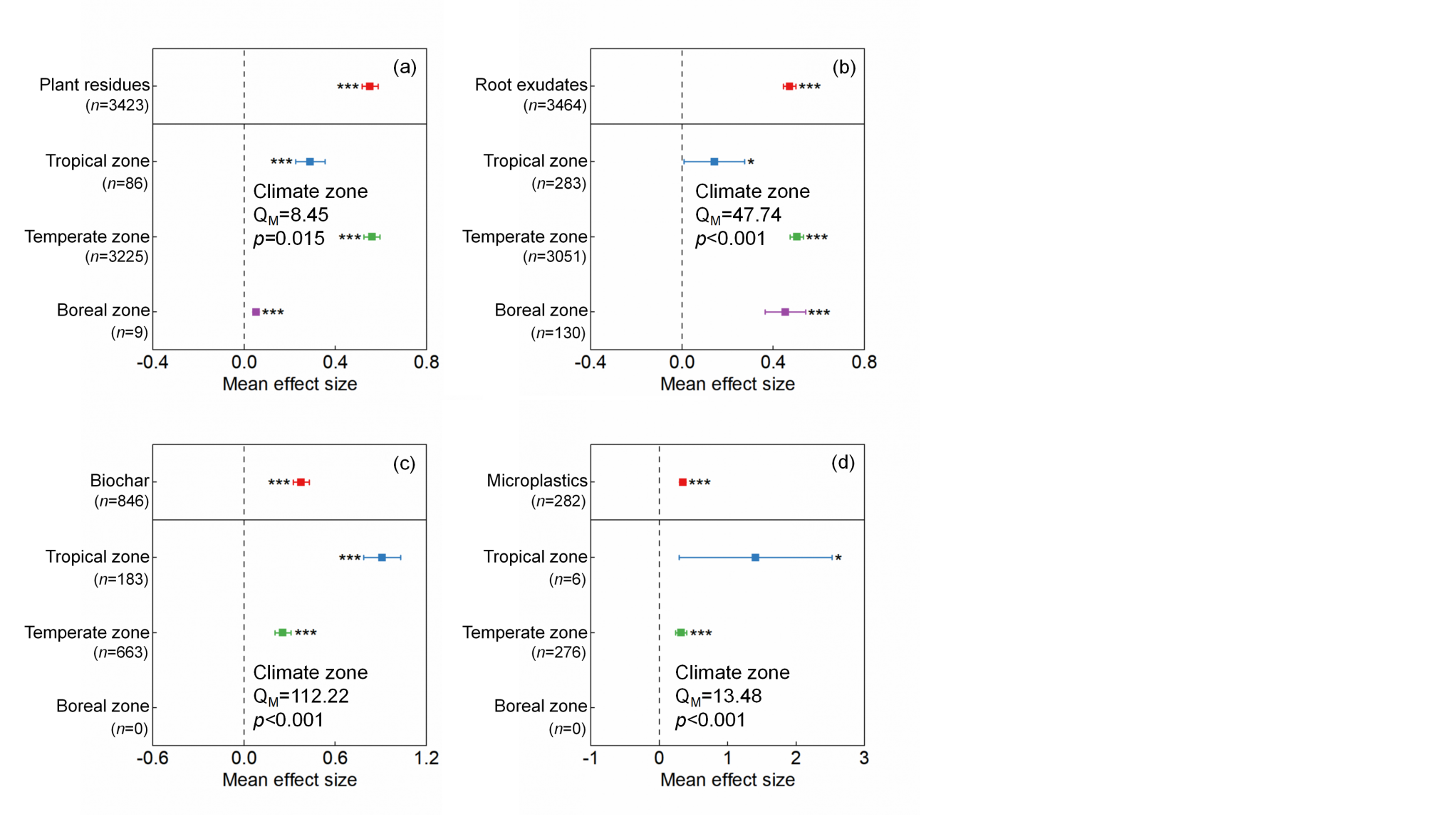
**

**Fig. S4** The mean effect size of soil organic matter priming in response to carbon input (mean ± 95% CI, CI is confidence interval) categorized by climatic zones (tropical zone, temperate zone, and boreal zone). Q_M_, heterogeneity in group cumulative effect sizes.

**
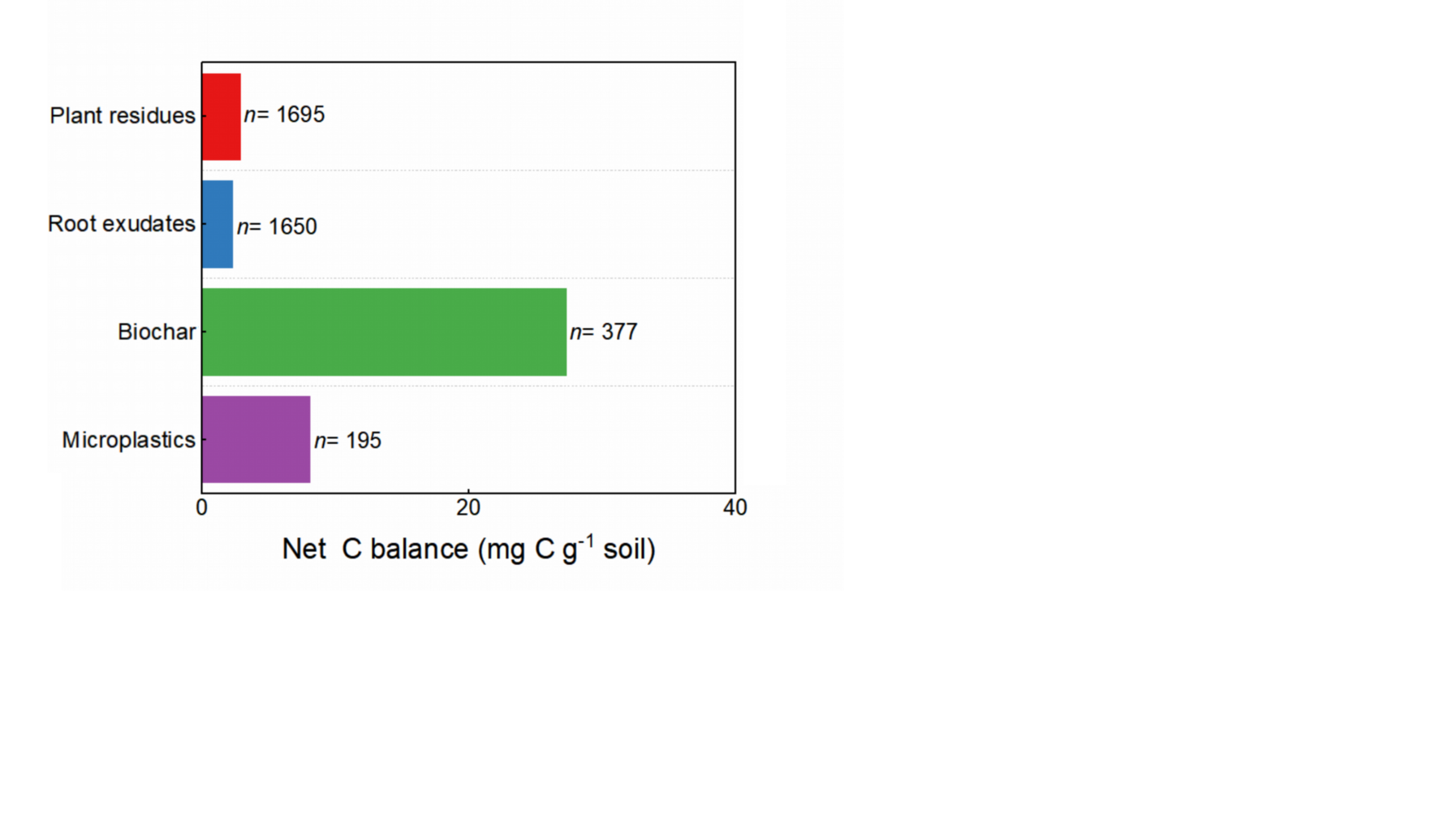
**

**Fig. S5** Size of average values of four carbon sources on net carbon (C) balance.

**
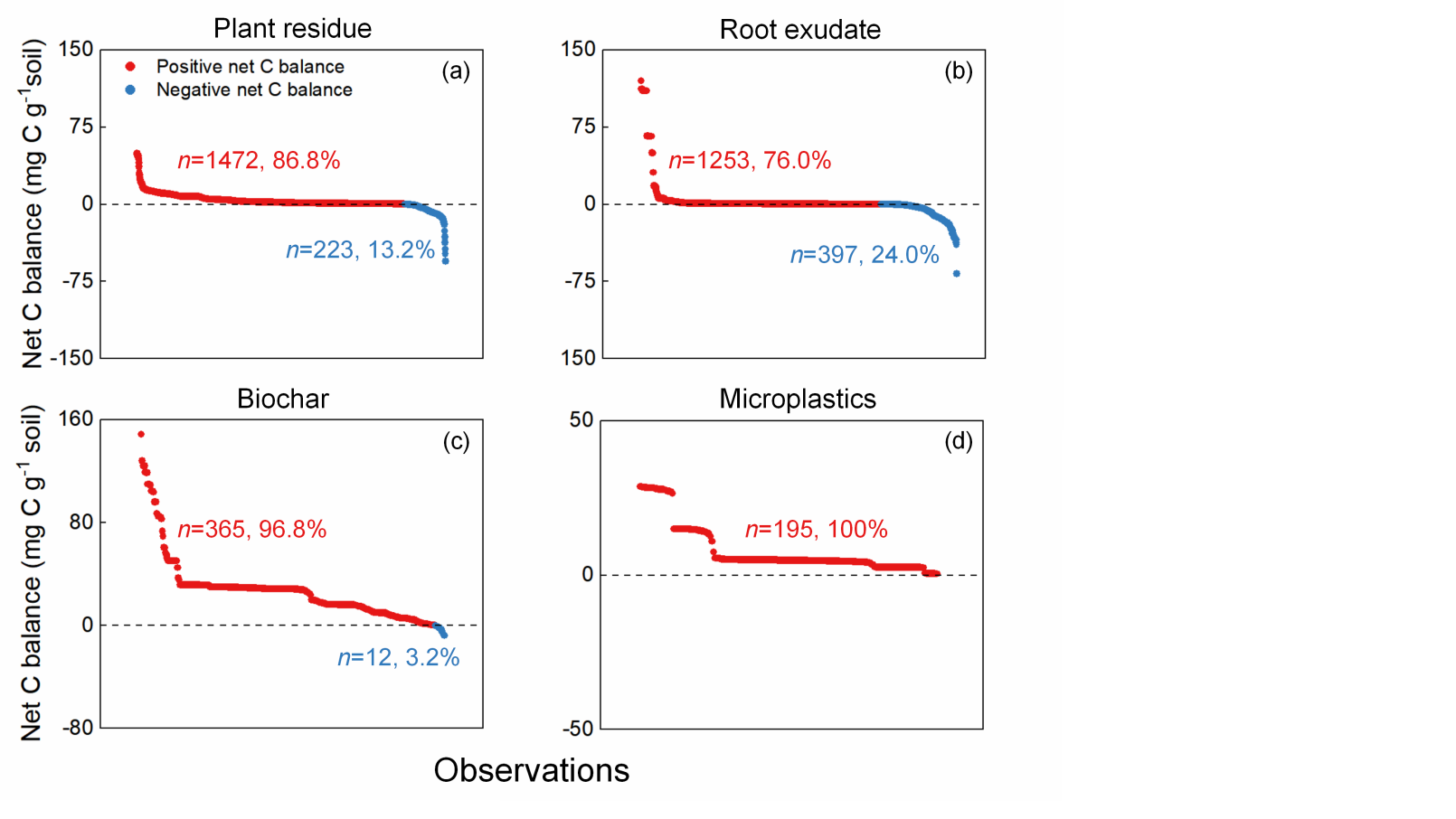
**

**Fig. S6** Graphs of percentage values plotted against for positive and negative effects of four carbon sources (plant residues, root exudates, biochar, and microplastics) on net carbon (C) balance. A positive effect size suggests that four C sources increase soil C content, whereas a negative effect size suggests that four C sources decrease soil C content. The *n* is sample size, and the number after the comma shows the percentage of positive and negative effects of four C sources on net C balance.

**Table S1** List of publications used in the study.

| **No.** | **First author** | **Year** | **Ecosystem** | **Carbon type** | **Substrate type** |
| --- | --- | --- | --- | --- | --- |
| 1 | Tian Chen | 2022 | Forest | Plant residues | Woody residue |
| 2 | Huifang Yuan | 2024 | Forest | Plant residues | Woody residue |
| 3 | Aohan Tian | 2023 | Cropland | Biochar | Crop biochar |
|  |  |  |  |  | Herbaceous biochar |
| 4 | Yulan Tao | 2025 | Forest | Plant residues | Woody residue |
|  |  |  |  | Root exudates | Monosaccharide |
| 5 | Xiaohan Lou | 2024 | Cropland | Plant residues | Woody residue |
| 6 | Min Xu | 2022 | Forest | Root exudates | Monosaccharide |
| 7 | Kongcan Mei | 2022 | Forest | Plant residues | Woody residue |
| 8 | Zhongjing Dai | 2012 | Grassland | Root exudates | Monosaccharide |
| 9 | Zhaoan Sun | 2021 | Cropland | Plant residues | Crop residue |
| 10 | Lin Cao | 2024 | Cropland | Plant residues | Woody residue |
| 11 | Yulam Tao | 2025 | Forest | Plant residues | Woody residue |
|  |  |  |  | Root exudates | Monosaccharide |
| 12 | Zheng Zhang | 2017 | Forest | Plant residues | Woody residue |
| 13 | Guangmu Tang | 2019 | Cropland | Biochar | Crop biochar |
| 14 | Yun Li | 2021 | Wetland | Root exudates | Monosaccharide |
| 15 | Xiaofeng Wang | 2013 | Forest | Plant residues | Woody residue |
| 16 | Xiaorong Lu | 2020 | Forest | Plant residues | Woody residue |
|  |  |  |  | Biochar | Woody biochar |
| 17 | Liang Wang | 2024 | Forest | Plant residues | Woody residue |
| 18 | Benjuan Liu | 2021 | Cropland | Plant residues | Crop residue |
|  |  |  |  | Biochar | Crop biochar |
| 19 | Mengli Li | 2022 | Cropland | Plant residues | Crop residue |
| 20 | Yunfa Qiao | 2024 | Cropland | Plant residues | Crop residue |
| 21 | Jiayu Li | 2021 | Grassland | Root exudates | Monosaccharide |
| 22 | Xuemei Huang | 2023 | Cropland | Plant residues | Woody residue |
| 23 | Yu Luo | 2014 | Cropland | Biochar | Herbaceous biochar |
| 24 | Lening Hu | 2011 | Forest | Plant residues | Crop residue |
| 25 | Xuemei Leng | 2022 | Cropland | Plant residues | Crop residue |
| 26 | Sujie Miao | 2019 | Cropland | Plant residues | Crop residue |
| 27 | Yuting Zhang | 2024 | Forest | Root exudates | Monosaccharide |
| 28 | Yunqiu Wang | 2022 | Cropland | Root exudates | Organic acids |
| 29 | Dianqing Lu | 2007 | Cropland | Root exudates | Monosaccharide |
|  |  |  |  | Plant residues | Crop residue |
| 30 | Na Li | 2023 | Cropland | Root exudates | Monosaccharide |
|  |  |  |  | Root exudates | Organic acids |
| 31 | Lixin Chen | 2017 | Forest | Plant residues | Woody residue |
| 32 | Mengruo Wang | 2013 | Grassland | Plant residues | Herbaceous residue |
| 33 | Jianjun Duan | 2022 | Cropland | Plant residues | Crop residue |
| 34 | Ziying Gan | 2022 | Forest | Plant residues | Woody residue |
| 35 | Zenzu Tang | 2017 | Cropland | Root exudates | Monosaccharide |
| 36 | Qingyan Qiu | 2019 | Forest | Root exudates | Monosaccharide |
| 37 | Hao Wang | 2020 | Forest | Root exudates | Monosaccharide |
| 38 | Aimeng Li | 2019 | Cropland | Plant residues | Crop residue |
| 39 | Feng Liu | 2022 | Cropland | Plant residues | Crop residue |
| 40 | Jialin Jiang | 2021 | Cropland | Root exudates | Monosaccharide |
| 41 | Junrui Chen | 2024 | Forest | Biochar | Crop biochar |
| 42 | Yuanyun Wei | 2019 | Cropland | Root exudates | Monosaccharide |
|  |  |  | Wetland |  |  |
| 43 | Shuangshuang Huang | 2019 | Cropland | Root exudates | Monosaccharide |
| 44 | Jingyue Xue | 2014 | Cropland | Root exudates | Monosaccharide |
|  |  |  | Forest |  |  |
| 45 | Chang Liao | 2016 | Forest | Root exudates | Monosaccharide |
| 46 | Shufen Yuan | 2015 | Forest | Root exudates | Monosaccharide |
|  |  |  |  | Plant residues | Woody residue |
| 47 | Yifan Zhu | 2020 | Forest | Biochar | Woody biochar |
| 48 | Ying Zhang | 2019 | Cropland | Plant residues | Crop residue |
|  |  |  |  | Biochar | Crop biochar |
| 49 | Shiqi Zhang | 2025 | Wetland | Plant residues | Woody residue |
| 50 | Haoyang Wu | 2025 | Grassland | Root exudates | Monosaccharide |
| 51 | Congyue Hu | 2025 | Wetland | Root exudates | Monosaccharide |
| 52 | Yang Xin | 2025 | Cropland | Plant residues | Crop residue |
|  |  |  |  | Biochar | Crop biochar |
| 53 | Changyi Lu | 2025 | Forest | Microplastics | Polybutylene succinate |
|  |  |  | Cropland |  |  |
|  |  |  | Grassland |  |  |
| 54 | Wenhao Jin | 2025 | Forest | Plant residues | Woody residue |
| 55 | Fenliang Fan | 2019 | Cropland | Plant residues | Crop residue |
| 56 | Xuhui Zhou | 2025 | Cropland | Biochar | Crop biochar |
| 57 | Weiwei Lu | 2021 | Forest | Biochar | Crop biochar |
| 58 | Niel Verbrigghe | 2022 | Grassland | Root exudates | Monosaccharide |
| 59 | D.D. Kok | 2022 | Cropland | Plant residues | Herbaceous residue |
| 60 | Kazi R. Mehnaz | 2019 | Grassland | Root exudates | Monosaccharide |
|  |  |  |  | Root exudates | Organic acids |
|  |  |  |  | Root exudates | Phenols |
| 61 | Lei Wu | 2019 | Cropland | Plant residues | Crop residue |
| 62 | Zhi Liang | 2023 | Cropland | Plant residues | Crop residue |
| 63 | Shuang Wang | 2024 | Cropland | Root exudates | Monosaccharide |
| 64 | Shuikuan Bei | 2022 | Cropland | Plant residues | Crop residue |
| 65 | Sha Zhou | 2022 | Forest | Root exudates | Monosaccharide |
| 66 | Mouliang Xiao | 2021 | Cropland | Root exudates | Monosaccharide |
|  |  |  |  | Plant residues | Crop residue |
| 67 | Tiangang Tang | 2024 | Forest | Root exudates | Monosaccharide |
| 68 | Zhenke Zhu | 2021 | Cropland | Root exudates | Monosaccharide |
| 69 | Jing Zhou | 2023 | Forest | Root exudates | Monosaccharide |
| 70 | Xiaohan Dong | 2024 | Forest | Root exudates | Monosaccharide |
| 71 | Ndzana Georges Martial | 2023 | Cropland | Root exudates | Organic acids |
| 72 | Chaoran Sun | 2023 | Cropland | Plant residues | Crop residue |
|  |  |  |  | Biochar | Crop biochar |
| 73 | M. You | 2019 | Cropland | Root exudates | Monosaccharide |
|  |  |  | Grassland | Root exudates | Organic acids |
|  |  |  | Cropland | Root exudates | Monosaccharide |
|  |  |  | Grassland | Root exudates | Organic acids |
| 74 | Qinghui Yu | 2023 | Forest | Root exudates | Monosaccharide |
|  |  |  | Grassland |  |  |
| 75 | Junlan Wei | 2022 | Forest | Plant residues | Woody residue |
| 76 | Lin Chao | 2018 | Cropland | Plant residues | Woody residue |
| 77 | Xiao Wang | 2023 | Cropland | Biochar | Crop biochar |
| 78 | Yongxiang Yu | 2024 | Cropland | Microplastics | Polylactic acid |
| 79 | Congli Xiao | 2024 | Cropland | Microplastics | Polylactic acid |
| 80 | Peixuan Cai | 2024 | Cropland | Plant residues | Crop residue |
| 81 | Clayton R. Butterly | 2019 | Cropland | Plant residues | Crop residue |
| 82 | Peng Tian | 2019 | Forest | Root exudates | Monosaccharide |
|  |  |  |  | Root exudates | Polysaccharide |
| 83 | Binaya Parajuli | 2022 | Cropland | Plant residues | Crop residue |
| 84 | Qingyan Qiu | 2023 | Forest | Root exudates | Monosaccharide |
|  |  |  |  | Root exudates | Polysaccharide |
| 85 | Peng Tian | 2019 | Forest | Root exudates | Monosaccharide |
| 86 | Xiuwei Zhang | 2023 | Cropland | Plant residues | Crop residue |
| 87 | Zhiyuan Zhang | 2024 | Forest | Root exudates | Monosaccharide |
|  |  |  |  | Root exudates | Organic acids |
| 88 | Yingde Xu | 2019 | Cropland | Plant residues | Crop residue |
| 89 | Gokul Gaudel | 2024 | Cropland | Root exudates | Monosaccharide |
|  |  |  | Forest |  |  |
|  |  |  | Grassland |  |  |
| 90 | T. J. Purakayastha | 2024 | Cropland | Biochar | Crop biochar |
| 91 | Subin Kalu | 2024 | Cropland | Root exudates | Monosaccharide |
| 92 | Fernanda Santos | 2021 | Forest | Plant residues | Woody residue |
|  |  |  |  | Biochar | Woody biochar |
| 93 | Yasuo Iimura | 2020 | Forest | Root exudates | Monosaccharide |
| 94 | Li Yue | 2019 | Grassland | Root exudates | Monosaccharide |
| 95 | Dongmei Wu | 2024 | Cropland | Plant residues | Woody residue |
| 96 | Rui Zhang | 2024 | Forest | Root exudates | Monosaccharide |
| 97 | Shaobo Yang | 2024 | Forest | Root exudates | Monosaccharide |
| 98 | Shaobo Yang | 2024 | Forest | Root exudates | Monosaccharide |
| 99 | Yuxin Huo | 2024 | Cropland | Microplastics | Polybutylene adipate-co-terephthalate |
| 100 | Qiong Xiao | 2024 | Cropland | Root exudates | Monosaccharide |
| 101 | Kaitao Jia | 2024 | Cropland | Microplastics | Polylactic acid |
| 102 | Guohao Zhang | 2023 | Cropland | Microplastics | Polyhydroxyalkanoates |
|  |  |  |  |  | Polybutylene succinate |
|  |  |  |  |  | Polylactic acid |
| 103 | Liying Chen | 2025 | Cropland | Microplastics | Polylactic acid |
| 104 | Blanca Bernal | 2016 | Forest | Plant residues | Woody residue |
|  |  |  |  | Root exudates | Monosaccharide |
|  |  |  |  | Root exudates | Organic acids |
| 105 | Bassem Dimassi | 2014 | Cropland | Root exudates | Polysaccharide |
| 106 | Hui Wang | 2015 | Forest | Plant residues | Woody residue |
| 107 | Nele Meyer | 2018 | Forest | Root exudates | Monosaccharide |
| 108 | Qingkui Wang | 2014 | Forest | Plant residues | Woody residue |
| 109 | Veronika Jílková | 2021 | Forest | Root exudates | Polysaccharide |
| 110 | Zheng-Rong Kan | 2020 | Cropland | Plant residues | Crop residue |
| 111 | Youchao Chen | 2022 | Cropland | Root exudates | Monosaccharide |
|  |  |  | Cropland | Plant residues | Crop residue |
|  |  |  | Forest | Root exudates | Monosaccharide |
|  |  |  | Forest | Plant residues | Crop residue |
| 112 | Shiting Li | 2024 | Forest | Plant residues | Woody residue |
| 113 | Qiong Liu | 2024 | Cropland | Root exudates | Monosaccharide |
| 114 | Shengxian Chen | 2024 | Cropland | Root exudates | Monosaccharide |
| 115 | Wenkuan Qin | 2024 | Grassland | Root exudates | Monosaccharide |
| 116 | Jiashu Zhou | 2024 | Forest | Plant residues | Crop residue |
|  |  |  |  | Biochar | Crop biochar |
| 117 | Jie Zhou | 2024 | Cropland | Root exudates | Monosaccharide |
| 118 | Weiwei Lu | 2015 | Cropland | Biochar | Crop biochar |
| 119 | Xuejuan Bai | 2024 | Forest | Plant residues | Woody residue |
| 120 | Yongchun Zhou | 2024 | Cropland | Plant residues | Woody residue |
| 121 | Qing Chang | 2024 | Forest | Root exudates | Monosaccharide |
| 122 | Jun Cui | 2024 | Cropland | Plant residues | Crop residue |
| 123 | Vusal Guliyev | 2023 | Cropland | Microplastics | Polybutylene succinate |
| 124 | Lor`ene Siegwart | 2023 | Cropland | Root exudates | Monosaccharide |
| 125 | Jiao Su | 2022 | Grassland | Root exudates | Monosaccharide |
|  |  |  |  | Plant residues | Crop residue |
| 126 | Jing Wang | 2023 | Forest | Root exudates | Monosaccharide |
| 127 | Yuxuan Zhang | 2024 | Cropland | Plant residues | Crop residue |
| 128 | Tiantian Ma | 2024 | Forest | Root exudates | Monosaccharide |
| 129 | Z. Yu | 2018 | Cropland | Biochar | Crop biochar |
| 130 | Yuxue Liu | 2018 | Cropland | Biochar | Crop biochar |
| 131 | Shaobo Yang | 2024 | Forest | Root exudates | Monosaccharide |
| 132 | Elinor F. Boos | 2023 | Cropland | Root exudates | Monosaccharide |
| 133 | Julian Heitkötter | 2017 | Forest | Root exudates | Organic acids |
| 134 | Julian Heitkötter | 2017 | Forest | Root exudates | Organic acids |
| 135 | Michael Herre | 2022 | Forest | Root exudates | Monosaccharide |
|  |  |  |  | Root exudates | Organic acids |
| 136 | Duyen T.T. Hoang | 2017 | Cropland | Root exudates | Monosaccharide |
| 137 | Juan Jia | 2017 | Grassland | Root exudates | Monosaccharide |
| 138 | Kristiina Karhu | 2016 | Forest | Root exudates | Monosaccharide |
| 139 | Chang Liao | 2023 | Cropland | Root exudates | Monosaccharide |
| 140 | Maokui Lü | 2015 | Forest | Root exudates | Monosaccharide |
| 141 | Elaine Pegoraro | 2019 | Grassland | Root exudates | Polysaccharide |
| 142 | Gaoyang Qiu | 2020 | Forest | Root exudates | Monosaccharide |
|  |  |  |  | Plant residues | Crop residue |
| 143 | F. Rukshana | 2013 | Cropland | Root exudates | Monosaccharide |
|  |  |  |  | Root exudates | Organic acids |
| 144 | Tanvir Shahzad | 2019 | Cropland | Plant residues | Crop residue |
| 145 | Juliane Struecker | 2017 | Cropland | Plant residues | Crop residue |
| 146 | Qiuxiang Tian | 2016 | Forest | Root exudates | Monosaccharide |
| 147 | Josefine Walz | 2017 | Grassland | Plant residues | Herbaceous residue |
| 148 | BirgitWild | 2016 | Grassland | Root exudates | Polysaccharide |
|  |  |  |  | Root exudates | Organic acids |
| 149 | Birgit Wild | 2014 | Grassland | Root exudates | Monosaccharide |
|  |  |  |  | Root exudates | Polysaccharide |
|  |  |  |  | Root exudates | Organic acids |
| 150 | Min Zhang | 2021 | Forest | Plant residues | Wood residue |
| 151 | Muhammad Auwal | 2021 | Cropland | Plant residues | Crop residue |
| 152 | Nang Seng Aye | 2018 | Cropland | Root exudates | Monosaccharide |
|  |  |  |  | Root exudates | Polysaccharide |
| 153 | E.V. Blagodatskaya | 2007 | Cropland | Root exudates | Monosaccharide |
| 154 | Ruirui Chen | 2014 | Cropland | Root exudates | Polysaccharide |
|  |  |  |  | Plant residues | Crop residue |
| 155 | Saikat Chowdhury | 2014 | Cropland | Root exudates | Organic acids |
| 156 | Leiyi Chen | 2018 | Grassland | Root exudates | Monosaccharide |
| 157 | D.P. Di Lonardo | 2018 | Cropland | Plant residues | Crop residue |
|  |  |  |  |  | Herbaceous residue |
|  |  |  |  |  | Woody residue |
|  |  |  | Forest |  | Crop residue |
|  |  |  |  |  | Herbaceous residue |
|  |  |  |  |  | Woody residue |
|  |  |  | Grassland |  | Crop residue |
|  |  |  |  |  | Herbaceous residue |
|  |  |  |  |  | Woody residue |
| 158 | Yunying Fang | 2018 | Cropland | Plant residues | Crop residue |
| 159 | S. Fontaine | 2011 | Cropland | Root exudates | Polysaccharide |
| 160 | Xianheng Fu | 2022 | Cropland | Plant residues | Crop residue |
| 161 | Yuexin Fan | 2020 | Forest | Plant residues | Crop residue |
| 162 | Rapha¨el Gutti`eres | 2021 | Cropland | Plant residues | Crop residue |
| 163 | Gokul Gaudel | 2024 | Forest | Root exudates | Monosaccharide |
| 164 | Bertrand Guenet | 2010 | Cropland | Plant residues | Crop residue |
| 165 | Iain P. Hartley | 2010 | Forest | Root exudates | Monosaccharide |
| 166 | Lettice C. Hicks | 2019 | Forest | Root exudates | Monosaccharide |
|  |  |  |  |  | Polysaccharide |
| 167 | Xinyu Jiang | 2016 | Grassland | Biochar | Herbaceous biochar |
| 168 | Eva Kaštovská | 2010 | Cropland | Root exudates | Monosaccharide |
| 169 | LuJun Li | 2018 | Cropland | Plant residues | Herbaceous residue |
|  |  |  | Grassland |  |  |
| 170 | Qianru Li | 2017 | Forest | Root exudates | Monosaccharide |
| 171 | Weiwei Lu | 2014 | Cropland | Biochar | Crop biochar |
| 172 | Bernardo Maestrini | 2014 | Forest | Biochar | Herbaceous biochar |
| 173 | Fanqiao Meng | 2017 | Cropland | Plant residues | Crop residue |
| 174 | Nele Meyer | 2017 | Cropland | Root exudates | Polysaccharide |
| 175 | K.Z. Mganga | 2018 | Cropland | Root exudates | Monosaccharide |
|  |  |  | Forest |  |  |
|  |  |  | Grassland |  |  |
| 176 | Qingyan Qiu | 2015 | Cropland | Plant residues | Crop residue |
| 177 | Qingyan Qiu | 2016 | Cropland | Plant residues | Crop residue |
| 178 | Jennifer L. Soong | 2018 | Forest | Plant residues | Crop residue |
| 179 | Zhuoxia Su | 2023 | Forest | Root exudates | Monosaccharide |
| 180 | Dongdong Wang | 2019 | Cropland | Plant residues | Crop residue |
| 181 | Hui Wang | 2018 | Cropland | Plant residues | Crop residue |
| 182 | Qingkui Wang | 2014 | Forest | Plant residues | Woody residue |
| 183 | Xiaohong Wang | 2021 | Cropland | Plant residues | Crop residue |
| 184 | Shaobo Zhang | 2023 | Forest | Biochar | Crop biochar |
| 185 | Yunyun Zheng | 2022 | Forest | Root exudates | Monosaccharide |
| 186 | Zhenke Zhu | 2018 | Cropland | Plant residues | Crop residue |
| 187 | Peng He | 2024 | Cropland | Plant residues | Crop residue |
| 188 | Ahmad Khan | 2023 | Cropland | Root exudates | Monosaccharide |
| 189 | Waqar Ahmad | 2014 | Cropland | Plant residues | Crop residue |
| 190 | Marie Börger | 2022 | Cropland | Plant residues | Crop residue |
|  |  |  |  | Plant residues | Herbaceous residue |
| 191 | Xi Chen | 2022 | Cropland | Plant residues | Crop residue |
|  |  |  |  | Biochar | Crop biochar |
| 192 | Courtney A. Creamer | 2015 | Cropland | Plant residues | Woody residue |
| 193 | Jun Cui | 2022 | Cropland | Plant residues | Crop residue |
|  |  |  |  | Biochar | Crop biochar |
| 194 | Yunying Fang | 2015 | Cropland | Biochar | Woody biochar |
| 195 | Yunying Fang | 2017 | Grassland | Biochar | Woody biochar |
| 196 | Chenxin Feng | 2021 | Forest | Root exudates | Monosaccharide |
| 197 | R.B. Frøseth | 2015 | Cropland | Plant residues | Herbaceous residue |
| 198 | Lina Gogoi | 2020 | Forest | Biochar | Woody biochar |
| 199 | Francesca M. Hopkins | 2014 | Forest | Root exudates | Monosaccharide |
| 200 | Lei Jiang | 2022 | Wetland | Root exudates | Monosaccharide |
| 201 | Sangeeta Lenka | 2019 | Cropland | Plant residues | Crop residue |
|  |  |  | Wetland |  |  |
| 202 | Qianyuan Liu | 2019 | Forest | Root exudates | Monosaccharide |
| 203 | Jianwei Li | 2018 | Forest | Plant residues | Woody residue |
| 204 | Huan Li | 2022 | Forest | Root exudates | Monosaccharide |
| 205 | Qingkui Wang | 2016 | Forest | Plant residues | Woody residue |
| 206 | Mengyang You | 2021 | Cropland | Plant residues | Crop residue |
| 207 | Dan Zhang | 2021 | Wetland | Root exudates | Monosaccharide |
| 208 | Hui Wang | 2014 | Forest | Plant residues | Crop residue |
| 209 | Yakov Kuzyakov | 2006 | Grassland | Root exudates | Polysaccharide |
| 210 | Bertrand Guenet | 2012 | Cropland | Root exudates | Polysaccharide |
|  |  |  |  | Plant residues | Crop residue |
| 211 | Rongzhong Ye | 2015 | Cropland | Plant residues | Crop residue |
| 212 | Yu Luo | 2017 | Cropland | Root exudates | Monosaccharide |
| 213 | Muhammad Shahbaz | 2017 | Cropland | Plant residues | Crop residue |
| 214 | Brianna K. Finley | 2018 | Forest | Plant residues | Woody residue |
| 215 | L. Falchini | 2003 | Grassland | Root exudates | Monosaccharide |
|  |  |  |  | Root exudates | Organic acids |
| 216 | Ute Hamer | 2005 | Cropland | Root exudates | Polysaccharide |
|  |  |  |  | Root exudates | Organic acids |
|  |  |  |  | Root exudates | Polysaccharide |
|  |  |  |  | Root exudates | Organic acids |
|  |  |  |  | Root exudates | Phenols |
| 217 | Sergey Blagodatsky | 2010 | Cropland | Root exudates | Monosaccharide |
| 218 | E. Blagodatskaya | 2011 | Cropland | Root exudates | Monosaccharide |
| 219 | Andrew Cross | 2011 | Forest | Biochar | Woody biochar |
|  |  |  | Wetland |  |  |
| 220 | Weidong Zhang | 2012 | Forest | Plant residues | Woody residue |
| 221 | Fernanda Santos | 2012 | Forest | Plant residues | Woody residue |
|  |  |  |  | Biochar | Woody biochar |
| 222 | Felipe Bastida | 2013 | Wetland | Root exudates | Monosaccharide |
| 223 | Evgenia Blagodatskaya | 2014 | Grassland | Root exudates | Polysaccharide |
| 224 | Yueling Zhang | 2015 | Cropland | Root exudates | Monosaccharide |
| 225 | Mette Vestergård | 2016 | Grassland | Root exudates | Monosaccharide |
| 226 | Mandeep Singh | 2017 | Cropland | Root exudates | Organic acids |
| 227 | Jun Cui | 2017 | Cropland | Plant residues | Crop residue |
|  |  |  |  | Biochar | Crop biochar |
| 228 | Kyle Mason-Jones | 2017 | Cropland | Root exudates | Monosaccharide |
| 229 | Tengxiang Lian | 2017 | Cropland | Plant residues | Crop residue |
| 230 | Muhammad Shahbaz | 2018 | Cropland | Plant residues | Crop residue |
| 231 | J. L. CHOTTE | 1998 | Cropland | Root exudates | Monosaccharide |
|  |  |  |  | Root exudates | Polysaccharide |
|  |  |  |  | Plant residues | Crop residue |
| 232 | Marie Sauvadet | 2018 | Cropland | Plant residues | Crop residue |
| 233 | J. Magid | 1999 | Cropland | Plant residues | Herbaceous residue |
| 234 | Andrew T. Nottingham | 2009 | Forest | Root exudates | Monosaccharide |
|  |  |  |  | Plant residues | Crop residue |
| 235 | Weidong Zhang | 2014 | Forest | Plant residues | Woody residue |
| 236 | Chen Lin | 2014 | Cropland | Plant residues | Crop residue |
| 237 | Thorald Eck | 2015 | Cropland | Plant residues | Crop residue |
| 238 | Huanjun Zhang | 2016 | Cropland | Root exudates | Monosaccharide |
| 239 | Xiao Liang | 2017 | Cropland | Plant residues | Crop residue |
| 240 | J. WU | 1993 | Grassland | Root exudates | Monosaccharide |
|  |  |  |  | Plant residues | Herbaceous residue |
| 241 | Oliver Dilly | 2014 | Forest | Root exudates | Monosaccharide |
| 242 | M.L. Luna-Guido | 2003 | Wetland | Plant residues | Crop residue |
| 243 | Xiuwei Zhang | 2017 | Cropland | Plant residues | Crop residue |
| 244 | YIN Yun-feng | 2014 | Cropland | Plant residues | Crop residue |
| 245 | B. Guenet | 2010 | Cropland | Root exudates | Polysaccharide |
| 246 | H. M. S. K. Herath | 2015 | Grassland | Plant residues | Crop residue |
|  |  |  |  | Biochar | Crop biochar |
| 247 | Balal Yousaf | 2017 | Cropland | Biochar | Woody biochar |
| 248 | Na Qiao | 2014 | Forest | Root exudates | Monosaccharide |
| 249 | Lening Hu | 2011 | Cropland | Plant residues | Crop residue |
| 250 | Jinshui Wu | 2011 | Cropland | Root exudates | Monosaccharide |
|  |  |  |  | Plant residues | Crop residue |
| 251 | R. Bol | 2003 | Grassland | Plant residues | Crop residue |
| 252 | Se´bastien Fontaine | 2004 | Grassland | Root exudates | Polysaccharide |
| 253 | Nang Seng Aye | 2016 | Cropland | Plant residues | Crop residue |
| 254 | Muhammad Shahbaz | 2016 | Cropland | Plant residues | Crop residue |
| 255 | Maokui Lyu | 2019 | Forest | Plant residues | Woody residue |
| 256 | Noe´mie Pascault | 2013 | Cropland | Plant residues | Crop residue |
| 257 | Qingkui Wang | 2015 | Forest | Plant residues | Woody residue |
| 258 | Pascal Lienhard | 2013 | Cropland | Plant residues | Crop residue |
|  |  |  | Grassland |  |  |
| 259 | Shujie Miao | 2016 | Cropland | Plant residues | Crop residue |
| 260 | Raquel Schmatz | 2016 | Cropland | Plant residues | Crop residue |
| 261 | Christophe Naisse | 2014 | Grassland | Plant residues | Herbaceous residue |
|  |  |  | Forest | Plant residues | Herbaceous residue |
|  |  |  | Grassland | Biochar | Herbaceous biochar |
|  |  |  | Forest | Biochar | Herbaceous biochar |
| 262 | Peng Su | 2015 | Cropland | Plant residues | Woody residue |
| 263 | Xianheng Fu | 2025 | Cropland | Plant residues | Crop residue |
| 264 | Julia Wiesenbauer | 2025 | Forest | Root exudates | Polysaccharide |
|  |  |  | Forest | Root exudates | Organic acids |
|  |  |  | Grassland | Root exudates | Polysaccharide |
|  |  |  | Grassland | Root exudates | Organic acids |
| 265 | Junjie Lin | 2025 | Cropland | Microplastics | Polyhydroxyalkanoates |
|  |  |  |  |  | Polylactic acid |
| 266 | Shuang Zhou | 2021 | Cropland | Root exudates | Monosaccharide |
|  |  |  |  | Root exudates | Organic acids |
| 267 | Mengyang You | 2025 | Forest | Root exudates | Monosaccharide |
|  |  |  | Grassland | Root exudates | Monosaccharide |
| 268 | Xianheng Fu | 2025 | Cropland | Plant residues | Crop residue |
| 269 | Mengmeng Chen | 2025 | Cropland | Plant residues | Crop residue |
| 270 | Hui Wang | 2025 | Cropland | Plant residues | Crop residue |
| 271 | Hongxin Dong | 2025 | Cropland | Microplastics | Polyhydroxyalkanoates |
|  |  |  |  |  | Polylactic acid |
| 272 | Zhenhui Jiang | 2025 | Forest | Plant residues | Crop residue |
|  |  |  |  | Biochar | Crop biochar |
| 273 | Qingyan Qiu | 2025 | Forest | Plant residues | Woody residue |
| 274 | Rui Wang | 2025 | Cropland | Biochar | Woody biochar |
| 275 | Qiong Xiao | 2025 | Cropland | Root exudates | Monosaccharide |
| 276 | Qingkui Wang | 2025 | Forest | Root exudates | Monosaccharide |
| 277 | Takuma Koyama | 2025 | Cropland | Root exudates | Monosaccharide |
|  |  |  |  |  | Organic acids |
| 278 | Xinyu Bai | 2025 | Forest | Root exudates | Monosaccharide |
| 279 | Jiachen Pan | 2025 | Cropland | Root exudates | Monosaccharide |
| 280 | Xiuwei Zhang | 2025 | Cropland | Root exudates | Monosaccharide |
| 281 | Xinli Chen | 2025 | Forest | Root exudates | Monosaccharide |
|  |  |  | Cropland |  |  |
| 282 | Jie Yu | 2025 | Cropland | Plant residues | Crop residue |
| 283 | Xinyu Hou | 2025 | Forest | Root exudates | Monosaccharide |
|  |  |  |  |  | Organic acids |

**Table S2** The plant residues, root exudates, biochar and microplastics considered for the effects on soil organic carbon (SOM) priming, **s**ample size (number of observations), results of testing publication bias and random-effect models for each response variable. Publication bias was tested through fail-safe number and trim and fill models.

|  | n | Fail-safe number | Original model  Effect size and 95% CIs | Trim and fill model  Effect size and 95% CIs |
| --- | --- | --- | --- | --- |
| Plant residues | 3423 | 212459258 | 0.5508 (0.5157/0.5845) | 0.8576 (0.8239/0.8913) |
| Root exudates | 3464 | 192566628 | 0.4714 (0.4433/0.4995) | 0.7139 (0.6863/0.7415) |
| Biochar | 846 | 6406764 | 0.3748 (0.3222/0.4273) | 0.3748 (0.3222/0.4273) |
| Microplastics | 282 | 512448 | 0.3419 (0.2562/0.4275) | 0.3419 (0.2562/0.4275) |

**Data S1** References in Table S1. The studies in Chinese but with English abstract are marked with blue.

1. Chen, T., Yuan, F.H., Zhang, L.M., Hu, C.L., 2022. Effects of addition of leaf litter with different chemical properties on soil organic carbon mineralization and priming effect．Chinese Journal of Applied Ecology 33(10), 2602-2610.
2. Yuan, F., Ying, Y., Chen, Z., Lou, X., Hu, Y., 2024. Effects of Chinese fir leaf litter addition on soil CO_2_ release and priming effect under different incubation temperatures. Chinese Journal of Ecology 43(4), 993-999.
3. Tang, A., Xiao, Y., Xian, X., Jin, R., Long. S., Chen, Yulan., Li, B., Wang, C.Q., 2023. Effects of different biochars application on mineralization characteristics of organic carbon in acidic purple soil. Journal of Sichuan Agricultural University 41(1), 111-118.
4. Tao, Y., Liu, X., Zhao, X.C., Wang, Q.K., 2025. Effect of different exogenous carbon inputs on soil organic carbon decomposition and priming effect in two Chinese fir forests. Acta Ecologica Sinica 45(6), 1-12.
5. Lou, X., Chen, Z., Ying, Y., Hu, Y., 2024. Effects of herbivory leaf litter addition on soil organic carbon mineralization and priming effect. Journal of Forest and Environment 44(5), 492-500.
6. Xu, M., Liu, Y.Y., Yuan, X.C., Zeng, Q.X., Lin, H.Y., Wu, X.X., Cui, J.Y., Chen, W.W., Chen, Y.M., 2022. Effects of different carbon addition modes on the soil priming effect of a subtropical Phyllostachys edulis forest under nitrogen deposition. The Journal of Applied Ecology 33(10), 2619-2627.
7. Mei, K., Chen, Y., Fan, Y., Zhou, J., Zhang, Q., Cheng, L., Zeng, Q., Xu, J., Yuan, X., Cui, J., Liu, Y., 2022. Effects of litters and phosphorus addition on soil carbon priming effect in *Pinus massoniana* forest. Acta Pedologica Sinica 59(4), 1089-1099.
8. Dai, J.Z., Wei, Z.J., He, N.P., W, R.M., W, X.H., Zhang, Y.H., Zhao, X.N., Yu, G.R., 2012. Effect of grazing enclosure on the priming effect and temperature sensitivity of soil C miner alization in Leymus chinensis grasslands, Inner Mongolia, China. Chinese Journal of Plant Ecology 36(12), 1226-1236.
9. Sun, Z.A., Wang, F., Zhang, S., Zhang, X., Meng, F.Q., 2021. The effect of straw addition on organic and inorganic carbon release from calcareous soils. Acta Pedologica Sinica 58(6), 1520-1529.
10. Chao, L., Ou, M.F., Chen, J., Lu, W.F., Ma, L., Li, Z.G., Huang, B.H., Ming, A.G., Zhang, J.B., Hu, B.Q., Liu, Y.Y., 2024. Leaf litter decomposition and its priming effect of eleven typical ecological restoration tree species in the karst area of china. Acta Ecologica Sinica 44(8), 3397-3407.
11. Tao, Y., Liu, X., Zhao, X., Wang, Q., 2025. Effects of different exogenous carbon inputs on the soil organic carbon decomposition and priming effect in two Chinese fir forests. Acta Ecologica Sinica 45(6), 2698-2709.
12. Zhang, Z., Cai, X.Z., Tang, C.D., Guo, J.F., 2017. Priming effect of dissolved organic matter in the surface soil of a *Cunninghamia* lanceolata plantation．Acta Ecologica Sinica 37(22), 7660-7667．
13. Tang, G.M., Xu, W.L., Gu, M.Y., Yao, H.Y., Pu, S.H., Hu, K.L., 2019. Characteristics of cotton stalk-char and its effect on organic carbon mineralization in grey desert soil. Acta Ecologica Sinica 39(5), 1795-1803.
14. Li, Y., Wang, C.M., Gao, S.J., Wang, P., Qiu, J.Z., Shang, S.S., 2021.Effects of coupling effects of nitrogen addition and simulated warming on soil carbon priming effect in alpine peat wealand. Environmental Chemistry 40(8), 2430-2438.
15. Wang, X.F., Wang, S.L., Zhang, W.D., 2013. Effects of Chinese fir litter on soil organic carbon decomposition and microbial biomass carbon. Chinese Journal of Applied Ecology 24(9), 2393-2398.
16. Lu, X.R., Yin. Y., Feng, J.X., Ma, H.L., Gao, R., Yin, Y.F., 2020. Effects of chinese fir litter and its biochar addition on mineralization of native soil organic carbon. Acta Pedologica Sinica 57(4), 943-953.
17. Wang, L., Zhao X.C., Yang, S.B., Wang, Q.K., 2024. Priming effect of soil organic carbon decomposition induced by Cunninghamia lanceolate leaf litter and fine root and its response to nitrogen addition in subtropical forests. Chinese Journal of Plant Ecology, 48(11), 1434-1444.
18. Liu, B.J., Xie, Z.B., Liu, Q., Wang, X.J., Lin, Z.B., Bei, Q.C., Lin, X.W., Liu, G., Zhu, J.G., 2021. Correlation between biochar-induced carbon priming effect in soils and soil physiochemical properties. Soils 53(2), 343-353.
19. Li, M.L., Xu, M.X., Chen, Y.S., Ye, L.L., Jiang, J.P., 2022. Effects of different amounts of calcium carbonate on the mineralization of straw organic carbon in calcareous soil. Ecology and Environmental Sciences 31(10), 2002-2009.
20. Q, Y.F., Wang, X.Y., Tang Y.J., Miao, S.J., 2024. Responses of priming effect of soil organic carbon to carbon component of rice straw addition. Journal of Agro-Environment Science 43(10), 2416-2423.
21. Li, J.Y., Lyu, M.K., Li, X.J., Jiang, Y.M., Xie, J. S., 2021. Effects of soil moisture on priming effect of soil organic carbon in meadow in Wuyi Mountain,China．Chinese Journal of Applied Ecology 32(4), 1250-1258.
22. Huang, X.M., Chen, L.C., Tian, N., Guan, X., Hu, Y.L., Huang K., Su, X.J., Tao, X., 2023. Effect of leaves damaged by Dendrolimus punctatus and insect frass on soil priming effect. Chinese Journal of Applied Ecology 34(3), 770-776.
23. Luo, Y., Zhao, X.R., Li, G.T., Zhao, L.X., Meng, H.B., Lin, Q.M., 2014. Characteristics of difference in priming effect of miscanthus-derived biochar in acid and alkaline soils. Acta Pedologica Sinica 51(01), 90-95.
24. Hu, L.N., Su, Y.R., He, X.Y., Li, Y., Wang, A.H., Wu, J.S., 2011. Effect to the turnover of soil organic carbon in three typical soils in karst area by adding ^14^C-labelled organic matter. Journal of Agro-Environment Science 30(11), 2368-2373.
25. Leng X.M., Qian J.S., Zhan X.H., Xie W.Y., Liu X.Y., Zheng J.D., Guo S.W., Li L.Q., Pan G.X., 2022. Effects of external organic matter input on the mineralization of organic carbon in paddy soils with long-term fertilization. Journal of Nanjing Agicultural University 45(1), 103-112.
26. Miao, S.J., Qiao, Y.F., Wang, W.T., Shi, Y.H., 2019. Priming effect of maize straw addition on soil organic matter in yellow-brown soil. Soils 51(3), 622-626.
27. Zhang, Y.T., Xu, W.H., Wang, Q.K., Tian, P., 2025. Effects of iron oxides on the priming effect of topsoil and subsoil carbon decomposition in a subtropical forest. Acta Pedologica Sinica 62(02), 504-516.
28. Wang, Y.Q., Li, Y.H., Zhu, Z.K., Wu, J.S., Ge, T.D., 2022. The effect of iron oxides on mineralization, transformation and priming effect of acetate in anoxic paddy soils. Acta Pedologica Sinica 59(6), 1683-1694.
29. Lu, D.Q., Zhang, S.L., Yang, X.Y., 2007. Effect of supplying C and N on the mineralization and priming effect of organic matter in loessial soil. Plant Nutrition and Fertilizer Science 13(3), 423-429.
30. Li, N., Teng, P.J., Lei, W.Y., Long, J.H., Li, L.J., 2023. Effects and mechanisms of addition of different types of exogenous organic materials on priming effect of organic carbon in arable black soils. Chinese Journal of Eco-Agriculture 31(10), 1588-1601.
31. Chen, L.X., Li, G., Liu, Y.C., Duan, W.B., Sun, S.H., Li, F.F., Li, S.B., Mao, H.Y., 2017. Priming effect of coupling function of exogenous organic matter and temperature on soil organic carbon of pinus koraiensis broad-leaved mixed forest. Forest Research 30(5), 797-804.
32. Wang, R.M., Dong, K.H., He, N.P., Zhu, J.X., Dai, J.Z., Shi, K.K., 2013. Effect of enclosure on soil C mineralization and priming effect in Stipa grandis grassland of Inner Mongolia. Acta Ecologica Sinica 33(12), 3622-3629.
33. Duan, J.J., Luo, A.H., LI, R.D., Chen, L., Chen, J., Wang, X.L., Gao, Z.L., 2022. Temperature remarkedly affecting organic carbon mineralization of karst yellow rendzina, priming effect of rice straw, and Q_10_ in Guizhou province. Journal of Soil and Water Conservation 36(05), 265-280.
34. Gan, Z.Y., Wang, H., Ding, C., Lei, M., Yang, X.G., Cai, J.Y., Qiu, Q.Y., Hu, Y.L., 2022. Effects of dissolved organic matter derived from different plant and tissues in a subtropical forest on soil priming effect and the underlying mechanisms. Chinese Journal of Plant Ecology 46(7), 797-810.
35. Tang, Z.Z., Zhu, Z.K., Shen, B.J., Hu, Y.J., Wang, J., Pang, J., Tong, C.L., Ge, T.D., Wu, J.S., 2017. Effect of stoichiometric ratio of soil nutrients on mineralization and priming effect of glucose in paddy soil. Acta Pedologica Sinica 54(01), 246-254.
36. Qiu, Q.Y., Yao, K.L., Liu, J., Ge, Z.Q., Xu, W.B., Liu, H.X., Hu, Y.L., 2019. Effects of labile organic carbon input on the priming effect along an ecological restoration gradient. Acta Ecologica Sinica 39(13), 4855-4864．
37. Wang, H., Yang, Y., Xi, D., Qiu, Q.Y., Hu, Y.L., 2020. Impacts of labile organic carbon input on the priming effect of three forest soils in Wuyi Mountain. Acta Ecologica Sinica 40(24), 9184-9194.
38. Li, A.M., Li, H., Pei J.B., Xie, N.K., Liu, Y.W., Wang, J.K., 2019. Effects of maize straw application on organic carbon′s priming effect and temperature sensitivity in brown earth. Journal of Agro-Environment Science 38(12), 2788-2796.
39. Liu, F., Wang, Y.Q., Zhang, Y., Zhu, Z.K., Wu, J.S., Ge, T.D., Li, Y.D., 2022. Effect of long-term straw returning on the mineralization and priming effect of rice root-carbon. Environmental Science 43(08), 4372-4378.
40. Jiang, J.B., Zhu, Z.K., Lin, S., Li, Y.H., Li, K.L., Wang, X.L., Ge, T.D., Wu, J.S., 2021. Mineralization of goethite-adsorbed and -encapsulated organic carbon and its priming effect in paddy soil. Acta Pedologica Sinica 58(6), 1530-1539.
41. Chen, J.R., Wei, X.H., Hu, J.M., Shi, Y.Z., Zhang, J.H., Zheng, F.H., 2024. Effect of biochar-based fertilizer on temperature sensitivity of soil organic carbon mineralization in aged tea gardens. Chinese Journal of Agrometeorology 45(03), 245-256.
42. Wei, Y.Y., Cui, L.J., Zhang, M.Y., Liu, W.W., Wang, D.A., Yang, S., Xiao, H.Y., 2019. Effects of exogenous carbon input on soil or ganic carbon mineralization and temperature sensitivity of cropland and wetland in the North China Plain. Chinese Journal of Eco-Agriculture 27(10), 1463-1471.
43. Huang, S.S., Huo, C.F., Xie H.T., Wang, P., Cheng, W.X., 2019. Soil organic carbon mineralization and priming effects in the topsoil and subsoil under no-tillage black soil. Chinese Journal of Applied Ecology 30(6), 1877-1884.
44. Xue, J.Y., Zhang, H.X., Quan, Q., Wang, R.M., Gan, Y.M., He, N.P., 2014. Effect of land-use type on soil carbon mineralization and its priming effect on red soils in the mid-subtropics of China. Chinese Journal of Applied and Environmental Biology 20 (3), 516-522.
45. Liao, C., Tian, Q.X., Wang, D.Y., Qu, L.Y., Wu, Y., Liu, F., 2016. Effects of labile carbon addition on organic carbon mineralization and microbial growth strategies in subtropical forest soils. Chinese Journal of Applied Ecology 27(9), 2848-2854.
46. Yuan, S.F., Wang, S.L., Zhang, W.D., 2015. Effect of external organic carbon and temperature on SOC decomposition. Chinese Journal of Soil Science 46(4), 916-922.
47. Zhu, Y.F., Sun, Z.L., Wang, Q.K., 2020. Effects of biochar and nitrogen additions on soil organic carbon decomposition and balance in a subtropical forest. Chinese Journal of Ecology 39(9), 2851-2859.
48. Zhang, Y., Liu, X., Ren, X.J., Li, D.F., Wu, D.F., Chen, X.L., 2019. Effects of straw and biochar on soil carbon pool management index and organic carbon mineralization. Journal of Soil and Water Conservation 33(03), 153-159.
49. Zhang, S.Q., Wang, C.L., Xu, X.Y., Peng, B.Y., Li, Y., Yang, X.T., Lei, H., Hou, R.D., 2025. Effect of exogenous carbon inputs on soil organic carbon mineralization and its components in the tundra. Acta Ecologica Sinica 45(5), 2122-214050.
50. Wu, H.Y., Wang, J.Q., Xia. F., Wei, W., Wu, M.Q., Wang, W., Ran, L.L., Yan, S., Xue, Y. Y., Zheng, S. K., Shi, H., Wang, J.L., He, J.D., 2025. Effects of exogenous carbon addition on soil organic carbon mineralisation and microbial community structure in alpine meadows of northern Tibet. Pratacultural Science, https://doi.org/10.11829/j.issn.1001-0629.2024-0560.
51. Hu, C.Y., Zhang, Y.R, Han, C., Li, P.G., Han, G.X., Song, W.M., 2025. Impacts of exogenous organic carbon input and soil properties on the priming effect in coastal wetlands of Yellow River Delta. China.Chinese Journal of Ecology, 44(8), 2497-2505.
52. Yang, X., Zhu, J., Ren, T., Li, X.K., Lu, J.W., 2025. The influence of straw treatment methods on straw mineralization and priming effects in soil. Acta Pedologica Sinica, https://doi.org/10.11766/trxb202406290265.
53. Lu, C., Zhang, Y., Sun, A.Q., Han, R., Chen, Q.L., 2025. Warming alters microplastic-induced soil carbon priming across diverse terrestrial ecosystems. Geoderma 460, 117414.
54. Jin, W., Dong, H., Wu, Q., Shao, S., Liang, C., Ma, X., Li, Y., Luo, Y., Chen, J., Qin, H., 2025. Microbial stoichiometric imbalance and community composition determine the priming effects caused by leaf litter input in a subtropical forest soil. Plant and Soil 515, 1499-1516.
55. Fan, F., Yu, B., Wang, B., George, T.S., Yin, H., Xu, D., Li, D., Song, A., 2019. Microbial mechanisms of the contrast residue decomposition and priming effect in soils with different organic and chemical fertilization histories. Soil Biology and Biochemistry 135, 213-221.
56. Zhou, X., Feng, Z., Yao, Y., Liu, R., Shao, J., Jia, S., Gao, Y., Xue, K., Chen, H., Fu, Y., He, Y., 2025. Nitrogen input alleviates the priming effects of biochar addition on soil organic carbon decomposition. Soil Biology and Biochemistry 202, 109689.
57. Lu, W., Zha, Q., Zhang, H., Chen, H.Y.H., Yu, J., Tu, F., Ruan, H., 2021. Changes in soil microbial communities and priming effects induced by rice straw pyrogenic organic matter produced at two temperatures. Geoderma 400, 115217.
58. Verbrigghe, N., Meeran, K., Bahn, M., Fuchslueger, L., Janssens, I.A., Richter, A., Sigurdsson, B.D., Soong, J.L., Vicca, S., 2022. Negative priming of soil organic matter following long-term in situ warming of sub-arctic soils. Geoderma 410, 115652.
59. Kok, D.D., Scherer, L., De Vries, W., Trimbos, K., Van Bodegom, P.M., 2022. Relationships of priming effects with organic amendment composition and soil microbial properties. Geoderma 422, 115951.
60. Mehnaz, K.R., Corneo, P.E., Keitel, C., Dijkstra, F.A., 2019. Carbon and phosphorus addition effects on microbial carbon use efficiency, soil organic matter priming, gross nitrogen mineralization and nitrous oxide emission from soil. Soil Biology and Biochemistry 134, 175-186.
61. Wu, L., Zhang, W., Wei, W., He, Z., Kuzyakov, Y., Bol, R., Hu, R., 2019. Soil organic matter priming and carbon balance after straw addition is regulated by long-term fertilization. Soil Biology and Biochemistry 135, 383-391.
62. Liang, Z., Rasmussen, J., Poeplau, C., Elsgaard, L., 2023. Priming effects decrease with the quantity of cover crop residues-Potential implications for soil carbon sequestration. Soil Biology and Biochemistry 184, 109110.
63. Wang, S., Gao, W., Ma, Z., Zhu, Z., Luo, Y., Wei, L., Yuan, H., Chen, S., Ying, C., Mason-Jones, K., Kuzyakov, Y., Ge, T., 2024. Iron mineral type controls organic matter stability and priming in paddy soil under anaerobic conditions. Soil Biology and Biochemistry 197, 109518.
64. Bei, S., Li, X., Kuyper, T.W., Chadwick, D.R., Zhang, J., 2022. Nitrogen availability mediates the priming effect of soil organic matter by preferentially altering the straw carbon-assimilating microbial community. Science of the Total Environment 815, 152882.
65. Zhou, S., Wang, Jieying, Chen, L., Wang, Jun, Zhao, F., 2022. Microbial community structure and functional genes drive soil priming effect following afforestation. Science of the Total Environment 825, 153925.
66. Xiao, M., Shahbaz, M., Liang, Y., Yang, J., Wang, S., Chadwicka, D.R., Jones, D., Chen, J., Ge, T., 2021. Effect of microplastics on organic matter decomposition in paddy soil amended with crop residues and labile C: A three-source-partitioning study. Journal of Hazardous Materials 416, 126221.
67. Tang, T., Zhang, Q., Fu, R., Duan, P., Zhang, Y., Yang, X., Wang, K., Li, D., 2024. Lithologic control of priming effect in subtropical forest. Catena 238, 107912.
68. Zhu, Z., Zhou, J., Shahbaz, M., Tang, H., Liu, S., Zhang, W., Yuan, H., Zhou, P., Alharbi, H., Wu, J., Kuzyakov, Y., Ge, T., 2021. Microorganisms maintain C:N stoichiometric balance by regulating the priming effect in long-term fertilized soils. Applied Soil Ecology 167, 104033.
69. Zhou, J., Qiao, N., Zhu, T., Pang, R., Sun, Y., Zhou, X., Xu, X., 2023. Native soil labile organic matter influences soil priming effects. Applied Soil Ecology 182, 104732.
70. Dong, X., Fang, Y., Liu, D., Guan, X., Huang, K., Liu, Y., Wang, Q., Wang, S., Zhang, W., 2024. In-situ warming does not change soil priming effect induced by glucose addition in a temperate forest. Pedosphere 34, 759-769.
71. Georges Martial, N., Yao, S., Hamer, U., Zhang, Y., Zhang, B., 2023. Positive and negative priming effects induced by freshly added mineral-associated oxalic acid in a Mollisol. Rhizosphere 26, 100708.
72. Sun, C., Tan, S., Qiu, Z., Sun, S., Jiang, P., Chen, L., 2023. Effects of different exogenous organic substrates on soil carbon and nitrogen mineralization and their priming effects. Agronomy 13, 3017.
73. You, M., Han, X., Chen, X., Yan, J., Li, N., Zou, W., Lu, X., Li, Y., Horwath, W.R., 2019. Effect of reduction of aggregate size on the priming effect in a Mollisol under different soil managements. European Journal of Soil Science 70, 765–775.
74. Yu, Q., Zhang, Z., He, Y., Hao, M., Wang, G., Dun, X., Wu, Q., Gao, P., 2023. Secondary shrubs promoted the priming effect by increasing soil particle organic carbon mineralization. Frontiers in Forests and Global Change 6, 1288259.
75. Wei, J., Zhang, Q., Wang, Q., Tian, P., 2022. Effects of litter Input on temperature sensitivity of soil organic carbon mineralization along a forest elevation gradient. Forests 13, 1250
76. Chao, L., Liu, Y., Freschet, G.T., Zhang, W., Yu, X., Zheng, W., Guan, X., Yang, Q., Chen, L., Dijkstra, F.A., Wang, S., 2019. Litter carbon and nutrient chemistry control the magnitude of soil priming effect. Functional Ecology 33, 876-888.
77. Wang, X., Li, Z., Cheng, Y., Yao, H., Li, H., You, X., Zhang, C., Li, Y., 2023. Wheat straw hydrochar induced negative priming effect on carbon decomposition in a coastal soil. iMeta 2, e134.
78. Yu, Y., Lin, S., Sarkar, B., Wang, J., Liu, X., Wang, D., Ge, T., Li, Y., Zhu, B., Yao, H., 2024. Mineralization and microbial utilization of poly(lactic acid) microplastic in soil. Journal of Hazardous Materials 476, 135080.
79. Xiao, C., Liu, X., Wang, D., Xue, J., Liu, L., Yu, Y., Yao, H., 2024. Impact of moisture on the degradation and priming effects of poly(lactic acid) microplastic. Land Degradation and Development 35, 4623–4636.
80. Cai, P., Wang, H., Zhao, Z., Li, X., Wang, Y., Zhan, X., Han, X., 2024. Effects of straw addition on soil priming effects under different tillage and straw return modes. Plants 13, 3188.
81. Butterly, C.R., Armstrong, R.D., Chen, D., Tang, C., 2019. Residue decomposition and soil carbon priming in three contrasting soils previously exposed to elevated CO_2_. Biology and Fertility of Soils 55, 17-29.
82. Tian, P., Mason-Jones, K., Liu, S., Wang, Q., Sun, T., 2019. Form of nitrogen deposition affects soil organic matter priming by glucose and cellulose. Biology and Fertility of Soils 55, 383-391.
83. Parajuli, B., Ye, R., Szogi, A., 2022. Mineral N suppressed priming effect while increasing microbial C use efficiency and N_2_O production in sandy soils under long-term conservation management. Biology and Fertility of Soils 58, 903-915.
84. Qiu, Q., Li, M., Mgelwa, A.S., Hu, Y.L., 2023. Divergent mineralization of exogenous organic substrates and their priming effects depending on soil types. Biology and Fertility of Soils 59, 87-101.
85. Tian, P., Liu, S., Wang, Q., Sun, T., Blagodatskaya, E., 2019. Organic N deposition favours soil C sequestration by decreasing priming effect. Plant and Soil 445, 439-451.
86. Zhang, X., Zhu, B., Yu, F.-H., Wang, P., Cheng, W., 2023. Long-term bare fallow soil reveals the temperature sensitivity of priming effect of the relatively stabilized soil organic matter. Plant and Soil 488, 57-70.
87. Zhang, Z., Wang, W., Qi, J., Zhang, H., Tao, F., Zhang, R., 2019. Priming effects of soil organic matter decomposition with addition of different carbon substrates. Journal of Soils and Sediments 19, 1171-1178.
88. Xu, Y., Ding, F., Gao, X., Wang, Y., Li, M., Wang, J., 2019. Mineralization of plant residues and native soil carbon as affected by soil fertility and residue type. Journal of Soils and Sediments 19, 1407-1415.
89. Gaudel, G., Xing, L., Raseduzzaman, M., Poudel, M., Dong, W., Hu, C., 2024. Soil microbes, carbon, nitrogen, and the carbon to nitrogen ratio indicate priming effects across terrestrial ecosystems. Journal of Soils and Sediments 24, 307-322.
90. Purakayastha, T.J., Bera, T., Dey, S., Pande, P., Kumari, S., Bhowmik, A., 2024. Biochar aided priming of carbon and nutrient availability in three soil orders of India. Scientific Reports 14, 8420.
91. Kalu, S., Seppänen, A., Mganga, K.Z., Sietiö, O.-M., Glaser, B., Karhu, K., 2024. Biochar reduced the mineralization of native and added soil organic carbon: evidence of negative priming and enhanced microbial carbon use efficiency. Biochar 6, 7.
92. Santos, F., Rice, D.M., Bird, J.A., Berhe, A.A., 2021. Pyrolysis temperature and soil depth interactions determine PyC turnover and induced soil organic carbon priming. Biogeochemistry 153, 47-65.
93. Iimura, Y., Tanaka, D., Nagao, S., Fujitake, N., Ohtsuka, T., 2020. The mineralization rate of black soil carbon in the deep layers of Japanese volcanic ash soil may be easily accelerated by labile carbon supply. Soil Science and Plant Nutrition 66, 415-420.
94. Li, Y., Nie, C., Shao, R., Du, W., Liu, Y., 2019. Soil priming effect mediated by nitrogen fertilizationg gradients in a Semi-arid gassland, China. Journal of Resources and Ecology 10, 147.
95. Wu, D., Wang, X., Fan, A., Chen, Y., Yang, Z., Lyu, M., Yao, X., Guo, J., Yang, Y., Chen, G., 2024. Consistent positive response but inconsistent microbial mechanisms of absorptive root litter-induced priming effect to warming at different decomposition stages. Geoderma 452, 117079.
96. Zhang, R., Li, R., Kuang, J., Shi, Z., 2024. Influence of drought intensity on soil carbon priming and its temperature sensitivity after rewetting. Science of the Total Environment 908, 168362.
97. Yang, S., Zhao, X., Wang, Q., Tian, P., 2024b. Greater influences of nitrogen addition on priming effect in forest subsoil than topsoil regardless of incubation warming. Science of the Total Environment 946, 174308.
98. Yang, S., Zhao, X., Sun, Z., Wang, L., Tian, P., Wang, Q., 2024a. Carbon and nitrogen addition-derived enzyme activities in topsoil but nitrogen availability in subsoil controls the response of soil organic carbon decomposition to warming. Science of the Total Environment 949, 175261.
99. Huo, Y., Dijkstra, F.A., Possell, M., Singh, B., 2024. Mineralisation and priming effects of a biodegradable plastic mulch film in soils: Influence of soil type, temperature and plastic particle size. Soil Biology and Biochemistry 189, 109257.
100. Xiao, Q., Zhang, W., Wu, L., Huang, Y., Cai, Z., Li, D., Xu, X., Hartley, I.P., 2024. Long-term liming mitigates the positive responses of soil carbon mineralization to warming and labile carbon input. Journal of Environmental Management 354, 120498.
101. Jia, K., Nie, S., Tian, M., Sun, W., Gao, Y., Zhang, Y., Xie, X., Xu, Z., Zhao, C., Li, C., 2024. Biodegradable microplastics can cause more serious loss of soil organic carbon by priming effect than conventional microplastics in farmland shelterbelts. Functional Ecology 38, 2447–2458.
102. Zhang, G., Liu, D., Lin, J., Kumar, A., Jia, K., Tian, X., Yu, Z., Zhu, B., 2023. Priming effects induced by degradable microplastics in agricultural soils. Soil Biology and Biochemistry 180, 109006.
103. Chen, L., Han, L., Wang, F., Chen, Q., Huang, H., Wang, J., Ma, C., Sun, K., Rillig, M.C., Kuzyakov, Y., Yang, Z., 2025. Polylactic acid microplastics induced negative priming and improved carbon sequestration via microbial processes in different paddy soils. Soil Biology and Biochemistry 201, 109653.
104. Bernal, B., McKinley, D.C., Hungate, B.A., White, P.M., Mozdzer, T.J., Megonigal, J.P., 2016. Limits to soil carbon stability; Deep, ancient soil carbon decomposition stimulated by new labile organic inputs. Soil Biology and Biochemistry 98, 85-94.
105. Dimassi, B., Mary, B., Fontaine, S., Perveen, N., Revaillot, S., Cohan, J.-P., 2014. Effect of nutrients availability and long-term tillage on priming effect and soil C mineralization. Soil Biology and Biochemistry 78, 332-339.
106. Wang, H., Xu, W., Hu, G., Dai, W., Jiang, P., Bai, E., 2015. The priming effect of soluble carbon inputs in organic and mineral soils from a temperate forest. Oecologia 178, 1239-1250.
107. Meyer, N., Welp, G., Rodionov, A., Borchard, N., Martius, C., Amelung, W., 2018. Nitrogen and phosphorus supply controls soil organic carbon mineralization in tropical topsoil and subsoil. Soil Biology and Biochemistry 119, 152-161.
108. Wang, Q., Wang, Y., Wang, S., He, T., Liu, L., 2014. Fresh carbon and nitrogen inputs alter organic carbon mineralization and microbial community in forest deep soil layers. Soil Biology and Biochemistry 72, 145-151.
109. Jílková, V., Jandová, K., Kukla, J., 2021. Responses of microbial activity to carbon, nitrogen, and phosphorus additions in forest mineral soils differing in organic carbon content. Biology and Fertility of Soils 57, 513-521.
110. Kan, Z.R., Virk, A.L., Wu, G., Qi, J.Y., Ma, S.T., Wang, X., Zhao, X., Lal, R., Zhang, H.L., 2020. Priming effect intensity of soil organic carbon mineralization under no-till and residue retention. Applied Soil Ecology 147, 103445.
111. Chen, Y., Li, W., You, Y., Ye, C., Shu, X., Zhang, Q., Zhang, K., 2022. Soil properties and substrate quality determine the priming of soil organic carbon during vegetation succession. Plant and Soil 471, 559–575.
112. Li, S., Jiang, Y., Lyu, M., Deng, C., Deng, W., Wang, M., Liu, J., Lu, Y., Xie, J., 2024. High-quality litter exerts a greater effect on soil carbon gain in unrestored than restored pine plantations. Science of the Total Environment 952, 175975.
113. Liu, Q., Zhu, Z., Abdalla, K., Ge, T., Wu, X., Kuzyakov, Y., Pausch, J., 2024. Microbial response to long-term fertilization of paddy soils: Apparent and real priming effects. Geoderma 445, 116884.
114. Chen, S., Guo, J., Guo, R., Huang, B., Huang, J., Wang, M., Shen, Q., Ling, N., Guo, S., 2024. Deciphering the active bacteria involving glucose-triggered priming effect in soils with gradient N inputs. Soil Biology and Biochemistry 199, 109612.
115. Qin, W., Feng, J., Zhang, Q., Yuan, X., Zhou, H., Zhu, B., 2024. Nitrogen and phosphorus addition mediate soil priming effects via affecting microbial stoichiometric balance in an alpine meadow. Science of the Total Environment 908, 168350.
116. Zhou, Jiashu, Zhang, S., Lv, J., Tang, C., Zhang, H., Fang, Y., Tavakkoli, E., Ge, T., Luo, Y., Cai, Y., Yu, B., White, J.C., Li, Y., 2024. Maize straw increases while its biochar decreases native organic carbon mineralization in a subtropical forest soil. Science of the Total Environment 939, 173606.
117. Zhou, Jie, Feng, W., Brown, R.W., Yang, H., Shao, G., Shi, L., Gui, H., Xu, J., Li, F.-M., Jones, D.L., Zamanian, K., 2024. Microplastic contamination accelerates soil carbon loss through positive priming. Science of the Total Environment 954, 176273.
118. Lu, W., Zhang, H., 2015. Response of biochar induced carbon mineralization priming effects to additional nitrogen in a sandy loam soil. Applied Soil Ecology 96, 165-171.
119. Bai, X., Zhai, G., Yan, Z., An, S., Liu, J., Huo, L., Dippold, M.A., Kuzyakov, Y., 2024. Effects of microbial groups on soil organic carbon accrual and mineralization during high- and low-quality litter decomposition. Catena 241, 108051.
120. Zhou, Y., Zhao, Z., Li, D., Wu, L., Chen, Z., An, N., Yang, J., Wang, Y., 2024. Effects of combined biochar and maize straw applications on soil greenhouse gas emissions. Applied Soil Ecology 202, 105540.
121. Chang, Q., Liu, Z., Zhang, T., Liu, S., Liu, B., Fan, X., Meng, D., Zhang, K., Bai, E., 2024. Soil priming effect in the organic and mineral layers regulated by nitrogen mining mechanism in a temperate forest. Land Degradation and Development 35, 4961–4971.
122. Cui, J., Wei, L., Zhu, Z., Deng, Y., Chen, S., Kuzyakov, Y., Ge, T., 2024. Influence of soil N availability on priming effects depending on temperature. Soil and Tillage Research 242, 106163.
123. Guliyev, V., Tanunchai, B., Udovenko, M., Menyailo, O., Glaser, B., Purahong, W., Buscot, F., Blagodatskaya, E., 2023. Degradation of bio-based and biodegradable plastic and its contribution to soil organic carbon stock. Polymers 15, 660.
124. Siegwart, L., Piton, G., Jourdan, C., Piel, C., Sauze, J., Sugihara, S., Bertrand, I., 2023. Carbon and nutrient colimitations control the microbial response to fresh organic carbon inputs in soil at different depths. Geoderma 440, 116729.
125. Su, J., Zhang, H., Han, X., Lv, R., Liu, L., Jiang, Y., Li, H., Kuzyakov, Y., Wei, C., 2023. 5300‐year‐old soil carbon is less primed than young soil organic matter. Global Change Biology 29, 260-275.
126. Wang, J., Wang, Y., Xue, R., Wang, D., Nan, W., 2023. Effects of defoliation and nitrogen on carbon dioxide (CO_2_ ) emissions and microbial communities in soils of cherry tree orchards. PeerJ 11, e15276.
127. Zhang, Y., Lu, M., Wang, Z., Zhang, K., Zhang, B., Naimaiti, R., Wei, S., Ding, X., 2024. Nutrient supply enhances positive priming of soil organic C under straw amendment and accelerates the incorporation of straw-derived C into organic C pool in paddy soils. European Journal of Soil Biology 123, 103695.
128. Ma, T., Zhan, Y., Chen, W., Hou, Z., Chai, S., Zhang, J., Zhang, X., Wang, R., Liu, R., Wei, Y., 2024. Microbial traits drive soil priming effect in response to nitrogen addition along an alpine forest elevation gradient. Science of the Total Environment 907, 167970.
129. Yu, Z., Chen, L., Pan, S., Li, Y., Kuzyakov, Y., Xu, J., Brookes, P.C., Luo, Y., 2018. Feedstock determines biochar‐induced soil priming effects by stimulating the activity of specific microorganisms. European Journal of Soil Science 69, 521-534.
130. Liu, Y., Chen, Y., Wang, Y., Lu, H., He, L., Yang, S., 2018. Negative priming effect of three kinds of biochar on the mineralization of native soil organic carbon. Land Degradation and Development 29, 3985-3994.
131. Yang, S., Zhao, X., Sun, Z., Wang, L., Tian, P., Wang, Q., 2024. Carbon and nitrogen addition-derived enzyme activities in topsoil but nitrogen availability in subsoil controls the response of soil organic carbon decomposition to warming. Science of the Total Environment 949, 175261.
132. Boos, E.F., Bruun, S., Magid, J., 2023. Priming is frequently overestimated in studies using ^14^C-labelled substrates due to underestimation of ^14^CO_2_ activity. Soil Biology and Biochemistry 181, 109020.
133. Heitkötter, J., Heinze, S., Marschner, B., 2017a. Relevance of substrate quality and nutrients for microbial C-turnover in top- and subsoil of a Dystric Cambisol. Geoderma 302, 89-99.
134. Heitkötter, J., Niebuhr, J., Heinze, S., Marschner, B., 2017b. Patterns of nitrogen and citric acid induced changes in C-turnover and enzyme activities are different in topsoil and subsoils of a sandy Cambisol. Geoderma 292, 111-117.
135. Herre, M., Heinze, S., Heitkötter, J., Marschner, B., 2022. Different factors control organic matter degradation in bulk and rhizosphere soil from the top- and subsoils of three forest stands. Soil Biology and Biochemistry 172, 108775.
136. Hoang, D.T.T., Bauke, S.L., Kuzyakov, Y., Pausch, J., 2017. Rolling in the deep: Priming effects in earthworm biopores in topsoil and subsoil. Soil Biology and Biochemistry 114, 59-71.
137. Jia, J., Feng, X., He, J.-S., He, H., Lin, L., Liu, Z., 2017. Comparing microbial carbon sequestration and priming in the subsoil versus topsoil of a Qinghai-Tibetan alpine grassland. Soil Biology and Biochemistry 104, 141-151.
138. Karhu, K., Hilasvuori, E., Fritze, H., Biasi, C., Nykänen, H., Liski, J., Vanhala, P., Heinonsalo, J., Pumpanen, J., 2016. Priming effect increases with depth in a boreal forest soil. Soil Biology and Biochemistry 99, 104-107.
139. Liao, C., Li, D., Huang, L., Yue, P., Liu, F., Tian, Q., 2020. Higher carbon sequestration potential and stability for deep soil compared to surface soil regardless of nitrogen addition in a subtropical forest. PeerJ 8, e9128.
140. Lü, M., Xie, J., Wang, C., Guo, J., Wang, M., Liu, X., Chen, Y., Chen, G., Yang, Y., 2015. Forest conversion stimulated deep soil C losses and decreased C recalcitrance through priming effect in subtropical China. Biology and Fertility of Soils 51, 857-867.
141. Perveen, N., Barot, S., Maire, V., Cotrufo, M.F., Shahzad, T., Blagodatskaya, E., Stewart, C.E., Ding, W., Siddiq, M.R., Dimassi, B., Mary, B., Fontaine, S., 2019. Universality of priming effect: An analysis using thirty five soils with contrasted properties sampled from five continents. Soil Biology and Biochemistry 134, 162-171.
142. Qiu, G., Zhu, M., Xu, J., Brookes, P.C., 2020. Evaluating C sources and microbial biomass dynamics involved in the triggering response with soil depth. Soil Biology and Biochemistry 149, 107958.
143. Rukshana, F., Butterly, C.R., Xu, J.M., Baldock, J.A., Tang, C., 2013. Soil organic carbon contributes to alkalinity priming induced by added organic substrates. Soil Biology and Biochemistry 65, 217-226.
144. Shahzad, T., Anwar, F., Hussain, S., Mahmood, F., Arif, M.S., Sahar, A., Nawaz, M.F., Perveen, N., Sanaullah, M., Rehman, K., Rashid, M.I., 2019. Carbon dynamics in surface and deep soil in response to increasing litter addition rates in an agro-ecosystem. Geoderma 333, 1-9.
145. Struecker, J., Dyckmans, J., Joergensen, R.G., 2017. Plant residue and native organic matter decomposition under subsoil-specific gas conditions – Comparing topsoils with C-poor and C-rich subsoils. Geoderma 292, 1-8.
146. Tian, Q., Yang, X., Wang, X., Liao, C., Li, Q., Wang, M., Wu, Y., Liu, F., 2016. Microbial community mediated response of organic carbon mineralization to labile carbon and nitrogen addition in topsoil and subsoil. Biogeochemistry 128, 125-139.
147. Walz, J., Knoblauch, C., Böhme, L., Pfeiffer, E.-M., 2017. Regulation of soil organic matter decomposition in permafrost-affected Siberian tundra soils - Impact of oxygen availability, freezing and thawing, temperature, and labile organic matter. Soil Biology and Biochemistry 110, 34-43.
148. Wild, B., Gentsch, N., Čapek, P., Diáková, K., Alves, R.J.E., Bárta, J., Gittel, A., Hugelius, G., Knoltsch, A., Kuhry, P., Lashchinskiy, N., Mikutta, R., Palmtag, J., Schleper, C., Schnecker, J., Shibistova, O., Takriti, M., Torsvik, V.L., Urich, T., Watzka, M., Šantrůčková, H., Guggenberger, G., Richter, A., 2016. Plant-derived compounds stimulate the decomposition of organic matter in arctic permafrost soils. Scientific Reports 6, 25607.
149. Wild, B., Schnecker, J., Alves, R.J.E., Barsukov, P., Bárta, J., Čapek, P., Gentsch, N., Gittel, A., Guggenberger, G., Lashchinskiy, N., Mikutta, R., Rusalimova, O., Šantrůčková, H., Shibistova, O., Urich, T., Watzka, M., Zrazhevskaya, G., Richter, A., 2014. Input of easily available organic C and N stimulates microbial decomposition of soil organic matter in arctic permafrost soil. Soil Biology and Biochemistry 75, 143-151.
150. Zhang, M., Dong, L.G., Fei, S.X., Zhang, J.W., Jiang, X.M., Wang, Y., Yu, X., 2021. Responses of soil organic carbon mineralization and microbial communities to leaf litter addition under different soil layers. Forests 12, 170.
151. Auwal, M., Singh, B.P., Chen, Z., Kumar, A., Pan, S., Luo, Y., Xu, J., 2021. Nutrients addition regulates temperature sensitivity of maize straw mineralization. Journal of Soils and Sediments 21, 2778-2790.
152. Aye, N.S., Butterly, C.R., Sale, P.W.G., Tang, C., 2018. Interactive effects of initial pH and nitrogen status on soil organic carbon priming by glucose and lignocellulose. Soil Biology and Biochemistry 123, 33-44.
153. Blagodatskaya, E.V., Blagodatsky, S.A., Anderson, T.H., Kuzyakov, Y., 2007. Priming effects in Chernozem induced by glucose and N in relation to microbial growth strategies. Applied Soil Ecology 37, 95-105.
154. Chen, R., Senbayram, M., Blagodatsky, S., Myachina, O., Dittert, K., Lin, X., Blagodatskaya, E., Kuzyakov, Y., 2014. Soil C and N availability determine the priming effect: microbial N mining and stoichiometric decomposition theories. Global Change Biology 20, 2356-2367.
155. Chowdhury, S., Farrell, M., Bolan, N., 2014. Priming of soil organic carbon by malic acid addition is differentially affected by nutrient availability. Soil Biology and Biochemistry 77, 158-169.
156. Chen, L., Liu, L., Mao, C., Qin, S., Wang, J., Liu, F., Blagodatsky, S., Yang, G., Zhang, Q., Zhang, D., Yu, J., Yang, Y., 2018. Nitrogen availability regulates topsoil carbon dynamics after permafrost thaw by altering microbial metabolic efficiency. Nature Communications 9, 3951.
157. Di Lonardo, D.P., Manrubia, M., De Boer, W., Zweers, H., Veen, G.F., Van Der Wal, A., 2018. Relationship between home-field advantage of litter decomposition and priming of soil organic matter. Soil Biology and Biochemistry 126, 49-56.
158. Fang, Y., Nazaries, L., Singh, B.K., Singh, B.P., 2018. Microbial mechanisms of carbon priming effects revealed during the interaction of crop residue and nutrient inputs in contrasting soils. Global Change Biology 24, 2775-2790.
159. Fontaine, S., Henault, C., Aamor, A., Bdioui, N., Bloor, J.M.G., Maire, V., Mary, B., Revaillot, S., Maron, P.A., 2011. Fungi mediate long term sequestration of carbon and nitrogen in soil through their priming effect. Soil Biology and Biochemistry 43, 86-96.
160. Fu, X., Song, Q., Li, S., Shen, Y., Yue, S., 2022. Dynamic changes in bacterial community structure are associated with distinct priming effect patterns. Soil Biology and Biochemistry 169, 108671.
161. Fan, Y., Zhong, X., Lin, T.C., Lyu, M., Wang, M., Hu, W., Yang, Z., Chen, G., Guo, J., Yang, Y., 2020. Effects of nitrogen addition on DOM-induced soil priming effects in a subtropical plantation forest and a natural forest. Biology and Fertility of Soils 56, 205-216.
162. Guttières, R., Nunan, N., Raynaud, X., Lacroix, G., Barot, S., Barré, P., Girardin, C., Guenet, B., Lata, J.C., Abbadie, L., 2021. Temperature and soil management effects on carbon fluxes and priming effect intensity. Soil Biology and Biochemistry 153, 108103.
163. Gaudel, G., Xing, L., Shrestha, S., Poudel, M., Sherpa, P., Raseduzzaman, M., Zhang, X., 2024. Microbial mechanisms regulate soil organic carbon mineralization under carbon with varying levels of nitrogen addition in the above-treeline ecosystem. Science of the Total Environment 917, 170497.
164. Guenet, B., Neill, C., Bardoux, G., Abbadie, L., 2010. Is there a linear relationship between priming effect intensity and the amount of organic matter input? Applied Soil Ecology 46, 436-442.
165. Hartley, I.P., Hopkins, D.W., Sommerkorn, M., Wookey, P.A., 2010. The response of organic matter mineralisation to nutrient and substrate additions in sub-arctic soils. Soil Biology and Biochemistry 42, 92-100.
166. Hicks, L.C., Meir, P., Nottingham, A.T., Reay, D.S., Stott, A.W., Salinas, N., Whitaker, J., 2019. Carbon and nitrogen inputs differentially affect priming of soil organic matter in tropical lowland and montane soils. Soil Biology and Biochemistry 129, 212-222.
167. Jiang, X., Haddix, M.L., Cotrufo, M.F., 2016. Interactions between biochar and soil organic carbon decomposition: Effects of nitrogen and low molecular weight carbon compound addition. Soil Biology and Biochemistry 100, 92-101.
168. Kaštovská, E., Šantrůčková, H., Picek, T., Vašková, M., Edwards, K.R., 2010. Direct effect of fertilization on microbial carbon transformation in grassland soils in dependence on the substrate quality. Journal of Plant Nutrition and Soil Science 173, 706-714.
169. Li, L.J., Zhu-Barker, X., Ye, R., Doane, T.A., Horwath, W.R., 2018. Soil microbial biomass size and soil carbon influence the priming effect from carbon inputs depending on nitrogen availability. Soil Biology and Biochemistry 119, 41-49.
170. Li, Q., Tian, Y., Zhang, X., Xu, X., Wang, H., Kuzyakov, Y., 2017. Labile carbon and nitrogen additions affect soil organic matter decomposition more strongly than temperature. Applied Soil Ecology 114, 152-160.
171. Lu, W., Ding, W., Zhang, J., Li, Y., Luo, J., Bolan, N., Xie, Z., 2014. Biochar suppressed the decomposition of organic carbon in a cultivated sandy loam soil: A negative priming effect. Soil Biology and Biochemistry 76, 12-21.
172. Maestrini, B., Herrmann, A.M., Nannipieri, P., Schmidt, M.W.I., Abiven, S., 2014. Ryegrass-derived pyrogenic organic matter changes organic carbon and nitrogen mineralization in a temperate forest soil. Soil Biology and Biochemistry 69, 291-301.
173. Meng, F., Dungait, J.A.J., Xu, X., Bol, R., Zhang, X., Wu, W., 2017. Coupled incorporation of maize (*Zea mays* L.) straw with nitrogen fertilizer increased soil organic carbon in Fluvic Cambisol. Geoderma 304, 19-27.
174. Nele Meyer, Gerhard Welp, Ludger Bornemann, Wulf Amelung, 2017. Microbial nitrogen mining affects spatio-temporal patterns of substrate-induced respiration during seven years of bare fallow. Soil Biology and Biochemistry 104, 175-184.
175. Mganga, K.Z., Kuzyakov, Y., 2018. Land use and fertilisation affect priming in tropical andosols. European Journal of Soil Biology 87, 9-16.
176. Qiu, Q., Wu, L., Ouyang, Z., Li, B., Xu, Y., 2016a. Different effects of plant-derived dissolved organic matter (DOM) and urea on the priming of soil organic carbon. Environmental Science: Processes and Impacts 18, 330-341.
177. Qiu, Q., Wu, L., Ouyang, Z., Li, B., Xu, Y., Wu, S., Gregorich, E.G., 2016b. Priming effect of maize residue and urea N on soil organic matter changes with time. Applied Soil Ecology 100, 65-74.
178. Soong, J.L., Marañon-Jimenez, S., Cotrufo, M.F., Boeckx, P., Bodé, S., Guenet, B., Peñuelas, J., Richter, A., Stahl, C., Verbruggen, E., Janssens, I.A., 2018. Soil microbial CNP and respiration responses to organic matter and nutrient additions: Evidence from a tropical soil incubation. Soil Biology and Biochemistry 122, 141-149.
179. Su, Z., Shangguan, Z., 2023. Nitrogen addition decreases the soil cumulative priming effect and favours soil net carbon gains in Robinia pseudoacacia plantation soil. Geoderma 433, 116444.
180. Wang, D., Zhu, Z., Shahbaz, M., Chen, L., Liu, S., Inubushi, K., Wu, J., Ge, T., 2019. Split N and P addition decreases straw mineralization and the priming effect of a paddy soil: a 100-day incubation experiment. Biology and Fertility of Soils 55, 701-712.
181. Wang, H., Hu, G., Xu, W., Boutton, T.W., Zhuge, Y., Bai, E., 2018. Effects of nitrogen addition on soil organic carbon mineralization after maize stalk addition. European Journal of Soil Biology 89, 33-38.
182. Wang, Q., Wang, S., He, T., Liu, L., Wu, J., 2014. Response of organic carbon mineralization and microbial community to leaf litter and nutrient additions in subtropical forest soils. Soil Biology and Biochemistry 71, 13-20.
183. Wang, X., Lu, J., Zhang, X., Wang, P., 2021. Contrasting microbial mechanisms of soil priming effects induced by crop residues depend on nitrogen availability and temperature. Applied Soil Ecology 168, 104186.
184. Zhang, S., Fang, Y., Kawasaki, A., Tavakkoli, E., Cai, Y., Wang, H., Ge, T., Zhou, J., Yu, B., Li, Y., 2023. Biochar significantly reduced nutrient-induced positive priming in a subtropical forest soil. Biology and Fertility of Soils 59, 589-607.
185. Zheng, Y., Jin, J., Wang, X., Clark, G.J., Tang, C., 2022. Increasing nitrogen availability does not decrease the priming effect on soil organic matter under pulse glucose and single nitrogen addition in woodland topsoil. Soil Biology and Biochemistry 172, 108767.
186. Zhu, Z., Ge, T., Luo, Y., Liu, S., Xu, X., Tong, C., Shibistova, O., Guggenberger, G., Wu, J., 2018. Microbial stoichiometric flexibility regulates rice straw mineralization and its priming effect in paddy soil. Soil Biology and Biochemistry 121, 67-76.
187. He, P., Li, L.-J., Dai, S.S., Guo, X.L., Nie, M., Yang, X., Kuzyakov, Y., 2024. Straw addition and low soil moisture decreased temperature sensitivity and activation energy of soil organic matter. Geoderma 442, 116802.
188. Khan, A., Wichern, F., Uporova, M., Kuzyakov, Y., 2024. Mineralization and temperature sensitivity of soil organic matter pools of contrasting lability. European Journal of Soil Science 75, e13451.
189. Ahmad, W., Singh, B., Dijkstra, F.A., Dalal, R.C., Geelan-Small, P., 2014. Temperature sensitivity and carbon release in an acidic soil amended with lime and mulch. Geoderma 214, 168–176.
190. Börger, M., Bublitz, T., Dyckmans, J., Wachendorf, C., Joergensen, R.G., 2022. Microbial carbon use efficiency of litter with distinct C/N ratios in soil at different temperatures, including microbial necromass as growth component. Biology and Fertility of Soils 58, 761-770.
191. Chen, X., Lin, J., Wang, P., Zhang, S., Liu, D., Zhu, B., 2022. Resistant soil carbon is more vulnerable to priming effect than active soil carbon. Soil Biology and Biochemistry 168, 108619.
192. Creamer, C.A., De Menezes, A.B., Krull, E.S., Sanderman, J., Newton-Walters, R., Farrell, M., 2015. Microbial community structure mediates response of soil C decomposition to litter addition and warming. Soil Biology and Biochemistry 80, 175-188.
193. Cui, J., Ge, T., Nie, M., Kuzyakov, Y., Alharbi, S., Fang, C., Deng, Z., 2022. Contrasting effects of maize litter and litter-derived biochar on the temperature sensitivity of paddy soil organic matter decomposition. Frontiers in Microbiology 13, 1008744.
194. Fang, Y., Singh, B., Singh, B.P., 2015. Effect of temperature on biochar priming effects and its stability in soils. Soil Biology and Biochemistry 80, 136-145.
195. Fang, Y., Singh, B.P., Matta, P., Cowie, A.L., Van Zwieten, L., 2017. Temperature sensitivity and priming of organic matter with different stabilities in a Vertisol with aged biochar. Soil Biology and Biochemistry 115, 346-356.
196. Feng, C., Sun, H., Zhang, Y., 2021. The magnitude and direction of priming were driven by soil moisture and temperature in a temperate forest soil of China. Pedobiologia 89, 150769.
197. Frøseth, R.B., Bleken, M.A., 2015. Effect of low temperature and soil type on the decomposition rate of soil organic carbon and clover leaves, and related priming effect. Soil Biology and Biochemistry 80, 156-166.
198. Gogoi, L., Narzari, R., Gogoi, N., Borkotoki, B., Kataki, R., 2020. Effect of Biochar on Soil Respiration from a Semi-evergreen, Moist Deciduous Forest Soil. Int. International Journal of Geosynthetics and Ground Engineering 6, 26.
199. Hopkins, F.M., Filley, T.R., Gleixner, G., Lange, M., Top, S.M., Trumbore, S.E., 2014. Increased belowground carbon inputs and warming promote loss of soil organic carbon through complementary microbial responses. Soil Biology and Biochemistry 76, 57-69.
200. Jiang, L., Ma, X., Song, Y., Gao, S., Ren, J., Zhang, H., Wang, X., 2022. Warming-Induced labile carbon change soil organic carbon mineralization and microbial abundance in a Northern peatland. Microorganisms 10, 1329.
201. Lenka, S., Trivedi, P., Singh, B., Singh, B.P., Pendall, E., Bass, A., Lenka, N.K., 2019. Effect of crop residue addition on soil organic carbon priming as influenced by temperature and soil properties. Geoderma 347, 70-79.
202. Liu, Q., Xu, X., Wang, H., Blagodatskaya, E., Kuzyakov, Y., 2019. Dominant extracellular enzymes in priming of SOM decomposition depend on temperature. Geoderma 343, 187-195.
203. Li, J., Jian, S., De Koff, J.P., Lane, C.S., Wang, G., Mayes, M.A., Hui, D., 2018. Differential effects of warming and nitrogen fertilization on soil respiration and microbial dynamics in switchgrass croplands. Global Change Biology Bioenergy 10, 565-576.
204. Li, H., Liu, G., Luo, H., Zhang, R., 2022. Labile carbon input and temperature effects on soil organic matter turnover in subtropical forests. Ecological Indicators 145, 109726.
205. Wang, Q., He, T., Liu, J., 2016. Litter input decreased the response of soil organic matter decomposition to warming in two subtropical forest soils. Scientific Reports 6, 33814.
206. You, M., He, P., Dai, S.S., Burger, M., Li, L.-J., 2021. Priming effect of stable C pool in soil and its temperature sensitivity. Geoderma 401, 115216.
207. Zhang, D., Gong, C., Zhang, W., Zhang, H., Zhang, J., Song, C., 2021. Labile carbon addition alters soil organic carbon mineralization but not its temperature sensitivity in a freshwater marsh of Northeast China. Applied Soil Ecology 160, 103844.
208. Wang, H., Boutton, T.W., Xu, W., Hu, G., Jiang, P., Bai, E., 2015. Quality of fresh organic matter affects priming of soil organic matter and substrate utilization patterns of microbes. Scientific Reports 5, 10102.
209. Kuzyakov, Y., Bol, R., 2006. Sources and mechanisms of priming effect induced in two grassland soils amended with slurry and sugar. Soil Biology and Biochemistry 38, 747-758.
210. Guenet, B., Juarez, S., Bardoux, G., Abbadie, L., Chenu, C., 2012. Evidence that stable C is as vulnerable to priming effect as is more labile C in soil. Soil Biology and Biochemistry 52, 43-48.
211. Ye, R., Doane, T.A., Morris, J., Horwath, W.R., 2015. The effect of rice straw on the priming of soil organic matter and methane production in peat soils. Soil Biology and Biochemistry 81, 98-107.
212. Luo, Y., Zang, H., Yu, Z., Chen, Z., Gunina, A., Kuzyakov, Y., Xu, J., Zhang, K., Brookes, P.C., 2017. Priming effects in biochar enriched soils using a three-source-partitioning approach: ^14^C labelling and ^13^C natural abundance. Soil Biology and Biochemistry 106, 28-35.
213. Shahbaz, M., Kuzyakov, Y., Heitkamp, F., 2017. Decrease of soil organic matter stabilization with increasing inputs: Mechanisms and controls. Geoderma 304, 76-82.
214. Finley, B.K., Dijkstra, P., Rasmussen, C., Schwartz, E., Mau, R.L., Liu, X.J.A., Van Gestel, N., Hungate, B.A., 2018. Soil mineral assemblage and substrate quality effects on microbial priming. Geoderma 322, 38-47.
215. Falchini, L., Naumova, N., Kuikman, P.J., Bloem, J., Nannipieri, P., 2003. CO_2_ evolution and denaturing gradient gel electrophoresis profiles of bacterial communities in soil following addition of low molecular weight substrates to simulate root exudation. Soil Biology and Biochemistry 35, 775-782.
216. Hamer, U., Marschner, B., 2005. Priming effects in different soil types induced by fructose, alanine, oxalic acid and catechol additions. Soil Biology and Biochemistry 37, 445-454.
217. Blagodatsky, S., Blagodatskaya, E., Yuyukina, T., Kuzyakov, Y., 2010. Model of apparent and real priming effects: Linking microbial activity with soil organic matter decomposition. Soil Biology and Biochemistry 42, 1275-1283.
218. Blagodatskaya, E., Yuyukina, T., Blagodatsky, S., Kuzyakov, Y., 2011. Three-source-partitioning of microbial biomass and of CO_2_ efflux from soil to evaluate mechanisms of priming effects. Soil Biology and Biochemistry 43, 778-786.
219. Zimmerman, A.R., Gao, B., Ahn, M.Y., 2011. Positive and negative carbon mineralization priming effects among a variety of biochar-amended soils. Soil Biology and Biochemistry 43, 1169-1179.
220. Zhang, W., Wang, S., 2012. Effects of NH_4_^+^ and NO_3_^−^ on litter and soil organic carbon decomposition in a Chinese fir plantation forest in South China. Soil Biology and Biochemistry 47, 116-122.
221. Santos, F., Torn, M.S., Bird, J.A., 2012. Biological degradation of pyrogenic organic matter in temperate forest soils. Soil Biology and Biochemistry 51, 115-124.
222. Bastida, F., Torres, I.F., Hernández, T., Bombach, P., Richnow, H.H., García, C., 2013. Can the labile carbon contribute to carbon immobilization in semiarid soils? Priming effects and microbial community dynamics. Soil Biology and Biochemistry 57, 892-902.
223. Blagodatskaya, E., Khomyakov, N., Myachina, O., Bogomolova, I., Blagodatsky, S., Kuzyakov, Y., 2014. Microbial interactions affect sources of priming induced by cellulose. Soil Biology and Biochemistry 74, 39-49.
224. Zhang, Y., Yao, S., Mao, J., Olk, D.C., Cao, X., Zhang, B., 2015. Chemical composition of organic matter in a deep soil changed with a positive priming effect due to glucose addition as investigated by ^13^C NMR spectroscopy. Soil Biology and Biochemistry 85, 137-144.
225. Vestergård, M., Reinsch, S., Bengtson, P., Ambus, P., Christensen, S., 2016. Enhanced priming of old, not new soil carbon at elevated atmospheric CO_2_. Soil Biology and Biochemistry 100, 140-148.
226. Singh, M., Sarkar, B., Biswas, B., Bolan, N.S., Churchman, G.J., 2017. Relationship between soil clay mineralogy and carbon protection capacity as influenced by temperature and moisture. Soil Biology and Biochemistry 109, 95-106.
227. Cui, J., Ge, T., Kuzyakov, Y., Nie, M., Fang, C., Tang, B., Zhou, C., 2017. Interactions between biochar and litter priming: A three-source ^14^C and δ^13^C partitioning study. Soil Biology and Biochemistry 104, 49–58.
228. Mason-Jones, K., Kuzyakov, Y., 2017. “Non-metabolizable” glucose analogue shines new light on priming mechanisms: Triggering of microbial metabolism. Soil Biology and Biochemistry 107, 68-76.
229. Lian, T., Jin, J., Wang, G., Tang, C., Yu, Z., Li, Y., Liu, J., Zhang, S., Liu, X., 2017. The fate of soybean residue-carbon links to changes of bacterial community composition in Mollisols differing in soil organic carbon. Soil Biology and Biochemistry 109, 50-58.
230. Shahbaz, M., Kumar, A., Kuzyakov, Y., Börjesson, G., Blagodatskaya, E., 2018. Interactive priming effect of labile carbon and crop residues on SOM depends on residue decomposition stage: Three-source partitioning to evaluate mechanisms. Soil Biology and Biochemistry 126, 179-190.
231. Chotte, J.L., Ladd, J.N., Amato, M., 1998. Sites of microbial assimilation, and turnover of soluble and particulate ^14^C-labelled substrates decomposing in a clay soil. Soil Biology and Biochemistry 30, 205-218.
232. Sauvadet, M., Lashermes, G., Alavoine, G., Recous, S., Chauvat, M., Maron, P.A., Bertrand, I., 2018. High carbon use efficiency and low priming effect promote soil C stabilization under reduced tillage. Soil Biology and Biochemistry 123, 64-73.
233. Magid, J., Kjærgaard, C., Gorissen, A., Kuikman, P.J., 1999. Drying and rewetting of a loamy sand soil did not increase the turnover of native organic matter, but retarded the decomposition of added ^14^C-labelled plant material. Soil Biology and Biochemistry 31, 595-602.
234. Nottingham, A.T., Griffiths, H., Chamberlain, P.M., Stott, A.W., Tanner, E.V.J., 2009. Soil priming by sugar and leaf-litter substrates: A link to microbial groups. Applied Soil Ecology 42, 183-190.
235. Zhang, W., Wang, X., Wang, S., 2014. Fate of Chinese-fir litter during decomposition as a result of inorganic N additions. Applied Soil Ecology 74, 30-36.
236. Chen, L., Zhang, J.B., Zhao, B.Z., Xin, X.L., Zhou, G.X., Tan, J.F., Zhao, J.H., 2014. Carbon Mineralization and Microbial Attributes in Straw-Amended Soils as Affected by Moisture Levels. Pedosphere 24, 167-177.
237. Eck, T., Potthoff, M., Dyckmans, J., Wichern, F., Joergensen, R.G., 2015. Priming effects of Aporrectodea caliginosa on young rhizodeposits and old soil organic matter following wheat straw addition. European Journal of Soil Biology 70, 38-45.
238. Zhang, H., Ding, W., Luo, J., Bolan, N., Yu, H., Zhu, J., 2016. Temporal responses of microorganisms and native organic carbon mineralization to ^13^C-glucose addition in a sandy loam soil with long-term fertilization. European Journal of Soil Biology 74, 16-22.
239. Liang, X., Yuan, J., Yang, E., Meng, J., 2017. Responses of soil organic carbon decomposition and microbial community to the addition of plant residues with different C:N ratio. European Journal of Soil Biology 82, 50-55.
240. Wu, J., Brookes, P.C., Jenkinson, D.S., 1993. Formation and destruction of microbial biomass during the decomposition of glucose and ryegrass in soil. Soil Biology and Biochemistry 25, 1435-1441.
241. Dilly, O., Zyakun, A., 2008. Priming effect and respiratory quotient in a forest soil amended with glucose. Geomicrobiology Journal 25, 425-431.
242. Luna-Guido, M., Vega-Estrada, J., Ponce-Mendoza, A. et al, 2003. Mineralization of ^14^C-labelled maize in alkaline saline soils. Plant and Soil 250, 29-38.
243. Zhang, X., Han, X., Yu, W., Wang, P., Cheng, W., 2017. Priming effects on labile and stable soil organic carbon decomposition: Pulse dynamics over two years. Plos One 12, e0184978.
244. Yin, Y., He, X., Gao, R., Ma, H., Yang, Y., 2014. Effects of rice straw and its biochar addition on soil labile carbon and soil organic carbon. Journal of Integrative Agriculture 13, 491-498.
245. Guenet, B., Leloup, J., Raynaud, X., Bardoux, G., Abbadie, L., 2010. Negative priming effect on mineralization in a soil free of vegetation for 80 years. European Journal of Soil Science 61, 384-391.
246. Herath, H.M.S.K., Camps‐Arbestain, M., Hedley, M.J., Kirschbaum, M.U.F., Wang, T., Van Hale, R., 2015. Experimental evidence for sequestering C with biochar by avoidance of CO_2_ emissions from original feedstock and protection of native soil organic matter. Global Change Biology Bioenergy 7, 512-526.
247. Yousaf, B., Liu, G., Wang, R., Abbas, Q., Imtiaz, M., Liu, R., 2017. Investigating the biochar effects on C‐mineralization and sequestration of carbon in soil compared with conventional amendments using the stable isotope (δ^13^C) approach. Global Change Biology Bioenergy 9, 1085-1099.
248. Qiao, N., Schaefer, D., Blagodatskaya, E., Zou, X., Xu, X., Kuzyakov, Y., 2014. Labile carbon retention compensates for CO_2_ released by priming in forest soils. Global Change Biology 20, 1943-1954.
249. Hu, L., Su, Y., He, X., Wu, J., Zheng, H., Li, Y., Wang, A., 2012. Response of soil organic carbon mineralization in typical Karst soils following the addition of ^14^C‐labeled rice straw and CaCO_3_. Journal of the Science of Food and Agriculture 92, 1112-1118.
250. Wu, J., Zhou, P., Li, L., Su, Y., Yuan, H., Syers, J.K., 2012. Restricted mineralization of fresh organic materials incorporated into a subtropical paddy soil. Journal of the Science of Food and Agriculture 92, 1031-1037.
251. Bol, R., Moering, J., Kuzyakov, Y., Amelung, W., 2003. Quantification of priming and CO_2_ respiration sources following slurry‐C incorporation into two grassland soils with different C content. Rapid Communications in Mass Spectrometry 17, 2585–2590.
252. Fontaine, S., Bardoux, G., Benest, D., Verdier, B., Mariotti, A., Abbadie, L., 2004. Mechanisms of the priming effect in a savannah soil amended with cellulose. Soil Science Society of America Journal 68, 125-131.
253. Aye, N.S., Butterly, C.R., Sale, P.W.G., Tang, C., 2017. Residue addition and liming history interactively enhance mineralization of native organic carbon in acid soils. Biology and Fertility of Soils 53, 61-75.
254. Shahbaz, M., Kuzyakov, Y., Sanaullah, M., Heitkamp, F., Zelenev, V., Kumar, A., Blagodatskaya, E., 2017. Microbial decomposition of soil organic matter is mediated by quality and quantity of crop residues: mechanisms and thresholds. Biology and Fertility of Soils 53, 287-301.
255. Lyu, M., Xie, J., Vadeboncoeur, M.A., Wang, M., Qiu, X., Ren, Y., Jiang, M., Yang, Y., Kuzyakov, Y., 2018. Simulated leaf litter addition causes opposite priming effects on natural forest and plantation soils. Biology and Fertility of Soils 54, 925-934.
256. Pascault, N., Ranjard, L., Kaisermann, A., Bachar, D., Christen, R., Terrat, S., Mathieu, O., Lévêque, J., Mougel, C., Henault, C., Lemanceau, P., Péan, M., Boiry, S., Fontaine, S., Maron, P.-A., 2013. Stimulation of different functional groups of bacteria by various plant residues as a driver of soil priming effect. Ecosystems 16, 810-822.
257. Wang, Q., Zeng, Z., Zhong, M., 2016. Soil moisture alters the response of soil organic carbon mineralization to litter addition. Ecosystems 19, 450-460.
258. Lienhard, P., Terrat, S., Mathieu, O., Levêque, J., Chemidlin Prévost-Bouré, N., Nowak, V., Régnier, T., Faivre, C., Sayphoummie, S., Panyasiri, K., Tivet, F., Ranjard, L., Maron, P.A., 2013. Soil microbial diversity and C turnover modified by tillage and cropping in Laos tropical grassland. Environmental Chemistry Letters 11, 391-398.
259. Miao, S., Ye, R., Qiao, Y., Zhu-Barker, X., Doane, T.A., Horwath, W.R., 2017. The solubility of carbon inputs affects the priming of soil organic matter. Plant and Soil 410, 129-138.
260. Schmatz, R., Recous, S., Aita, C., Tahir, M.M., Schu, A.L., Chaves, B., Giacomini, S.J., 2017. Crop residue quality and soil type influence the priming effect but not the fate of crop residue C. Plant and Soil 414, 229-245.
261. Naisse, C., Girardin, C., Davasse, B., Chabbi, A., Rumpel, C., 2015. Effect of biochar addition on C mineralisation and soil organic matter priming in two subsoil horizons. Journal of Soils and Sediments 15, 825-832.
262. Su, P., Lou, J., Brookes, P.C., Luo, Y., He, Y., Xu, J., 2017. Taxon-specific responses of soil microbial communities to different soil priming effects induced by addition of plant residues and their biochars. Journal of Soils and Sediments 17, 674-684.
263. Fu, X., Li, J., Kang, X., Chen, H., Li, S., 2025. The fungal community assembly was governed by deterministic selection during priming effects induced by residue addition. Plant and Soil 513, 1909-1925.
264. Wiesenbauer, J., Gorka, S., Jenab, K., Schuster, R., Kumar, N., Rottensteiner, C., König, A., Kraemer, S., Inselsbacher, E., Kaiser, C., 2025. Preferential use of organic acids over sugars by soil microbes in simulated root exudation. Soil Biology and Biochemistry 203, 109738.
265. Lin, J., Chen, B., Dong, H., Zhang, W., Kumar, A., Hui, D., Zhang, C., Shan, S., Zhu, B., 2025. Effects of soil moisture fluctuation and microplastics types on soil organic matter decomposition and carbon dynamics. Soil Biology and Biochemistry 205, 109781.
266. Zhou, S., Lin, J., Wang, P., Zhu, P., Zhu, B., 2023. Resistant soil organic carbon is more vulnerable to priming by root exudate fractions than relatively active soil organic carbon. Plant and Soil 488, 71-82.
267. You, M., Guo, D., Shi, H., He, P., Burger, M., Li, L.J., 2025. Microbial nutrient limitations and chemical composition of soil organic carbon regulate the organic carbon mineralization and temperature sensitivity in forest and grassland soils. Plant and Soil 514, 459-476.
268. Chen, M., Kuzyakov, Y., Zhou, J., Zamanian, K., Wang, S., Abdalla, K., Wang, J., Li, X., Li, H., Zhang, H., Mganga, K.Z., Li, Y., Blagodatskaya, E., 2025. High soil salinity reduces straw decomposition but primes soil organic carbon loss. Soil Biology and Biochemistry 207, 109835.
269. Chen, M., Kuzyakov, Y., Zhou, J., Zamanian, K., Wang, S., Abdalla, K., Wang, J., Li, X., Li, H., Zhang, H., Mganga, K.Z., Li, Y., Blagodatskaya, E., 2025. High soil salinity reduces straw decomposition but primes soil organic carbon loss. Soil Biology and Biochemistry 207, 109835.
270. Wang, H., Ye, W., He, W., Guo, Z., Hu, G., Lou, Y., Yang, Q., Yang, Z., Sun, Y., Pan, H., Zhuge, Y., 2025. Phosphorus addition increases soil organic matter priming in a coastal saline soil. Soil Biology and Biochemistry 208, 109862.
271. Dong, H., He, P., Liu, M., Kuzyakov, Y., Li, L.-J., 2025. Nitrogen availability governs priming effect induced by biodegradable microplastics through microbial life-strategies. European Journal of Soil Science 76(4), e70170.
272. Jiang, Z., Zhang, S., Tang, C., Xiao, M., Zhou, J., Luo, Y., Ge, T., Yu, B., White, J.C., Li, Y., 2025. Divergent effects of straw and biochar on soil carbon priming are depth-dependent in subtropical Moso bamboo forests. Biology and Fertility of Soils. https://doi.org/10.1007/s00374-025-01927-z.
273. Qiu, Q., Mao, Z., Gan, Z., Mgelwa, A.S., Leuzinger, S., Hu, Y., 2025. Low-quality litter promotes soil organic carbon accumulation by inhibiting priming effects and stimulating mineral-associated organic carbon formation. Journal of Soils and Sediments 25, 2584-2599.
274. Wang, R., Hou, J., Chen, L., He, L., Na, L., Wang, Y., Lu, H., Yang, S., Liu, Y., 2025. Priming effects of vermiculite modified rice straw biochar on soil organic carbon: a new perspective of soil bacteria. Biochar 7(1), 54.
275. Xiao, Q., Zhang, W., Wu, L., Huang, Y., Wang, J., Cai, Z., Li, D., Chen, X., Ge, T., Xu, M., Kuzyakov, Y., 2025. Sensitivity of soil organic matter priming to warming depends on diurnal temperature cycle. Journal of Agricultural and Food Chemistry 73(32), 19993-20003.
276. Wang, Q., Zhao, X., Liu, S., Wang, Q., Zhang, W., Fontaine, S., Zhu, B., Tian, P., 2024. Contrasting responses of the priming effect to nitrogen deposition in temperate and subtropical forests. Catena 238, 107839.
277. Koyama, T., Enggrob, K.L., Rasmussen, J., Martins, J.T., Peixoto, L., 2025. Substrate quantity and quality affect microbial carbon use efficiency and priming effects of root exudates investigated with microdialysis. Soil Biology and Biochemistry 209, 109869.
278. Bai, X., Zhang, Q., Feng, J., Wu, X., Zeng, Q., Zhang, X., Yuan, X., Ni, X., Chen, Y., 2025. Warming reduces soil priming effects in a subtropical forest regardless of soil depth and carbon input amount. Plant and Soil. <https://doi.org/10.1007/s11104-025-07849-x>.
279. Pan, J., Zheng, X., Liao, D., Jiang, Z., Li, X., Li, C., Li, Y., Zhao, J., Ma, X., Geng, Q., Zhang, C., Wu, Q., Dong, Z., 2025. Temperature fluctuation affects soil organic carbon accumulation through soil enzyme activity and nutrient limitation. Environmental Technology and Innovation 40, 104485.
280. Zhang, X., Wang, Z., Lü, T., Han, X., Yu, F., 2025. Contrasting priming effects in soils subjected to long-term urea versus inorganic nitrogen addition. Soil and Tillage Research 256, 106918.
281. Chen, X., An, Z., Gross, C., Chang, S.X., 2025. Forested lands have lower soil carbon priming effects than croplands in hedgerow agroforestry systems. Agriculture Ecosystems and Environment 394, 109921.
282. Yu, J., Miao, S.J., Li, T., Tang, Y.J., Zhao, Y.D., Qiao, Y.F., 2025. Straw Addition Induces Positive Priming Effects on Different Soil Organic Carbon. Eurasian Soil Science 58(13), 196.
283. Hou, X., Hu, P., Li, J., Wang, K., Zhang, W., 2025. Lithology modulates soil priming effect via resource limitations and bacterial community structure. Functional Ecology. https://doi.org/10.1111/1365-2435.70231.
